# Escape to Ferality: The Endoferal Origin of Weedy Rice from Crop Rice through De-Domestication

**DOI:** 10.1371/journal.pone.0162676

**Published:** 2016-09-23

**Authors:** Kimberly L. Kanapeckas, Cynthia C. Vigueira, Aida Ortiz, Kyle A. Gettler, Nilda R. Burgos, Albert J. Fischer, Amy L. Lawton-Rauh

**Affiliations:** 1 Department of Genetics and Biochemistry, Clemson University, Clemson, South Carolina, United States of America; 2 South Carolina Department of Natural Resources, Marine Resources Research Institute, Charleston, South Carolina, United States of America; 3 Department of Biology, High Point University, High Point, North Carolina, United States of America; 4 Department of Plant Sciences, University of California Davis, Davis, California, United States of America; 5 Department of Crop, Soil, and Environmental Sciences, University of Arkansas, Fayetteville, Arkansas, United States of America; Università Politecnica delle Marche, ITALY

## Abstract

Domestication is the hallmark of evolution and civilization and harnesses biodiversity through selection for specific traits. In regions where domesticated lines are grown near wild relatives, congeneric sources of aggressive weedy genotypes cause major economic losses. Thus, the origins of weedy genotypes where no congeneric species occur raise questions regarding management effectiveness and evolutionary mechanisms responsible for weedy population success. Since eradication in the 1970s, California growers avoided weedy rice through continuous flood culture and zero-tolerance guidelines, preventing the import, presence, and movement of weedy seeds. In 2003, after decades of no reported presence in California, a weedy rice population was confirmed in dry-seeded fields. Our objectives were to identify the origins and establishment of this population and pinpoint possible phenotypes involved. We show that California weedy rice is derived from a different genetic source among a broad range of AA genome *Oryzas* and is most recently diverged from *O*. *sativa* temperate *japonica* cultivated in California. In contrast, other weedy rice ecotypes in North America (Southern US) originate from weedy genotypes from China near wild *Oryza*, and are derived through existing crop-wild relative crosses. Analyses of morphological data show that California weedy rice subgroups have phenotypes like medium-grain or gourmet cultivars, but have colored pericarp, seed shattering, and awns like wild relatives, suggesting that reversion to non-domestic or wild-like traits can occur following domestication, despite apparent fixation of domestication alleles. Additionally, these results indicate that preventive methods focused on incoming weed sources through contamination may miss burgeoning weedy genotypes that rapidly adapt, establish, and proliferate. Investigating the common and unique evolutionary mechanisms underlying global weed origins and subsequent interactions with crop relatives sheds light on how weeds evolve and addresses broader questions regarding the stability of selection during domestication and crop improvement.

## Introduction

Understanding the evolutionary genetics of adaptation to human-mediated practices like small and large-scale production agriculture is critical to address global challenges including the security of food, fuel, bioproduct, and fiber production [1]. A central challenge in agriculture is to harness the genetic variation controlling key traits in crops to produce stable populations that can be planted, managed, and harvested effectively. Evolutionary models frame and explain the domestication, continued improvement, and management of cultivated plants. Examining these processes sheds light on the roles of selection and demography on genetic interactions of populations and species during adaptation [2]. During domestication and crop improvement, individuals are selected for predictable traits. The means and variances of these traits in breeding lines over generations depend upon the relative roles of genetics and the environment in shaping variation and the number of alleles at loci (and across linked regions) governing these phenotypes. Likewise, the additive genetic variance associated with a given domestication trait may control how easy it is to fix a population for a trait value, particularly for traits that are vastly different from wild or weedy close relatives.

Domestication is a selection process for adaptation to agro-ecological niches favorable for human use, harvest, consumption, and management. Historical gene flow between wild progenitors and domesticated plant populations ensures that cultivated varieties (cultivars) vary in their composition of domestication versus wild traits. Domesticated lines and wild relatives that can interbreed are common among plants [3] and animals [4]. Genome-wide studies of these interbreeding complexes help us understand how genetic introgression modulates adaptation and the maintenance of species boundaries in the face of gene flow [5–9].

Although weedy rice physiologically and phenotypically resembles cultivated rice, it differs in several important weedy traits, including seed shattering habit, seed dormancy, protracted emergence, and the presence of red pigmentation in the seed pericarp in many cases. Shattering furthers propagation of the weed because seeds scatter in the field before cultivated (non-shattering) rice is harvested. Variation in gene sequence and expression has been shown in many genes related to seed shattering, including *qSH1*, *sh4*, and *SHAT1* [10,11]. The shattering trait in weedy rice has been shown to re-evolve after fixation of the non-shattering *sh4* allele in its domesticated ancestors [12]. Additionally, QTL analysis indicates that shattering has re-emerged independently and is controlled by different genetic locations in weedy rice [13].

Variable seed dormancy makes control of weedy rice by crop rotation difficult due to the ability of weedy rice to remain dormant for extended periods in the field [14]. Protracted emergence patterns make control by chemical means difficult because late or early emerging individuals can escape herbicide applications [15]. Prolonged and highly variable emergence also makes control by non-chemical means, such as cover crops, difficult. Finally, weedy rice is commonly referred to as red rice when characterized by a red-pigmented pericarp. Contamination of commercial rice with pigmented red rice seed significantly lowers its commercial value [16].

Most traits distinguishing crop from weedy forms are determined by recessive alleles of major-effect loci [17]. A subsequent focus on the molecular evolution of genes important in de-domestication can guide our understanding of the tempo and process of evolution in weedy and feralized crop populations, but first we must examine the evolutionary origins and morphologies that characterize emergent weed populations. This knowledge informs agricultural management strategies that account for how weeds evolve and mitigate infestation. Understanding the genetic interplay underlying these processes will predict their directionality, identify traits for crop improvement in the face of new or changing environmental constraints, and outline ecosystem management strategies for sustainability.

Cultivated Asian rice (*Oryza sativa*) and its progenitor *O*. *rufipogon* are both diploid AA genome species, which facilitates introgression and the maintenance of hybrid feral forms (intermediate conspecific weedy rice). There are two cultivated species of rice: African rice (*O*. *glaberrima* Steud.), which was domesticated from the wild progenitor *O*. *barthii* in Africa, and Asian rice (*O*. *sativa*), which was domesticated from the wild progenitors *O*. *rufipogon* and *O*. *nivara* in Asia. Asian rice is classified under two major subgroups, *japonica* and *indica*. The *japonica* subgroup includes tropical *japonica*, temperate *japonica*, and aromatic rice, while the *indica* subgroup includes *aus* and *indica* rice. Rice cultivation in the US includes primarily tropical *japonica* cultivars in the Southern rice belt and temperate *japonica* in northern California. Recently, rice production in California has included ‘specialty’ varieties of temperate *japonica* rice (referred to as ‘gourmet rice’ herein). Although gourmet rice varieties are brought in, control of imported and specialty seed stocks in California has been tightly regulated to prevent the accidental introduction and dissemination of wild or weedy rice [18, 19].

Weedy rice interacts with rice in the US, mainly across the Southern rice belt in Arkansas, Louisiana, Mississippi, Missouri, and Texas. This weed most likely originated from early domesticated Asian rice (*O*. *sativa*) that reverted to wild/weedy traits (e.g. taller plants with awns, increased tillering, pigmented pericarp, and/or asynchronous flowering with the crop) and was later introduced into rice cultivation in the US [12, 20, 21]. In the southern US, there are two major weedy rice ecotypes that have been consistently well-defined, strawhull awnless (SH) and blackhull awned (BHA). SH and BHA weedy rice are most similar genetically to *indica* and *aus* rice varieties, respectively [21, 22]. Hybridization between weedy rice ecotypes and between weedy rice and cultivated rice has been shown to increase genetic diversity in these groups [21, 23]. Neither *indica* nor *aus* varieties were grown in the US at or before the time weedy rice was reported in southern US rice, indicating that both ecotypes arose in Asia and were brought in as contaminants of seed stocks during early rice production.

**Fig 1:**
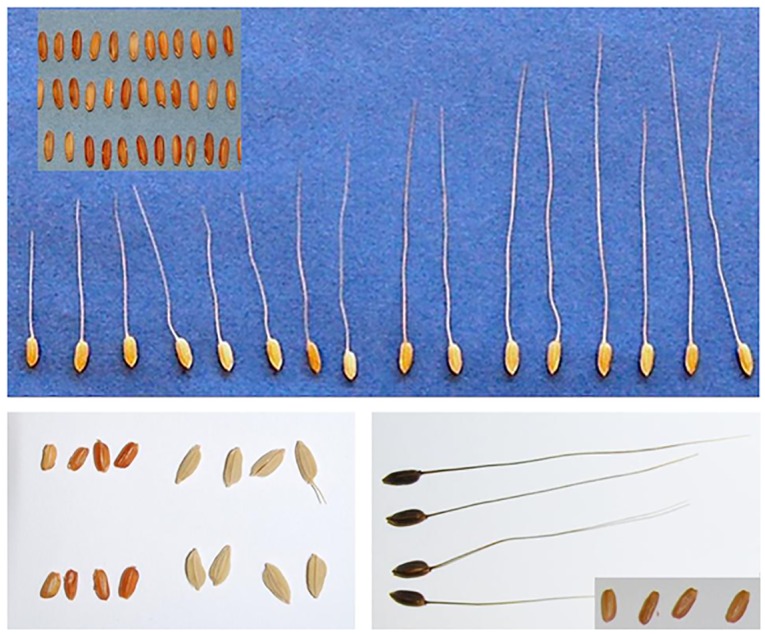
Grains of the California weedy rice ecotype and the southern weedy rice ecotypes. California weedy rice (top panel) with representative strawhull (bottom left) and blackhull (bottom right) weedy rice ecotypes. California weedy rice has straw to golden colored hulls and long straw colored awns. Strawhull weedy rice has short to no awns and straw colored hulls. Blackhull weedy rice has long, dark awns with a dark hull color. All three ecotypes have the characteristic red colored pericarp (wherein weedy ‘red’ rice derives its common name).

Rice cultivated in California is largely of the straw hull (SH) variety, while the weedy rice infesting this region is straw hull awned (SHA). Morphologically, California SHA weedy rice is distinct from both SH and BHA weedy rice in the southern US (as well as other global weedy rices), as it has a straw-colored hull with long awns (Fig 1). Moreover, California SHA weedy rice is morphologically distinct from cultivated rice in California as it has colored pericarp and fully developed awns. SHA weedy rice was widespread in California rice fields from the 1920s [24, 25] into the 1940s [26]. Bellue (1932) [25] proposed that California weedy rice in the early 1900s originated from contamination from other parts of the US (southern regions). These Asian tropical *japonica* varieties are not present in the US, and no evidence has supported southern US weedy rice de-domestication from the co-occurring cultivated rice they infest [27]. Swift management efforts through a direct, water-seeding system, herbicides, and certified seed mitigated infestations in the Sacramento Valley until complete elimination of weedy rice (SHA) in California in the 1970s [28]. California SHA weedy rice was eradicated until 2003 when a single field was infested. Since 2003, this weed has spread to several other fields in Colusa and Glenn counties. Possibilities for the origin of California weedy rice include hybridization between cultivated rice and other relatives, reversion of cultivated rice to weediness, or introduction of an already established weedy rice lineage by contamination of seed stock entering California. Because the southern US grows tropical *japonica* cultivars and California grows temperate *japonica* cultivars, contamination of seed stocks would most likely occur outside of the US.

Weedy rice is commonly referred to as red rice when characterized by a red-pigmented pericarp. Contamination of commercial rice with pigmented weedy red rice seed significantly lowers its commercial value [16]. Weedy rice is very similar to the cultivated crops with which it grows, both genetically and phenotypically [29]. The low genetic distance between cultivated, weedy, and wild forms maintains intermediates and in turn perpetuates hybridization between crop and weedy/wild rice. These similarities can result from the loss of crop-specific alleles in crops, resulting in weediness (*e*.*g*. feral or weedy rye (*Secale cereale* L.), hybridization between crops and wild relatives (*e*.*g*. radish *Raphanus sativus* L. and wild radish *Raphanus raphanistrum* L.), or by selection for phenotypic mimicry of the cultivated plant (*e*.*g*. barnyardgrass (*Echinochloa crus-galli* L.) growing in rice fields [30–32]. When domesticated plants and weedy plants are genetically compatible, hybridization can potentially transfer alleles for weedy characteristics to the cultivated populations and cultivar-specific alleles into weedy populations [23]. One important example of this is when herbicide resistance alleles move into weedy species that hybridize with resistant crops [33–35]. Indeed, interactions among crops and weeds can impact the adaptive potential of a weed to a new environment by simultaneously increasing genetic diversity in the weed and imparting alleles from the crop that are already suited to survival in an agro-ecosystem [29].

Although rice is a ‘model system’ for domestication studies and the evolutionary history of many global weedy rice ecotypes is well-established, the origin of this recently emergent weedy *Oryza* population in areas without endemic species (e.g. California) is poorly understood. In this study, we elucidate the origins of California weedy rice and attempt to identify morphologies that confer weediness in the de-domestication process. We used a genome-wide panel of 48 sequence tagged sites (STS), which are 400–500 bp portions of expressed genes that have already been sequenced in a thorough sampling of AA genome *Oryza* species [21]. The STS loci we use in this study are an established and effective tool in the rice community for recapturing the population structure of weedy rice, and represent an unbiased sample of genomic SNP diversity across diverse *Oryza*, including similar varieties [36]. Indeed, the data from these 48 STS markers enables quantification of nucleotide variation in weedy, cultivated, and wild rice, and enable the robust quantification of US weedy rice nucleotide variation and population structure (parsing well-defined and admixed populations) as well as the determination of which *Oryza* have contributed to US weedy rice genomes and the role of de-domestication in weedy rice evolution [36]. The Olsen *et al*. (2007) publication [36] provides thorough information as to how loci are distributed among the 12 rice chromosomes and the suitability of estimation of *F*_*ST*_ and all other genetic diversity parameters. The diverse panel of *Oryza* used in this study included wild species from Asia (*O*. *rufipogon* and *O*. *nivara*), Africa (*O*. *barthii*), Central America (*O*. *glumaepatula*), and Australia (*O*. *meridionalis*), along with cultivated Asian rice (*O*. *sativa*: tropical *japonica*, temperate *japonica*, *aus*, *indica*, and aromatic) and cultivated African rice (*O*. *glaberrima*). To these sequences, we added sequence information at the same loci for weedy and cultivated rice collected from California in order to identify the origin of this newly established weedy population. The population divergence history and variance in the many phenotypes used by the International Rice Research Institute (IRRI) [37] to characterize rice life history stages are unknown in this recently reported weedy population. We show that weedy rice in California is genetically and morphologically distinct from other weedy, wild, and cultivated rice groups included in our sampling. Hybridization as the mechanism of origin is unlikely in this case due to the low level of sequence diversity, uniform haplotype grouping assignment within the California weedy group, and complete homozygosity at all loci for all individuals. Coalescent model-fitting indicates that California weedy rice diverged most recently from temperate *japonica* cultivars which are grown in California, possibly involving a recent regression of cultivated rice back to a weedy form since establishment in the US. The picture that emerges from our study is that, despite low diversity, weedy rice can harbor significant trait variance and be morphologically distinct from its domesticated progenitors. Understanding how and why crops turn weedy and the dynamics of feral forms in production agriculture will help ameliorate crop-weed competition, reduced yield and quality, contamination of harvested grain, and disease reservoirs due to these weeds.

## Materials and Methods

### Plant material

Mature seeds were collected from weedy rice plants growing in four fields in northern California in 2008. Collection of weedy rice seed was done with the help of rice extension agents, who obtained permission from growers to sample their fields. The elimination of weedy rice in California for decades prior to this recent discovery of weedy populations was made possible by the cooperation of growers. No endangered species were involved nor impacted by this activity.

For genotyping, we included a total of 27 weedy rice individuals and 12 cultivars (Table 1 and Table A in S1 File). Cultivars that we added to the existing STS sequence dataset were all temperate *japonica* grown in California.

**Table 1:** GPS coordinates of the four fields reported to be infested with SHA weedy rice in California, and the number of weedy rice plants sampled in each field for genetic analyses.

Field	GPS Coordinates	Weedy Rice Samples Taken (N = 27)
CRR1	Lat. 39.2332349; Long. -122.219323	8
CRR2	Lat. 39.6023524; Long. -122.125835	5
CRR3	Lat. 39.60227836; Long. -122.121612	5
CRR4	Lat. 39.60379276; Long. -122.099699	9

### Scoring of morphological traits

A collection of morpho-phenotypic traits was scored for both cultivated and weedy rice in California (Table A in S1 File). Twenty seven California weedy rice plants were sampled from the four rice fields in the state reported to be infested with weedy rice. Seventy-nine once-selfed lines (offspring) from the field collected mother plants were grown in the U.C. Davis outdoor facilities for phenotyping. Approximately three offspring lines were obtained from each original California weedy rice line (mother plant) collected from the field. Only certain traits that were applicable to field-collected “mother” plants at harvest—such as grain size—were used in the analysis to incorporate the most representative features of this emergent weed in the field.

Germinated seeds were transplanted on April 18, 2007, to 22-liter pots filled with saturated soil and placed inside basins. Fertilizer was added following field recommendations. Seedlings were thinned to one per pot soon after establishment and when seedlings reached the 3- to 4-leaf stage of growth, the basins were flooded as in a paddy field. Pots were spaced 50 cm apart and arranged in a randomized complete block design with six replicates per accession. Morphological traits evaluated in this study and measurement methods were based on rice descriptors for morpho-agronomic characterization published by the International Rice Research Institute [34].

### STS gene sequence analyses

Twenty-seven weedy rice plants were chosen for genotyping along with 12 accessions of temperate *japonica* varieties that are cultivated in California. Leaf tissue from the outdoor grown plants was excised and desiccated for shipment to Clemson University for DNA extraction. DNA was extracted from desiccated leaf tissue using the Macherey-Nagel NucleoSpin 96 Plant DNA extraction kit (Düren, Germany). Purified genomic DNA was diluted 2:1 in nuclease-free water for polymerase chain reactions (PCR). PCR was carried out using standard conditions to amplify 48 (400-500bp) gene fragments selected by [21] from 111 sequenced tagged sites (STS) developed by [38]. PCR products were checked by gel electrophoresis and cleaned up using Exonuclease and Antarctic phosphatase treatment (New England Biolabs^®^ Inc., Ipswich, MA, USA) following the method described in [39]. Direct sequencing in both the forward and reverse directions was carried out by the Clemson University Genomics and Computational Biology Laboratory.

Sequences were assembled into contiguously aligned sequence ‘contigs’ and assigned quality scores using Phred and Phrap. Contigs were aligned and inspected visually for quality and heterozygous sites in BioLign version 4.0.6.2 (Tom Hall, http://en.bio-soft.net/dna/BioLign.html). Heterozygous base calls were randomly assigned to two pseudo-haplotypes, which were then phased using PHASE version 2.1 [40–41]. Due to low levels of heterozygosity in the data set, haplotypes were inferred with very high probabilities and consistency across five runs. All sequences have been submitted to NCBI GenBank (*O*. *sativa* accession numbers KT441140-KT443009). Phased haplotypes were aligned with sequences obtained from [21]. These additional sequences consist of the same 48 STS loci for a broad range of AA genome *Oryza* species including 58 weedy rice accessions sampled over a 30 year period from Arkansas, Louisiana, Mississippi, Missouri, and Texas. Also included in this dataset are sequences from the major cultivated groups from Asia (*O*. *sativa indica*, *aus*, tropical *japonica*, temperate *japonica*, and aromatic) and Africa (*O*. *glaberrima*), as well as wild species sampled from Asia (*O*. *rufipogon* and *O*. *nivara*), Africa (*O*. *barthii*), Central America (*O*. *glumaepatula*), and Australia (*O*. *meridionalis*) [21].

### Data analyses

#### Genetic structure and divergence

Summary statistics for each STS locus including nucleotide diversity at silent sites (*π*) using the Juke’s Cantor correction [42], Watterson’s *θ* at silent sites [43], number of segregating sites *S*, and Tajima’s *D* [44] were calculated in DnaSP version 5.0 [45]. Arlequin version 3.5 [46] was used to calculate pairwise *F*_*ST*_ and *Ф*_*ST*_ estimates with 10,000 permutations to assess significance. Bonferroni corrections were used to determine *P*-value cutoffs. Recombination break points in each locus were determined using the four gamete test [47] in SITEs [48].

The population-mutation parameter *F*_*ST*_ is an estimate of genetic divergence within and between groups and was used to test for the extent of genetic differentiation. To better estimate divergence between California weedy rice and other rice groups, the population mutation parameter *Ф*_*ST*_ was used, which is similar to *F*_*ST*_ but uses distances between haplotypes, not just haplotype frequencies. Genetic diversity was measured by computing the average nucleotide diversity (*θ*_*π*_), total number of segregating sites, and Watterson’s *θ*_*w*_ within each field as well as within all fields combined.

Population structure was inferred using InStruct [49], which was designed to allow for inbreeding by not assuming Hardy-Weinberg equilibrium within populations. Using STRUCTURE [50] for inbreeding populations results in inappropriately higher rates of inferred splitting between populations (*i*.*e*. infers too many subpopulations). Five permutations for each number of populations (*K*) were set from 1 to 22 with 500,000 steps and a burn-in period of 100,000 steps. InStruct runs were completed on the Clemson University Condor computing cluster. Log likelihoods for each run were compared to determine the best fit *K* value. Distruct version 1.1 [51] was used to create the graphical display from the results obtained with InStruct [49].

Isolation with Migration modeling (IMa) [52–53] was used to test for best fit models of isolation-migration and simultaneously estimate effective population sizes (N_*ef*_), migration between populations (N_*ef*_*m*), ancestral population size (N_*ef*_A) and time since divergence (T). California weedy rice was compared on a pairwise basis to California cultivated rice (temperate *japonica*), strawhull (SH) weedy rice, blackhull (BHA) weedy rice, *O*. *rufipogon* (from three geographic regions) and *O*. *nivara*. Recombination was only detected in *O*. *rufipogon*, so the longest non-recombining blocks were only utilized in the comparisons including *O*. *rufipogon*. Each comparison was run in M-mode with wide value cutoffs for all parameters to determine where posterior probability distributions ranged. After the initial run, three runs were conducted with different random number seeds and smaller cutoff values that were based on the distribution of parameter values from the first run. All runs had 100,000,000 MCMC steps after a burn-in of 100,000 steps. Each run had 10 chains with a mixing rate of five chain swaps per step. All three M-mode outputs were checked for convergence and L-mode runs were conducted on the tree files to test nested models. The maximum likelihood estimates were scaled into demographic values based on a mutation rate of 1 × 10^−8^ and a generation time of one year, as done with previous work [21], based on [54, 55]. All IMa runs were computed on the Condor cluster at Clemson University using primarily an extensive web-enabled system to simultaneously manage and monitor performances of each set of input priors. Use of a cluster allowed for more than 28 simultaneous runs, where priors could be checked and adjusted as needed.

#### Multivariate analysis of trait variance

The goal of these phenotypic analyses was to elucidate genotype-phenotype relationships between California *Oryza* cultivar and weedy rice ecotypes. Thus, we determined the most influential phenotypic footprints of rapid divergence in domestic and wild-like traits of rice and its conspecific weed within the California floristic province. To characterize trait variability and by extension morphological relationships among domesticated and weedy rice ecotypes in infested fields, we quantified phenotypic diversity by first describing the variance partitioning of weedy populations and comparing the adaptive traits which characterized weedy rice to those that defined cultivars. To more accurately characterize dimensionality in weedy or feral rice morphology, a subset of unique gourmet varieties were added to the medium-grain cultivars in the rice dataset for the phenotypic diversity analyses.

Qualitative descriptors were transformed using the PRINQUAL procedure of SAS with the OPSCORE option for optimal scoring [56] and MONOTONE option for monotonic preservation of order. Principal Components Analysis (PCA) with maximum total variance (MTV) was performed on the combined quantitative and transformed qualitative descriptors. The variables describing cultivars were reduced by eliminating any that did not vary by descriptive statistics (mean and mode) and then using both random and *a priori* sampling to preserve group partitioning while identifying the eigenvectors which most clearly separated groups. UPGMA (Unweighted pair group method of arithmetic averages) hierarchical clustering using the CLUSTER procedure of SAS was performed to confirm separation of clusters on PCs (by comparing mean variance; significant differences were considered when *P ≤* 0.05) and to generate a dendrogram using average Euclidean distances. Qualitative transformations, PCAs, and MANOVAs were executed in SAS^®^ Version 9.3 (SAS Institute Inc., Cary, NC, USA 2002–2010). Eigenvector biplot graphics and hierarchical clustering dendrograms were generated in JMP (JMP Pro 10 SAS Institute Inc., Cary, NC, USA 1989–2010).

## Results

### Genetic diversity and divergence of California weedy rice

Average estimates of genetic differentiation (*F*_*ST*_) between weedy rice in California rice fields are very low, ranging from 0 to 0.0026 (Table 2). There are no significant differences in *F*_*ST*_ estimates for any of the 48 loci. The highest *F*_*ST*_ estimate was 0.077, between CRR1 and CRR4 at STS085. These low values indicate no population structure and no divergence of weedy rice in the fields sampled, which supports the appropriateness of a genetic diversity assessment for California weedy rice (see Tables D and F in S1 File for genetic diversity indices for all STS loci tested among all rice groups). Measures of genetic diversity (θ_*π*_ and Watterson’s θ_w_) for California weedy rice within each field as well as for weedy rice within all fields combined are also very low (Table 3), consistent with a recent founder event, or strong population bottleneck. These values are a full order of magnitude lower than what was calculated for strawhull (SH) and blackhull (BHA) weedy rice ecotypes collected from the southern US [21]. Due to the lack of population substructure and low genetic diversity, we placed all California weedy rice into one group for the remaining analyses.

**Table 2:** Average pairwise *F*_*ST*_ estimates between weedy rice sampled in four California fields.

Field	CRR1	CRR2	CRR3	CRR4
CRR1		0^b^ (0, 0.025)	0 (0, 0.025)	0 (0, 0.077)
CRR2	0.0005^a^		0	0 (0.012)
CRR3	0.0005	0		0 (0, 0.012)
CRR4	0.0026	0.0003	0.0003	

Bottom diagonal (a) are mean pairwise *F*_*ST*_ estimates across 48 loci, top diagonal (b) are median pairwise *F*_*ST*_ estimates with range in parentheses. All values were estimated in Arlequin.

**Table 3:** Nucleotide sequence diversity of California rice by field and for total data set.

	Total # SegSites	*θ*_*π*_ /Kb	*θ*_*w*_ /Kb
CRR1	4	0.0135	0.0560
CRR2	0	0.0000	0.0000
CRR3	0	0.0000	0.0000
CRR4	2	0.0325	0.0452

Total number of segregating sites (SegSites) is across 48 STS loci. Average nucleotide diversity at silent sites per 1000 base pairs (*θ*_*π*_ */*Kb). Average Watterson’s *θ* at silent sites per 1000 base pairs (θ_*W*_ /Kb).

Values for average population differentiation estimates (*Ф*_*ST*_) across all 48 loci (Table 4) indicate high divergence between California weedy rice and all other sampled groups. The lowest mean value is with *O*. *rufipogon* collected from Southeast Asia (*Ф*_*ST*_ = 0.3639). Taking the median values across the 48 loci allows better understanding of the patterns across all loci. The lowest divergence was between California SHA weedy rice and BHA and SH; median *Ф*_*ST*_ values (0.0152 and 0.0275, respectively) indicated that for at least half of the loci tested divergence was an order of magnitude lower than the mean estimates (0.4247 and 0.4462, respectively). This indicates that the mean *Ф*_*ST*_ is high due to divergence at a few loci, and that California weedy rice does share some similarity to weedy rice from the southern US at several loci.

**Table 4:** Average population differentiation estimates (*Ф*_*ST*_) between California weedy rice and other rice groups.

CA weedy rice versus:	Mean *Ф*_*ST*_	Median *Ф*_*ST*_
CA temp. *japonica*	0.5496	0.8638
SH weedy	0.4462	0.0275
BHA weedy	0.4247	0.0152
*O*. *rufipogon* China	0.4375	0.3771
*O*. *rufipogon* India	0.4094	0.3593
*O*. *rufipogon* SE Asia	0.3639	0.3075
*O*. *nivara*	0.5956	0.6992
*O*. *glumaepatula*	0.7146	0.9843
*O*. *barthii*	0.7894	0.9601
*O*. *glaberrima*	0.7658	1.0000
*O*. *meridionalis*	0.9636	1.0000

Estimates are averaged across 48 STS loci.

### Population structure of California cultivated and weedy rice

**Fig 2:**
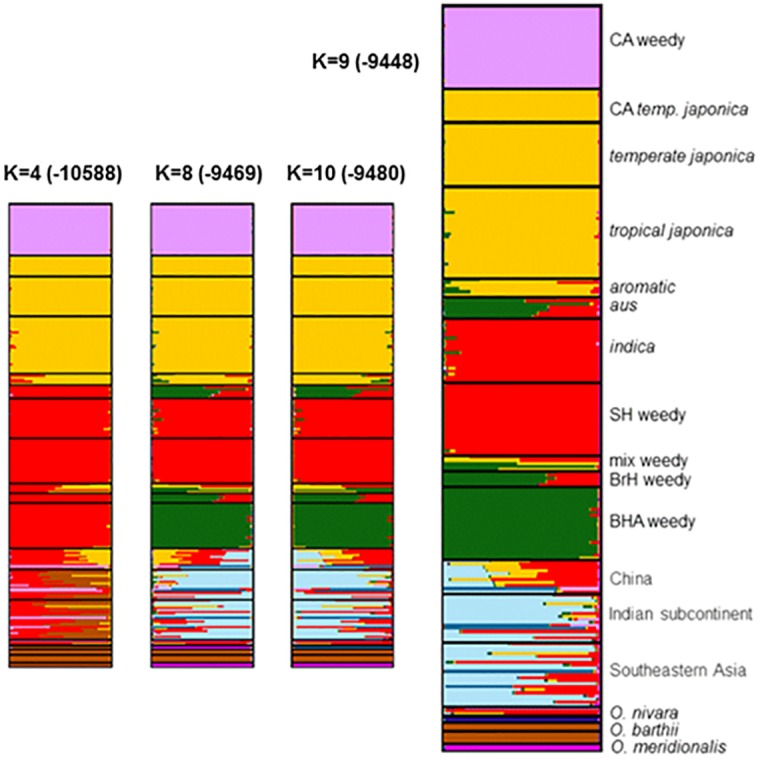
Population structure as inferred from the best fit number of groupings in InStruct. Each color indicates genetic membership of the specified number of groups (*K*). The best fit model had *K* = 9, shown on the right. *K* = 8 and *K* = 10 are included for comparison. *K* = 4 was the lowest *K* value maintaining California weedy rice as a separate group. Ln likelihood scores for each *K* value are shown in parentheses, and likelihood scores for *K* = 1 through *K* = 22 are in Table E in S1 File.

Population structure, as inferred by InStruct [46], was used to help determine the potential origin of California weedy rice genotypes from a global sample of *Oryza* species. The best fit model is a population number or *K* value of 9 (Ln Likelihood = -9448). Shown in Fig 2 are groupings with *K* = 4 (Ln Likelihood = -10588) and *K* = 8 (Ln Likelihood = -9469) through *K* = 10 (Ln Likelihood = -9480). California weedy rice remains a distinct group down to *K* = 4, where all other weedy rice individuals are grouped together with *indica* and *aus* cultivated varieties. At *K* = 9, membership of clusters was consistent with previous work [18]; however, temperate and tropical *japonica* accessions were not resolved. These remain unresolved for all *K* values up to 22 (Table E in S1 File), indicating that there is not enough sequence variation to separate these groups. Previous work with the dataset was able to resolve these two groups (not shown); however, the addition of California weedy rice impedes resolution.

### Demographic history of California weedy rice

**Fig 3:**
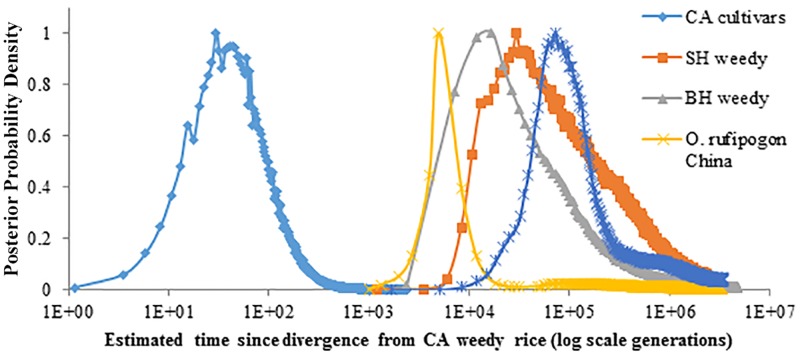
Posterior probability densities for time since divergence from California weedy rice estimated by the isolation-with-migration model. Time axis is on a log scale to include all comparisons (among California US cultivars, southern US strawhull (SH) and blackhull awned (BHA) weedy rice, and *O*. *rufipogon* from China and India).

**Fig 4:**
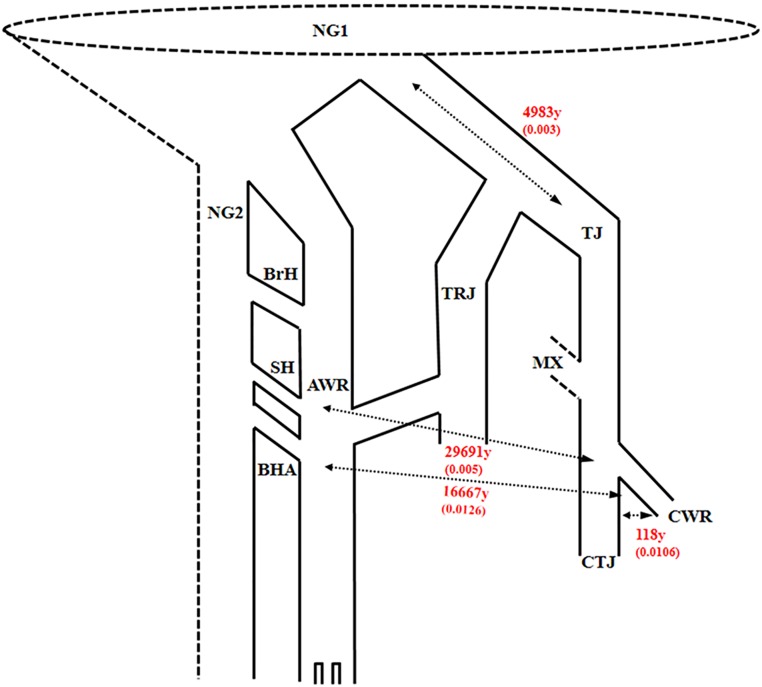
Relative divergence times in generations (G) (per Table 5). Ancestral gene flow/migration (NeM) from each likely source into California weedy rice are in parentheses. Labels are as follows: *Oryza sativa* ancestral global pool, including Chinese *O*. *rufipogon*, aromatic, *barthii*, *meridionalis* (NG1), *O*. *sativa* global sources including Indian *O*. *rufipogon*, *nivara*, *sativa* aus, other indica-like *Oryza* (NG2), temperate *japonica* (TJ), tropical *japonica* (TRJ), brownhull (BrH), blackhull (BHA), or strawhull awnless (SHA) Arkansas weedy rice (AWR), California temperate *japonica* (CTJ), SHA (strawhull awned) California weedy rice (CWR), and mixed genotypes (*e*.*g*. red pericarp African *O*. *glaberrima*) (MX).

The most recent divergence of California weedy rice from other *Oryza*s is from California rice cultivars (temperate *japonica*), which was estimated at about 118 generations ago (posterior credible interval falls between 118 and 218,000 generations ago). The other divergence estimates were over an order of magnitude older (Fig 3 and Table 5). Interestingly, both SH and BHA weedy rice from the southern US have very old divergence estimates: approximately 30,000 (posterior credible interval is between 5938 and 1,713,777 generations) and 17,000 (posterior credible interval is between 2,381 and 2,683,333 generations), respectively. These numbers are likely inflated compared to the reported origin of domesticated rice approximately 10,000 generations ago due to interactions among other closely related genotypes. This follows work examining model testing performance of IMa under several scenarios, which showed that divergence estimates inflate when gene flow from other populations is included in the model [57]. A model of relative divergence times shows a shallow, recent coalescence of California weedy rice and California crop rice alleles, whereas SH and BHA southern US weedy rice and Chinese *O*. *rufipogon* show a much older divergence from California weedy rice (Fig 4). Migration estimates between California weedy rice and all other groups were quite low, with higher estimates of migration into California weedy rice in all cases. Indeed, these data should not be interpreted as absolute numbers but instead as relative values. Any overestimation of the generation values (e.g. inflation) could otherwise indicate that the divergence actually happened even more recently. The effective population size for California weedy rice is very small in all cases, supporting a recent founder event or bottleneck.

**Table 5:** Co-estimated demographic parameters for California weedy rice compared to a second population as inferred in the best fit isolation-with-migration model (IMa).

Pop 2*	Ne*** CA weedy	*Ne*2	*Ne* Ancestral	NeM**** from CA weedy	*NeM* into CA weedy	Time (G)
**CA cultivars**	**6** (0, 24)	**65** (6, 194)	**3476** (647, 55253)	**0.0011** (0.0001, 0.01)	**0.0106** (0.002, 0.05)	**118** (118, 218000)
**SH weedy rice**	**0** (0, 6)	**24** (6, 83)	**6** (6, 17654)	**0** (0, 0.02)	**0.005** (0.0001, 0.03)	**29691** (5938, 1713777)
**BHA weedy rice**	**6** (0, 24)	**48** (18, 167)	**4190** (1179, 194060)	**0.0029** (0.001, 0.01)	**0.0126** (0.003, 0.04)	**16667** (2381, 2683333)
Wild rice China**	**0** (0, 25)	**814** (324, 2342)	**13364** (4676, 13364)	**0** (0, 0.104)	**0.003** (0.0002, 0.2)	**4983** (1661, 27779070)

Point estimates are in bold typeface, and the high and low 90% Bayesian posterior probability densities are in parentheses.

*Second population used for comparison to CA weedy rice.

**Wild rice is *O*. *rufipogon* sampled from China.

*** *Ne* is the effective population size.

****NeM is the effective migration rate between populations (average number of migrants per generation G).

### Weedy (wild-like) traits and de-domestication

California weedy rice differs morphologically from other southern US weedy rice ecotypes. California weedy rice has a straw-colored hull with long awns (SHA) (Fig 1), whereas only 7% of SH weedy rice in the southern US has awns [58]. Nevertheless, California weedy rice shares important weedy traits with those of southern US weedy rice including high seed shattering and a red-colored pericarp in addition to tall stature and high tillering habit [58].

### Trait variance distinguishes California weedy rice from cultivated rice

**Fig 5:**
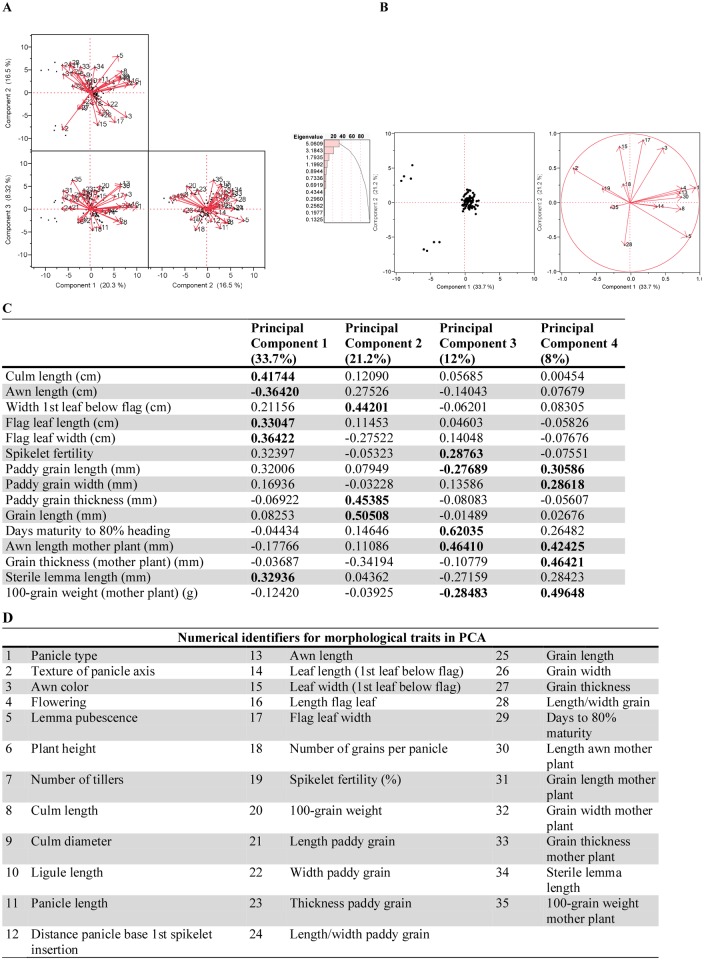
California weedy and cultivated rice phenotypic trait decomposition. 5A. PCA of California weedy and crop (medium-grain and specialty) rice. Highly correlated traits and traits with no variation excluded. 5B. PCA biplot of the refined PCA analysis of California weedy, medium-grain crop, and specialty crop rice traits which are key players in separating the three clusters. 5C. Eigenvector values for a refined California weedy and crop rice dataset of 15 traits with highly correlated measurements and those with no variation excluded (from 5B). 5D. List of traits corresponding to the numeric labels (1–35) of PCA vectors in 5A-5C. The length and direction of each vector indicate the strength and type (positive or negative) of the correlation between specific traits and the PCs.

Principal components analysis (PCA) reduced the set of observed variables for California weedy and cultivated rice by loading them on orthogonal lines of fit based on contributions to variance. No variation was observed amongst weedy and cultivated rice for leaf texture and angle, ligule shape, ligule color, ligule pubescence, auricle color, node color, or panicle secondary branching, so these traits were excluded from the analysis. When multiple traits represented the same metric, we chose the variable with the highest eigenvector value (Fig 5; Table B in S1 File) to represent each group or suite of highly correlated traits, although each group member or variable has an impact when describing the underlying mechanism responsible for phenotypic selection differences between the cultivar and weedy rice in California.

Importantly, pericarp (bran) color clearly distinguishes weedy from cultivated rice in California (weedy rice has reddish brown to purple pericarp whilst domesticated rice has brown pericarp), but is not highlighted in the dimension-reducing PCA (as it is in the MANOVA-confirmed morphogroups) because it was scored as a qualitative (not quantitative) trait following International Rice Research Institute descriptor guidelines (IRRI, [37]. As a qualitative character, pericarp color was scaled to be analyzed in conjunction with discrete, quantitative variables which were more influential compared to the optimally scored, and monotonically transformed, qualitative categorical data in the PCA. There was, however, variation in pericarp pigmentation that was not present in most of the other transformed qualitative data, which was revealed in the hierarchical clustering analysis (Figure A and Table C in S1 File).

The remaining phenotypic traits included vegetative growth habit characters, reproductive morphologies, and yield metrics related to grain morphology. Principal components analysis was conducted on these informative traits, excluding highly correlated variables (Fig 5A). A second PCA (PCA 2) was performed on a reduced dataset, which included the five traits with highest eigenvector values for each principal component (PC1, PC2, and PC3) in the initial analysis (Fig 5B and 5C). Principal Components 1 (PC1, 20.32%), 2 (16.53%), and 3 (8.32%) together account for 45.18% of the total cumulative variance in cultivated and weedy rice in the first PCA (Fig 5A). Traits most greatly discriminating California weedy from cultivated rice include panicle type, leaf width (first below flag leaf), flowering (early, intermediate, late), awn color, and culm length (plant height) (PC1); lemma pubescence, texture of the panicle axis, length of the first leaf below the flag leaf, length/width ratio of grain, and width of flag leaf (PC2); 100-grain weight of the field-collected mother plant, awn length of the field-collected and offspring plants, spikelet fertility (seed set), and grains per panicle described most of the variation along PC3. Because we were interested in the contribution of suites of traits describing each statistically significant orthogonal vector, the 15 traits contributing most to variance along the first three PCs in PCA 1 were subjected to a second analysis. In PCA 2 of “key discriminating traits,” principal components 1 (PC1, 33.73%), 2 (21.23%), 3 (11.96%), and 4 (7.99%) account for 74.91% of the cumulative variance in the phenotype of California weedy rice (Fig 5B). Since components or dimensions with an eigenvalue greater than one are statistically relevant to the result (termed the Guttman-Kaiser cutoff criterion), we report four principal components for this second PCA [59–60].

### Crop (domestication) traits retained in California weedy rice

Traits in California weedy rice plants that mimic both medium-grain and gourmet specialty cultivars include erect leaf angles, cleft ligule shape, culm strength (most plants lean), green node color, panicle exertion (panicle exerts well above the collar of the flag leaf blade), and ligule pubescence (hirsute or hairy for all California weedy rice ecotypes). Crop traits in weedy rice specific to co-occurring medium-grain cultivars include basal leaf sheath color (light green in medium-grain crop and weedy rice) and hull color (strawhull). Crop traits grouping weedy rice with gourmet specialty varieties include intermediate tillering or spreading growth habit (medium-grain cultivars are erect) (Table 6 and Fig 5). These crop-like traits are presumably carried by the immediate progenitors of the weedy rice, the crop, and have not been lost by the crop. When shared between weedy and cultivated rice, some of these traits (such as identical hull color) could reinforce weed persistence and adaptation to cultivation by visually disguising the weed, thus preventing detection.

**Table 6:** Traits conferring phenotypic identity to novel weedy rice and co-occurring cultivated rice in California.

Phenotype	California Weedy Rice (CWR)	California Cultivated Rice (medium-grain CCR1)	California Cultivated Rice (gourmet, CCR2)
Pericarp	Pigmented (red-reddish brown)	Non-pigmented (white)	Pigmented (brown)
Spikelet Fertility	Low (76% or lower)	High (78–87%)	High (78–87%)
Awnedness	Long, fully developed awns	Awnless	Very short awns
Stature	Tall (110 cm average height)	Short (77–104 cm average height)	Short (77–104 cm average height)
Culm Habit	Acute culm angle	Spread habit (obtuse culm angle)	Spread habit (obtuse culm angle)
Tillers	High number of tillers	Fewer tillers	Fewer tillers
Panicles	More grains (22+ per panicle); More panicles, opened	Compact panicles; Fewer grains (10–17 per panicle)	Compact panicles; Fewer grains (10–17 per panicle)
Texture	Hispid-scabrous to tip of panicle	Smooth panicles	Smooth panicles
Flowering	Intermediate to late (protracted)	Early to intermediate	Early to intermediate
Internode Color	Golden	Green	Green

Traits conferring phenotypic identity to novel weedy rice and co-occurring cultivated rice ecotypes in California. This phenotype summary is generated from the comparison of morphogroups based on principal components analysis, hierarchical clustering, and multivariate analysis of variance.

### Wild-like traits distinguishing weedy rice from cultivated rice in California

Wild-like traits in California weedy rice traits exhibit high variance and differentiate this nascent feral population from both medium-grain cultivated varieties M-104, M-202, M-204, and M-205 as well as gourmet varieties in several ways. Compared with medium-grain cultivars, California weedy rice has a purple pericarp, long fully developed awns, lower seed set but with more grains per panicle, open panicles with scabrous texture, more tillers and panicles, spreading growth habit, long culm (100 cm) with gold internodes and delayed and extended flowering period (Table 6). The medium-grain cultivars in California have brown pericarp; short awns, (gourmet varieties are awnless), high seed set, less tillers and panicles than weedy rice, have compact panicles, are less than 100 cm tall, flower earlier than weedy rice, and have erect culms. Distinct clusters within the California weedy rice population can be resolved by a multivariate analysis of variance (MANOVA).

UPGMA cluster analysis confirmed morphogroups (California weedy, California medium-grain cultivars, and California specialty cultivars) based on the key partitioned traits identified through PCA (Table 6). The number of clusters N was given as three for several clustering methods (*e*.*g*. average, K-means, centroid, ward). More clusters could be resolved amongst the weedy rice, but an additional split did not offer more information when co-occurring rice cultivars were included (Figure A and Table C in S1 File).

## Discussion

### The unique, independent origin of weedy rice in California

Weedy rice in California is a newly established and distinct group in the USA. Isolation with migration (IMa) modeling suggests that California SHA weedy rice diverged from California rice cultivars approximately 118 generations ago. The relatively recent divergence, distinct morphology, and small genetic relatedness with other US weedy rice indicate that this unique population has evolved separately from a cultivated ancestor.

The recent origin of California weedy rice suggests that the population has differentiated since the establishment of rice cultivation in California and is in the early stages of segregating weedy traits, such as more tillers, extended flowering, and pigmented pericarp. Across all loci, we find no haplotypes in California cultivated rice that are not present in other *japonicas* (data not shown). Further, there are no additional shared polymorphisms between California weedy rice and other *japonicas* that are not shared between California weedy and California crop rice. California weedy rice either (1) diverged from japonicas outside of the US and was brought in to California after becoming weedy or (2) diverged from California *japonica* cultivated in California. The former argument that this weed was brought in is highly unlikely due to strict laws in rice seed import into California. Regardless, California weedy rice is distinct from the other US weedy rices and our coalescent IM estimates point to a recent de-domestication from the California *japonica* line. Indeed, while the generation values may be inflated (a common issue with IMa estimates), this does not necessarily mean that the estimate is greater than it should be. The relative values are indicative of the relatively recent divergence, which was our objective with this analysis. This result does not demonstrate definitively that time since divergence between California cultivated and weedy rice is different from that between BHA and California weedy rice; however, it is clear that California cultivated rice and California weedy rice have different origins, and more importantly, that the divergence of California weedy rice is more recently from cultivated rice in the same area than that from all other *Oryza* groups investigated. Moreover, while California weedy rice shares some similarity to weedy rice from the southern US at several loci as expected with a similar genetic background, divergence at few specific loci highlights changes required for California weedy rice to thrive in this novel agricultural system. If weedy rice in California is newly derived from a temperate *japonica* crop ancestor in current commercial fields, this suggests that new feral ecotypes can evolve anywhere rice is under domestication.

Our population genetic diversity analysis is consistent with recent establishment of the weed from a few individuals: low genetic diversity within and between California rice cultivars where weedy rice infestations occurred. Pairwise divergence estimates indicate high genetic divergence between California weedy rice and other groups. We examined the possibility that gourmet rice varieties (which include red pericarp pigmented types) could have been the source of California weedy rice because it may be an established cultivated or wild-weedy rice originating outside of North America that recently colonized California [22]. Thus, we sequenced 12 informative STS loci in red colored pericarp gourmet rice varieties grown in California and found no shared haplotypes with California weedy rice (data not shown). In addition, California weedy rice is distinct morphologically from gourmet varieties scored in this study (Table 6). While both California weedy rice and its putative domestic progenitor share straw hull color, phenotypic traits that differentiate California weedy red rice from cultivars include purple pericarp, increased plant height, longer leaves, greater tillering capacity, protracted flowering time, more grains per panicle, long awns, and seed shattering.

The de-domestication origin of weedy rice direct from rice cultivars is not unique to this study, as at least two cases of *O*. *sativa* f. *spontanea* have been documented in the Guangdong and Liaoning provinces of China [61–62]. Weedy rice in California is derived from rice cultivar in a smaller-scale rice growing region isolated from wild relatives and compliant with very strict guidelines for seed purity as well as tracking and reporting infestations.

An earlier US weedy rice population genetics study by Londo and Schaal [22] included microsatellite data for a single California weedy rice genotype, RR28, collected in California. Sequence analysis of the p-VATPase region of this genotype indicated that it was a unique, private haplotype genetically most similar to *O*. *rufipogon*—specifically *O*. *rufipogon* A100945-1 from southeast Asia used in crossing trials in California during the late 1970s [63]. These M-101 crosses with *O*. *rufipogon* accessions produced weedy rice (crop-wild hybrid origin) with red pericarp and shattering seeds to be used as a stem rot- resistant parent in breeding programs. California weedy rice RR28 appears to be a unique variant not found in our collection of thorough California weedy rice sampling. Moreover, [22] included only one California cultivar (M-205) in their study. This recently released cultivar is likely not the exclusive source of domestication alleles, so our study was designed to capture a comprehensive set of crop alleles from a wider range of California cultivars. Londo and Schaal [22] reported that RR28 shares alleles with NSGC 5936 (*O*. *rufipogon* A101510 from India) and California cultivar accession M205. The California ‘red’ rice specimen in our study, from the same county as the one used by [22], are four gourmet varieties (cultivars) with red bran. A single genotype cannot explain the population-level evolutionary history of weedy rice in California. We employed an extensive, broad sampling of wild, weedy, and cultivated genotypes to test the hypothesis of a wild/domestic hybrid in California. This sampling approach enabled us to examine how global and gourmet specialty rice sources have contributed to the localized evolution of weedy rice. The intermediate morphology of California weedy rice alone—strawhull awned (SHA) and pigmented pericarp—does not necessarily indicate a crop-wild hybrid origin. Indeed, we found no evidence to suggest an escaped *O*. *rufipogon* source of weedy rice (Tables 4 and 5).

From an evolutionary standpoint, the amount of morphological variation exhibited by this emergent lineage of weedy rice demonstrates its rapid adaptive potential and ability to persist in agroecosystems. Zhang *et al*. [62, 64] also used UPGMA cluster analysis and principal component analysis (PCA) to show that a spontaneously emergent weedy rice lineage is more closely related to rice cultivar grown in the sample field than with other neighboring cultivars or weedy varieties, supporting a de-domestication hypothesis that weedy rice can be derived from cultivated rice as we show in this study (Tables 4 and 6). The possibility that a widely cultivated species has a propensity to feralize under selection pressure variation has implications for crop management and necessitates further investigation on both the agroecological and molecular evolutionary levels (Kanapeckas *et al*., *in prep*.).

### The impacts of weed diversity and crop mimicry on management

Morphological traits used to classify major ecotypes of US weedy rice (in California and the southern US), including awnedness, plant height and culm abundance, seed shattering, spikelet fertility (seed set), flowering, grain weight, and leaf size, are readily identifiable in the field (see Table 6 and Tables A and C in S1 File). However, determining the evolutionary pathways to weediness is imperative but challenging because de-domestication can follow different trajectories and proceed cryptically [65]. Some weedy rice are visually indistinguishable from cultivars except for the shattering phenotype because some weedy rice have the same overall appearance and grain size as the cultivar, and have white pericarp, but this is not the case of California or other US weedy rice [66–67]. Also, seed morphology differences between weedy and cultivated rice in the field may only become apparent after hulling when the red caryopsis is visible, effectively concealing the weed during invasion [68]. Emergence, establishment, and spread of weedy rice in places that have no wild *Oryza* provide clues to how feral forms cryptically evolve. For example, re-seeding with previously grown cultivated rice in Malaysia has selected for weedy ecotypes which shatter their seed [69]. In instances where farmers cease cultivating a landrace to take advantage of a new cultivar with better yield, the agroecological conditions are altered to prime the environment for de-domestication to proceed. Abandoned landrace rice can establish, and because farmers are familiar with the similar appearance of volunteer landrace varieties under cultivation, they may not be identified as de-domesticated lineages. This suggests that management strategies must include monitoring at a smaller scale to ensure that escaping individuals with slightly higher variance in weedy traits are immediately identified rather than considered environmental variants of the cultivar.

The historical eradication of weedy rice from California was largely due to intensive management including control of water and use of certified seed. However, unintentional dispersal of weedy individuals via a connectivity corridor such as a river or irrigation system could be responsible for either resurgence or maintenance of weedy rice populations.

### Adaptive evolution of weeds through de-domestication

Evolution of weedy rice by de-domestication is not simply “domestication in reverse,” and involves the interplay of a greater number of mechanistic drivers than rice domestication. Temporal variation in US weedy rice flowering strategies and shared haplotypes with crop ancestors suggest hybridization and evolution on a short timescale [70]. The gene(s) or processes conferring a wild or weedy trait during de-domestication may not be the same ones responsible during domestication [71]. However, because variation is limited to ancestral standing variation and novel mutations, weeds evolving directly from crops consist of fewer de-domesticated haplotypes, making these cases ideal for testing hypotheses about adaptive evolution during de-domestication [39]. Understanding the origin of weedy rice in California will be useful in managing this weed and controlling the evolution of future feral ecotypes. Pinpointing the molecular evolution and genomic factors involved in the weediness associated with this endoferal origin of weedy rice in California is currently underway (Kanapeckas *et al*., *in prep)*.

In the absence of directed husbandry, domesticated lines will maintain or acquire weedy or wild traits to ensure success in resource capture, survival, and fecundity [71–73]. This de-domestication, or evolutionary reversion to wild-type morphology (known also as ferality), can happen through processes involving hybridization with endemic or introduced relatives and involve either natural selection acting on standing genetic variation in the domesticated populations or gene flow from wild relatives [73]. Modes of weed evolution include a domesticate origin with genetic contributions from weedy or wild relatives (exoferality), or solely domesticate-derived forms (endoferality and exo-endoferality).

Evolution of weeds from crop domesticated lines requires changes at several important traits. Seed dispersal (by shattering) is pivotal to the establishment and maintenance of grasses, where the level of shattering is directly related to a weed’s fitness. Because non-shattering is selected for in domesticated grasses, weeds evolving from crops would have to revert to shattering either through gene flow or by re-evolving the trait, assuming there is no standing variation for this trait in domesticated populations. Extended seed dormancy could also increase fitness of a weed, but would be selected against in a crop because cultivation would be difficult. Therefore, the reversion to seed dormancy would be selected for in weedy species. In addition, it is possible that pigmented pericarp may be selected for in weedy rice evolution due to its association with the ability of seeds to remain dormant for long periods of time [74].

Weedy rice emerging in cultivated rice populations could be explained by the quick loss of a few domestication alleles in regions of the genome that experienced relaxation of selective pressure. Based on an evolutionary dynamic described in [75], the evolutionary genetic dynamics we observe here could be those of deep divergence of non-endemic wild species at neutral loci (STS) but allele-sharing at key wild-like loci, which necessitates a closer look at candidate genes. An interesting further avenue of research will be to sequence known genetic regions that confer weedy characteristics and whose patterns have been identified in both weedy and cultivated rice to have a better understanding of the evolution of weediness as manifested in the California ecotype (currently underway—Kanapeckas *et al*. in prep.). The *Rc* locus would be a good candidate because most rice cultivated in California would carry the loss of function mutation, resulting in a white pericarp. We can determine if California weedy rice shares polymorphisms associated with the loss of function allele (*rc*), but has re-evolved pigmented pericarp either by reversion or by changes at another locus. We could also test if California weedy rice has captured the ancestral functional allele through gene flow from another source such as cultivated rice with a red pigmented pericarp or wild or weedy relatives present outside of the US.

In closing, we present compelling evidence of rapid independent origin of weedy races of rice from cultivated relatives and contribute to our understanding of adaptive evolution under domestication. The use of divergence population genetics to track crop and weed interactions, as done in this study, is useful in understanding how weeds evolve and what approaches can be used to best control their spread. However, there is still much to learn about the extent to which contemporary populations diverge in genetic and morpho-physiological background from their non-wild progenitors during de-domestication.

## Supporting Information

S1 File **Figure A. Dendrogram from UPGMA hierarchical cluster analysis of morphological traits in California cultivated (medium-grain and gourmet-boutique) and weedy rice**. The analysis was performed in SAS^®^ version 9.3 (Cary, NC, USA). **Table A**. Accessions, phenotypic traits, and agroecological variables (with life history stages) used in this study. **Table B**. Eigenvector values for initial PCA with highly correlated and variables with no variation excluded. **Table C.** California weedy rice (CWR) and California cultivated rice morphogroups (CCR1: medium-grain cultivars) and CCR2: gourmet cultivars) determined by UPGMA cluster analysis (Figure A in S1 File). Weedy rice morphotype code based on the measured traits listed in Table A in S1 File. Numbers in parenthesis for qualitative traits correspond to IRRI (2012) morphological rice descriptors (category). Average values for quantitative measurements are mean (± SD) of traits per morphotype. Data indicated “mother plant” are from field-collected plants, while offspring from the field-collected seeds are majority of traits. Phenotype collection information for traits without Category descriptors can also be found in the International Rice Research Institute’s Standard Evaluation System for Rice (IRRI, 2012; available http://bit.ly/1l7TIt0.]. **Table D**. Standard sequence diversity indices (e.g. segregating sites, sequence diversity pi, Watterson’s theta, polymorphic loci, numbers of mutations, synonymous versus nonsynonymous replacements) for sequence tagged site (STS) loci in all groups of *Oryza* analyzed in this study. **Table E.** STRUCTURE mean log likelihood results. Mean log likelihood of each K value (LnP(K)) and variance are shown for all K cluster models evaluated in STRUCTURE. The most likely value of K is shown in bold. **Table F**. Divergence measures for 48 STS loci. Estimates of gene flow (*F*_*ST*_), number of migrants (*Nm*), and number of net nucleotide substitutions per site between populations (*Da*). *N* is the number of individuals. *No polymorphic sites in the region. CWR indicates California weedy rice (*O*. *sativa*), CCR indicates California cultivated rice, Population 1 (Pop 1) is intraspecific and Population 2 (Pop 2) is interspecific.
(DOC)
