# Supplementary material for: Escape to Ferality: The Endoferal Origin of Weedy Rice from Crop Rice through De-Domestication
Source: PLoS One. 2016 Sep 23;11(9):e0162676. doi: 10.1371/journal.pone.0162676 (PMC5035073; doi:10.1371/journal.pone.0162676)
Supplement: S1 File — Figure A. Dendrogram from UPGMA hierarchical cluster analysis of morphological traits in California cultivated (medium-grain and gourmet-boutique) and weedy rice. The analysis was performed in SAS® version 9.3 (Cary, NC, USA). Table A. Accessions, phenotypic traits, and agroecological variables (with life history stages) used in this study. Table B. Eigenvector values for initial PCA with highly correlated and variables with no variation excluded. Table C. California weedy rice (CWR) and California cultivated rice morphogroups (CCR1: medium-grain cultivars) and CCR2: gourmet cultivars) determined by UPGMA cluster analysis (Figure A in S1 File). Weedy rice morphotype code based on the measured traits listed in Table A in S1 File. Numbers in parenthesis for qualitative traits correspond to IRRI (2012) morphological rice descriptors (category). Average values for quantitative measurements are mean (± SD) of traits per morphotype. Data indicated “mother plant” are from field-collected plants, while offspring from the field-collected seeds are majority of traits. Phenotype collection information for traits without Category descriptors can also be found in the International Rice Research Institute’s Standard Evaluation System for Rice (IRRI, 2012; available http://bit.ly/1l7TIt0.]. Table D. Standard sequence diversity indices (e.g. segregating sites, sequence diversity pi, Watterson’s theta, polymorphic loci, numbers of mutations, synonymous versus nonsynonymous replacements) for sequence tagged site (STS) loci in all groups of Oryza analyzed in this study. Table E. STRUCTURE mean log likelihood results. Mean log likelihood of each K value (LnP(K)) and variance are shown for all K cluster models evaluated in STRUCTURE. The most likely value of K is shown in bold. Table F. Divergence measures for 48 STS loci. Estimates of gene flow (FST), number of migrants (Nm), and number of net nucleotide substitutions per site between populations (Da). N is the number of individuals. * [file pone.0162676.s001.doc]

FILE S1: FIGURE A AND TABLES A-F

**Fig A.** **Dendrogram from UPGMA hierarchical cluster analysis of morphological traits in California cultivated (medium-grain and gourmet-boutique) and weedy rice**. The analysis was performed in SAS® version 9.3 (Cary, NC, USA).

**
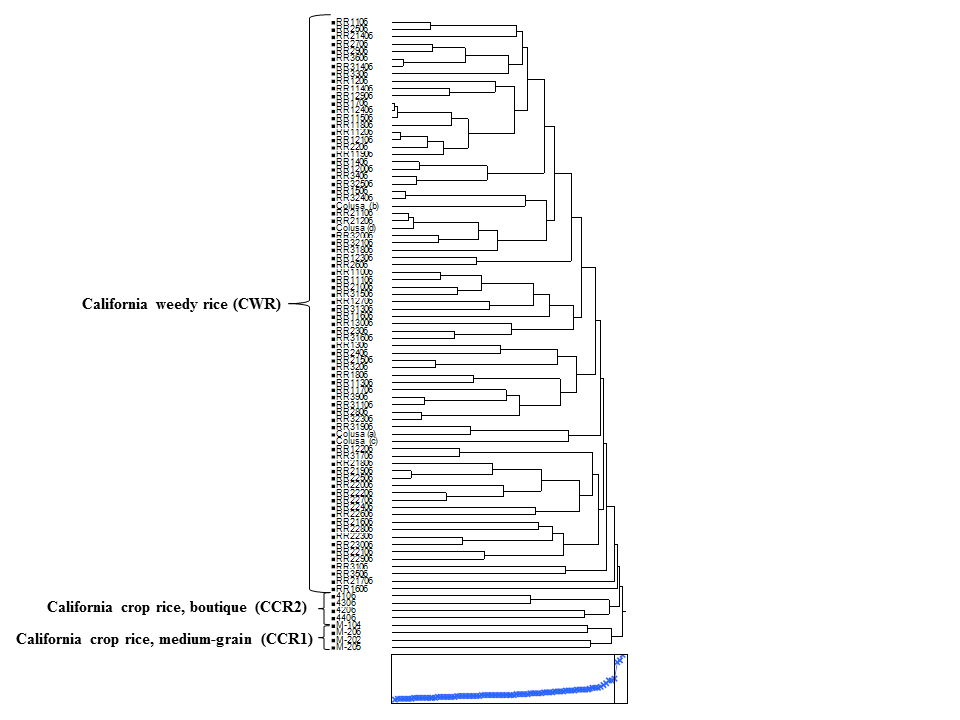
**

| **Accession** | **Vegetative Traits** |
| --- | --- |
| RR1106 | Leaf pubescence |
| RR1206 | Leaf color |
| RR1306 | Leaf texture |
| RR1406 | Leaf sheath color |
| RR1506 | Leaf angle |
| RR1606 | Ligule shape |
| RR1706 | Ligule color |
| RR1806 | Basal leaf sheath color |
| RR11006 | Auricle color |
| RR11106 | Plant height seedling |
| RR11206 | Number of tillers |
| RR11306 | Plant height mature plant |
| RR11406 | **Reproductive Traits** |
| RR11506 | Culm angle |
| RR11606 | Culm length |
| RR11706 | Culm strength (lodging resistance) |
| RR11806 | Internode color |
| RR11906 | Node color |
| RR12006 | Flag leaf angle |
| RR12106 | Panicle type |
| RR12206 | Panicle secondary branching |
| RR12306 | Panicle exsertion |
| RR12406 | Panicle axis |
| RR12706 | Texture of panicle axis |
| RR12906 | Awning |
| RR13006 | Awn color |
| RR2206 | Apiculus color |
| RR2306 | Hull color |
| RR2406 | Days from effective seeding date to 80% heading |
| RR2506 | Culm length |
| RR2606 | Culm diameter |
| RR2706 | Auricle length |
| RR2806 | Ligule length |
| RR2906 | Number of panicle |
| RR21006 | Panicle length |
| RR21106 | Distance panicle base to 1st spikelet insertion |
| RR21206 | Awn length |
| RR21406 | Leaf length |
| RR21506 | Leaf width |
| RR21606 | Flag leaf length |
| RR21706 | Flag leaf width |
| RR21806 | Number of grains per panicle |
| RR21906 | Total grains per panicle |
| RR22006 | Flowering |
| RR22106 | **Harvest/Post-harvest Traits** |
| RR22206 | Pericarp (bran) color |
| RR22306 | Panicle (seed) shattering |
| RR22406 | Color of glumes (mother plant) |
| RR22506 | Lemma pubescence |
| RR22606 | Pericarp (bran) color (mother plant) |
| RR22706 | Spikelet fertility (%) |
| RR22806 | 100-grain weight |
| RR22906 | Length paddy grain |
| RR23006 | Width paddy grain |
| RR3106 | Thickness paddy grain |
| RR3206 | Length/width paddy grain |
| RR3306 | Grain length |
| RR3406 | Grain width |
| RR3506 | Length/width grain |
| RR3606 | Days to 80% maturity |
| RR3906 | Length of awn (mother plant) |
| RR31106 | Grain length (mother plant) |
| RR31306 | Grain width (mother plant) |
| RR31406 | Grain thickness |
| RR31506 | Grain thickness (mother plant) |
| RR31606 | Sterile lemma length |
| RR31706 | 10-grain weight (mother plant) |
| RR31806 | 100-grain weight (mother plant) |
| RR31906 |  |
| RR32006 |  |
| RR32106 |  |
| RR32306 |  |
| RR32406 |  |
| RR32506 |  |
| RRColusa (a) |  |
| RRColusa (b) |  |
| RRColusa (c) |  |
| RRColusa (d) |  |
| **Accessions** |  |
| **California Crop Rice, Medium-grain (CCR1)** |  |
| M-104 |  |
| M-202 |  |
| M-205 |  |
| M-206 |  |
| **California Crop Rice, Boutique vars. (CCR2)** |  |
| 4106, 4206, 4306, 4406 |  |

**Table A**. Accessions, phenotypic traits, and agroecological variables (with life history stages) used in this study.

| **Phenotypic Trait** | **Principal component 1 (20.3%)** | **Principal component 2 (16.5%)** | **Principal component 3 (8.3%)** |
| --- | --- | --- | --- |
|  |  |  |  |
| Panicle type | **0.35120** | 0.07803 | 0.02662 |
| Texture of panicle axis | -0.21054 | **-0.31100** | 0.13489 |
| Awn color | **0.27386** | -0.20338 | 0.17160 |
| Flowering (1=early; 2=intermediate; 3=late) | **0.28295** | 0.07038 | 0.02572 |
| Lemma pubescence | 0.21056 | **0.31099** | -0.13493 |
| Plant height | -0.09136 | 0.15518 | -0.15474 |
| Number of tillers | 0.15427 | 0.02923 | -0.01405 |
| Culm length | **0.24022** | 0.17942 | -0.16508 |
| Culm diameter | -0.04501 | 0.14298 | 0.11290 |
| Ligule length | -0.01814 | 0.09874 | 0.19108 |
| Panicle length | 0.07562 | 0.11291 | -0.22275 |
| Distance from panicle base to 1st spikelet insertion | -0.06890 | 0.03486 | -0.14557 |
| Awn length | 0.23152 | 0.11714 | **0.29737** |
| Leaf length (1st leaf below flag) | 0.11920 | 0.08154 | -0.04448 |
| Leaf width (1st leaf below flag) | 0.05548 | **-0.26918** | 0.15156 |
| Length flag leaf | **0.29832** | 0.09742 | 0.05547 |
| Flag leaf width | 0.18853 | **-0.24824** | 0.15749 |
| Number of grains per panicle | 0.01760 | -0.09679 | **-0.24248** |
| Spikelet fertility (%) | -0.09288 | -0.13528 | -0.12232 |
| 100-grain weight | 0.07195 | -0.16813 | **0.27504** |
| Length paddy grain | -0.15282 | 0.22788 | 0.01112 |
| Width paddy grain | 0.14003 | -0.10333 | -0.01522 |
| Thickness paddy grain | -0.04981 | -0.08016 | 0.22973 |
| Length/width paddy grain | -0.21435 | 0.23020 | 0.00915 |
| Grain length | -0.13482 | 0.17325 | 0.16597 |
| Grain width | 0.08982 | -0.19339 | -0.02000 |
| Grain thickness | -0.07898 | -0.13189 | 0.18732 |
| Length/width grain | -0.15370 | **0.25240** | 0.11202 |
| Days to 80% maturity | 0.22536 | 0.13700 | 0.03202 |
| Length awn mother plant | 0.23008 | 0.12499 | **0.27142** |
| Grain length mother plant | -0.20328 | 0.15470 | 0.22013 |
| Grain width mother plant | -0.05653 | 0.08397 | 0.14124 |
| Grain thickness mother plant | -0.07407 | 0.21781 | 0.17604 |
| Sterile lemma length | 0.03348 | 0.21558 | 0.22145 |
| 100-grain weight mother plant | -0.13558 | 0.05627 | **0.34459** |

**Table B**. Eigenvector values for initial PCA with highly correlated and variables with no variation excluded.

|  | | | **California Rice Morphotypes** | | |
| --- | --- | --- | --- | --- | --- |
|  |  | | **CWR** | **CCR1** | **CCR2** |
| **Trait / Variable** | **Code** | **Category** | **(N=78)** | **(N=4)** | **(N=4)** |
| **Qualitative Traits** | | | | | |
| Leaf pubescence | 1 | Glabrous (smooth) including ciliated margins |  | 100% |  |
|  | 2 | intermediate | 100% |  | 100% |
| Leaf color | 60 | green |  | 100% |  |
|  | 61 | light green | 100% |  | 100% |
| Leaf texture | 1 | herbaceous | 100% | 100 | 100% |
| Leaf sheath color | 60 | green | 100% | 100% |  |
|  | 80 | purple (full) |  |  | 1 (25%) |
|  | 84 | purple lines |  |  | 3 (75%) |
| Leaf angle | 1 | erect | 100% | 100% | 100% |
| Ligule shape | 2 | cleft | 100% | 100% | 100% |
| Ligule color | 11 | whitish | 100% | 100% | 100% |
| Basal leaf sheath color | 61 | light green | 100% | 100% |  |
|  | 80 | purple |  |  | 100% |
| Auricle color | 61 | light green | 100% | 100% | 100% |
| Culm angle | 1 | erect- angle < 30° from perpendicular |  | 100% |  |
|  | 3 | intermediate-angle is about 45° from perpendicular | 100% |  |  |
|  | 7 | Spreading - the angle > 60° but culms do not rest on the ground |  |  | 100% |
| Culm strength | 3 | most plants leaning | 100% | 100% | 100% |
| Internode color | 41 | light gold | 100% |  |  |
|  | 60 | green |  | 100% |  |
|  | 84 | purple lines |  |  | 100% |
| Node color | 60 | green | 100% | 100% | 100% |
| Flag leaf angle | 1 | erect | 100% | 100% |  |
|  | 3 | intermediate |  |  | 100% |
| Panicle type | 1 | compact |  | 100% | 100% |
|  | 3 | open | 100% |  |  |
| Panicle secondary branching | 2 | heavy | 100% | 100% | 100% |
| Panicle exsertion | 1 | well exserted - panicle appears well above the collar of flag leaf blade | 100% | 100% |  |
|  | 5 | just exserted - the panicle base coincides with the collar of the flag leaf |  |  | 100% |
| Panicle axis | 1 | straight at maturity | 100% |  | 100% |

| **Trait / Variable**   | **Code** | **Category** | **CWR** | **CCR1** | **CCR2** |  | | --- | --- | --- | --- | --- | --- | |  |  |  |  |  |  | | | | | | |
| --- | --- | --- | --- | --- | --- | --- | --- | --- | --- | --- | --- | --- | --- | --- | --- | --- | --- |
| **Qualitative Traits** | | | | | |
|  | 2 | droopy at maturity |  | 100% |  |
| Texture of panicle axis | 1 | increasingly hispid-scabrous to tip | 100% |  | 100% |
|  | 2 | not increasingly |  | 100% |  |
| Awning | 0 | absent |  |  | 100% |
|  | 1 | short and partly awned |  | 100% |  |
|  | 9 | long and fully awned | 100% |  |  |
| Awn color | 0 | awnless |  |  | 100% |
|  | 20 | straw | 100% | 100% |  |
| Apiculus color | 10 | white | 100% | 100% |  |
|  | 70 | red |  |  | 100% |
| Hull color | 20 | straw | 100% | 100% |  |
|  | 52 | brown (tawny) |  |  | 100% |
| Pericarp color | 10 | white |  | 100% |  |
|  | 80 | purple | 100% |  | 100% |
| Stigma color | 10 | white | 100% | 100% |  |
|  | 80 | purple |  |  | 100% |
| Ligule pubescence | 2 | hirsute in specific places | 100% | 100% | 100% |
| Seed (panicle) shattering | 1 | very low (<1%) |  | 100% |  |
|  | 5 | moderate (6-25%) | 5% |  | 100% |
|  | 9 | more than 50% | 95% |  |  |
| Flowering | 1 | early |  | 50% | 50% |
|  | 2 | intermediate | 36% | 50% | 25% |
|  | 3 | late | 64% |  | 25% |
| Color of glumes  (mother plant) | 20 | straw | 100% | 100% |  |
|  | 52 | brown (tawny) |  |  | 100% |
| Lemma pubescence | 1 | glabrous |  | 100% |  |
|  | 4 | short hairs | 97% |  | 100% |
|  | 5 | long hairs (velvety) | 3% |  |  |
| Pericarp (bran) color (mother plant) | 10 | white |  | 100% |  |
|  | 50 | brown | 28% |  |  |
|  | 80 | purple | 72% |  | 100% |

| **Quantitative Traits** | **CWR** | **CCR1** | **CCR2** |
| --- | --- | --- | --- |
| Plant height (seedling) | 15.4 (± 1.1) | 14.4 (±0.67) | 18.0 (±1.7) |
| Number of days from effective seeding to 80% heading | 110 (±1.9) | 1031 (±3.6) | 106 (±4.5) |
| Number of tillers | 19.4 (±3.0) | 13.4 (±2.0) | 15.1(±2.2) |
| Plant height (mature) | 110 (±6.7) | 76.8 (±3.5) | 104 (±2.5) |
| Culm length (cm) | 89.2 (±5.2) | 61.1 (±3.1) | 82.7 (±2.2) |
| Culm diameter (mm) | 2.98 (±0.39) | 2.72 (±0.21) | 3.44 (±0.29) |
| Ligule or auricle length (mm) | 15.4 (±2.0) | 14.6 (±1.2) | 16.3 (±1.1) |
| Number of panicles | 17.6 (±2.6) | 12.8 (±1.7) | 14.7 (±2.7) |
| Panicle length (cm) | 20.7 (±3.3) | 15.4 (±1.1) | 21.9 (±0.25) |
| Distance from panicle base to 1st spikelet insertion (mm) | 4.92 (±3.4) | 4.5 (±1.7) | 8.20 (±3.3) |
| Awn length (mm) | 43.5 (±13.8) | 6.35 (±3.1) | 0 |
| Leaf length (1st leaf below flag) (cm) | 45.8 (±12.5) | 28.5 (±3.0) | 35.4 (±1.8) |
| Leaf width (1st leaf below flag) (cm) | 9.74 (±0.77) | 11.9 (±1.063) | 7.78 (±0.34) |
| Flag leaf length | 33.7 (±3.4) | 18.6 (±1.9) | 21.04 (±1.4) |
| Flag leaf width | 12.7 (±0.74) | 14.01 (±0.97) | 8.76 (±0.64) |
| Number of grains per panicle | 70.4 (±11.6) | 74.6 (±16.2) | 64.0 (±8.3) |
| Total grains per panicle | 22 (±7.0) | 10.3 (±1.5) | 17.6 (±7.08) |
| Spikelet fertility (%) | 76.1 (±7.07) | 87.72 (±1.8) | 78.03 (±9.1) |
| 100-grain weight (g) | 2.53 (±0.089) | 2.67 (±0.10) | 2.33 (±0.13) |
| Paddy grain length (mm) | 8.27 (±0.28) | 8.09 (0.27) | 9.17 (±0.20) |
| Paddy grain width (mm) | 3.15 (±0.15) | 3.11 (±0.16) | 2.94 (±0.19) |
| Paddy grain thickness (mm) | 1.96 (±0.093) | 2.07 (±0.15) | 1.92 (±0.057) |
| Paddy grain length/width ratio | 2.63 (±0.13) | 2.61 (±0.11) | 3.13 (±0.19) |
| Grain length (mm) | 6.4 (±0.22) | 6.40 (±0.12) | 6.8 (±0.21) |
| Grain width (mm) | 2.67 (±0.11) | 2.77 (±0.076) | 2.50 (±0.085) |
| Grain thickness (mm) | 1.76 (±0.082) | 1.92 (±0.034) | 1.71 (±0.10) |
| Grain L/W ratio | 2.40 (±0.13) | 2.32 (±0.032) | 2.72 (±0.10) |
| Days to 80% maturity | 150 (±1.91) | 143 (±3.78) | 146 (±4.54) |
| Awn length mother plant (mm) | 44.4 (±15.4) | 0 | 0 |
| Grain length mother plant (mm) | 8.031 (±0.34) | 8.3 (±0.34) | 8.93 (±0.18) |
| Grain width mother plant (mm) | 3.19 (±0.15) | 3.12 (±0.081) | 3.29 (±0.023) |
| Grain thickness mother plant (mm) | 2.069 (±0.075) | 2.0 (±0.036) | 2.17 (±0.035) |
| Sterile lemma length (mm) | 3.65 (±0.36) | 3.083 (±0.29) | 3.81 (±0.23) |
| 10-grain weight mother plant (g) | 0.27 (±0.029) | 0.30 (±0.013) | 0.29 (±0.008) |
| 100-grain weight mother plant (g) | 2.69 (±0.29) | 3.02 (±0.13) | 2.91 (±0.78) |

**Table C.** California weedy rice (CWR) and California cultivated rice morphogroups (CCR1: medium-grain cultivars) and CCR2: gourmet cultivars) determined by UPGMA cluster analysis. Weedy rice morphotype code based on the measured traits listed in Table A of S1 File. Numbers in parenthesis for qualitative traits correspond to IRRI (2012) morphological rice descriptors (category). Average values for quantitative measurements are mean (± SD) of traits per morphotype. Data indicated “mother plant” are from field-collected plants, while offspring from the field-collected seeds are majority of traits. Phenotype collection information for traits without Category descriptors can also be found in the International Rice Research Institute’s Standard Evaluation System for Rice (IRRI, 2012; available <http://bit.ly/1l7TIt0>.

| **Genetic diversity summary statistics for all 48 STS loci (summary of all plus each individual locus) - Kanapeckas et al., 2016** | | | | | | | | | | |  | |  |
| --- | --- | --- | --- | --- | --- | --- | --- | --- | --- | --- | --- | --- | --- |
|  |  |  |  |  |  |  |  |  |  |  | |  |  |
| ***Summary all STS loci*** | | |  |  |  |  |  |  |  |  | |  |  |
|  |  | Silent sites: | |  |  |  | Coding region only: | |  |  | |  |  |
|  |  | **# Seg sites (total)** | **Average Pi JC/kb** | **Median Pi JC/Kb** | **Average theta/Kb** | **Median Theta/kB** | **# Mutations** | **#synonmous** | **#replacement** | |  | |  |
|  | CAred | 6 | 0.02479 | 0.00000 | 0.07500 | 0.00000 | 2 | 2 | 0 |  | |  |  |
|  | TempJap | 62 | 0.73792 | 0.00000 | 1.04313 | 0.00000 | 18 | 9 | 9 |  | |  |  |
|  | TropJap | 102 | 1.51063 | 0.27000 | 1.56708 | 0.65500 | 37 | 23 | 14 |  | |  |  |
|  | Aromatic | 78 | 2.17208 | 0.00000 | 1.88979 | 0.00000 | 37 | 31 | 6 |  | |  |  |
|  | CAcult | 36 | 0.76000 | 0.00000 | 0.65521 | 0.00000 | 14 | 8 | 6 |  | |  |  |
|  | ARcult | 112 | 2.76833 | 0.66500 | 2.20896 | 1.00500 | 34 | 19 | 15 |  | |  |  |
|  | Aus | 52 | 1.62000 | 0.33000 | 1.20563 | 0.36500 | 13 | 5 | 8 |  | |  |  |
|  | indica | 110 | 2.15271 | 0.73500 | 1.79771 | 0.87500 | 42 | 25 | 17 |  | |  |  |
|  | SHWeedy | 32 | 0.84229 | 0.00000 | 0.59938 | 0.00000 | 14 | 8 | 6 |  | |  |  |
|  | MixWeedy | 96 | 3.16125 | 1.60500 | 2.43896 | 1.25500 | 37 | 23 | 14 |  | |  |  |
|  | BrHWeedy | 11 | 0.41292 | 0.00000 | 0.38521 | 0.00000 | 2 | 0 | 2 |  | |  |  |
|  | BHWeedy | 64 | 1.15771 | 0.00000 | 1.17021 | 0.00000 | 12 | 8 | 4 |  | |  |  |
|  | RufiChina | 221 | 4.58063 | 2.48500 | 4.84250 | 2.98000 | 94 | 59 | 35 |  | |  |  |
|  | RufiIndia | 315 | 6.42604 | 3.31000 | 6.22396 | 3.85000 | 129 | 87 | 42 |  | |  |  |
|  | RufiSEAsia | 432 | 7.27396 | 5.11000 | 7.43771 | 6.21000 | 169 | 102 | 67 |  | |  |  |
|  | Nivara | 122 | 4.92625 | 1.82500 | 4.09271 | 1.50000 | 46 | 30 | 16 |  | |  |  |
|  | Glumae | 32 | 1.40896 | 0.00000 | 1.20104 | 0.00000 | 16 | 8 | 8 |  | |  |  |
|  | Barthii | 63 | 2.05583 | 0.00000 | 1.67917 | 0.00000 | 22 | 8 | 14 |  | |  |  |
|  | glaber | 39 | 1.28542 | 0.00000 | 1.08000 | 0.00000 | 17 | 9 | 8 |  | |  |  |
|  | Meridionalis | 48 | 2.69771 | 0.00000 | 2.16271 | 0.00000 | 35 | 22 | 13 |  | |  |  |
| ***Individual loci*** | |  |  |  |  |  |  |  |  |  | |  |  |
| **STS004** | SC | 8 | 0.01669 | 0.01688 | 0.0137 | 1.2971 | 4 | 4 | 0 |  | |  |  |
|  | ARred | 15 | 0.01758 | 0.01779 | 0.01092 | 1.59858 | 4 | 4 | 0 |  | |  |  |
|  | CAred | 0 | 0 | 0 | 0 | na | 0 | 0 | 0 |  | |  |  |
|  |  | 6 | 0.01113 | 0.01121 | 0.00975 | 0.80504 | x | x | x |  | |  |  |
|  | Tempjap | 0 | 0 | 0 | 0 | na | 0 | 0 | 0 |  | |  |  |
|  |  | 12 | 0.00693 | 0.00697 | 0.01141 | -1.33607 | x | x | x |  | |  |  |
|  |  | 0 | 0 | 0 | 0 | na | x | x | x |  | |  |  |
|  | CAcult | 0 | 0 | 0 | 0 | na | 0 | 0 | 0 |  | |  |  |
|  | ARcult | 3 | 0.00354 | 0.00355 | 0.00327 | 0.20727 | 1 | 1 | 0 |  | |  |  |
|  |  | 4 | 0.00838 | 0.00842 | 0.00555 | 2.155 | x | x | x |  | |  |  |
|  | indica | 11 | 0.01555 | 0.01571 | 0.01019 | 1.7316 | 3 | 3 | 0 |  | |  |  |
|  | SHWeedy | 11 | 0.01902 | 0.01926 | 0.00997 | 2.91745 | 3 | 3 | 0 |  | |  |  |
|  | MixWeedy | 9 | 0.01892 | 0.01917 | 0.01254 | 1.95339 | 4 | 4 | 0 |  | |  |  |
|  | BrHWeedy | 0 | 0 | 0 | 0 | na | 0 | 0 | 0 |  | |  |  |
|  | BHWeedy | 10 | 0.01794 | 0.01815 | 0.00888 | 3.20548 | 4 | 4 | 0 |  | |  |  |
|  | Rufi | 29 | 0.02313 | 0.0235 | 0.021 | 0.13604 |  |  |  |  | |  |  |
|  |  | 5 | 0.01304 | 0.01315 | 0.01067 | 2.12492 | x | x | x |  | |  |  |
|  | Glumae | 0 | 0 | 0 | 0 | na | 0 | 0 | 0 |  | |  |  |
|  |  | 2 | 0.00522 | 0.00523 | 0.00427 | 1.89306 | x | x | x |  | |  |  |
|  | glaber | 9 | 0.01521 | 0.01536 | 0.01368 | 0.55061 | 2 | 2 | 0 |  | |  |  |
|  |  | 11 | 0.00732 | 0.00736 | 0.01163 | -1.21837 | x | x | x |  | |  |  |
|  |  |  |  |  |  |  |  |  |  |  | |  |  |
|  | Updated Seq Set Analyses | | |  |  |  |  |  |  |  | |  |  |
|  |  |  |  |  |  |  |  |  |  |  | |  |  |
|  |  | # seg sites | pi silent | pi (JC) | theta silent | Taj D |  |  |  |  | |  |  |
|  | CAredWell | 0 | 0 | 0 | 0 | na | 0 | 0 | 0 |  | |  |  |
|  | DemmerWest1 | 0 | 0 | 0 | 0 | na | 0 | 0 | 0 |  | |  |  |
|  | DemmerWest2 | 0 | 0 | 0 | 0 | na | 0 | 0 | 0 |  | |  |  |
|  | DemmerEast | 0 | 0 | 0 | 0 | na | 0 | 0 | 0 |  | |  |  |
|  | TropJap | 12 | 0.00429 | 0.0043 | 0.01014 | -1.75045 | 4 | 4 | 0 |  | |  |  |
|  | Aromatic | 0 | 0 | 0 | 0 | na | 0 | 0 | 0 |  | |  |  |
|  | Aus | 4 | 0.00857 | 0.00862 | 0.0052 | 2.53271 | 1 | 1 | 0 |  | |  |  |
|  | RufiChina | 21 | 0.0297 | 0.03031 | 0.02454 | 0.6706 | 5 | 5 | 0 |  | |  |  |
|  | RufiIndia | 16 | 0.01896 | 0.0192 | 0.01618 | 0.71023 | 3 | 3 | 0 |  | |  |  |
|  | RufiSEAsia | 27 | 0.02832 | 0.02887 | 0.02404 | 0.46547 | 7 | 5 | 2 |  | |  |  |
|  | Nivara | 8 | 0.01675 | 0.01694 | 0.01376 | 1.31709 | 2 | 2 | 0 |  | |  |  |
|  | Barthii | 2 | 0.00417 | 0.00418 | 0.00343 | 1.03194 | 2 | 2 | 0 |  | |  |  |
|  | Meridionalis | 0 | 0 | 0 | 0 | na | 0 | 0 | 0 |  | |  |  |
|  |  |  |  |  |  |  |  |  |  |  | |  |  |
| **STS005** |  | # seg sites | pi silent | pi (JC) | theta silent | Taj D | polymor. loci | synonymous | replacement | |  | |  |
|  | SC | 0 | 0 | 0 | 0 | na | 0 | 0 | 0 |  | |  |  |
|  | ARred | 1 | 0 | 0 | 0 | 0.37283 | 1 | 0 | 1 |  | |  |  |
|  | CAred | 0 | 0 | 0 | 0 | na | 0 | 0 | 0 |  | |  |  |
|  |  | 2 | 0.00591 | 0.00593 | 0.00588 | 0.01889 | x | x | x |  | |  |  |
|  | Tempjap | 0 | 0 | 0 | 0 | na | 0 | 0 | 0 |  | |  |  |
|  |  | 1 | 0 | 0 | 0 | 0.69854 | x | x | x |  | |  |  |
|  |  | 1 | 0 | 0 | 0 | 1.06589 | x | x | x |  | |  |  |
|  | CAcult | 0 | 0 | 0 | 0 | na | 0 | 0 | 0 |  | |  |  |
|  | ARcult | 1 | 0 | 0 | 0 | -0.68111 | 1 | 0 | 1 |  | |  |  |
|  |  | 1 | 0 | 0 | 0 | 0.01499 | x | x | x |  | |  |  |
|  | indica | 0 | 0 | 0 | 0 | na | 0 | 0 | 0 |  | |  |  |
|  | SHWeedy | 1 | 0 | 0 | 0 | -0.86644 | 1 | 0 | 1 |  | |  |  |
|  | MixWeedy | 1 | 0 | 0 | 0 | 0.01499 | 1 | 0 | 1 |  | |  |  |
|  | BrHWeedy | 0 | 0 | 0 | 0 | na | 0 | 0 | 0 |  | |  |  |
|  | BHWeedy | 0 | 0 | 0 | 0 | na | 0 | 0 | 0 |  | |  |  |
|  | Rufi | 18 | 0.00983 | 0.00989 | 0.01849 | -1.67279 |  |  |  |  | |  |  |
|  |  | 0 | 0 | 0 | 0 | na | x | x | x |  | |  |  |
|  | Glumae | 1 | 0 | 0 | 0 | 1.63299 | 1 | 0 | 1 |  | |  |  |
|  |  | 1 | 0.00806 | 0.00811 | 0.0066 | 1.63299 | x | x | x |  | |  |  |
|  | glaber | 0 | 0 | 0 | 0 | na | 0 | 0 | 0 |  | |  |  |
|  |  | 1 | 0 | 0 | 0 | -0.08237 | x | x | x |  | |  |  |
|  | PA_rice | 0 | 0 | 0 | 0 |  | 0 | 0 | 0 |  | |  |  |
|  |  |  |  |  |  |  |  |  |  |  | |  |  |
|  | Updated Seq Set Analyses | | |  |  |  |  |  |  |  | |  |  |
|  |  |  |  |  |  |  |  |  |  |  | |  |  |
|  |  | # seg sites | pi silent | pi (JC) | theta silent | Taj D |  |  |  |  | |  |  |
|  | CAredWell | 0 | 0 | 0 | 0 | na | 0 | 0 | 0 |  | |  |  |
|  | DemmerWest1 | 0 | 0 | 0 | 0 | na | 0 | 0 | 0 |  | |  |  |
|  | DemmerWest2 | 0 | 0 | 0 | 0 | na | 0 | 0 | 0 |  | |  |  |
|  | DemmerEast | 0 | 0 | 0 | 0 | na | 0 | 0 | 0 |  | |  |  |
|  | TropJap | 1 | 0 | 0 | 0 | 0.12319 | 1 | 0 | 1 |  | |  |  |
|  | Aromatic | 1 | 0 | 0 | 0 | 1.06589 | 1 | 0 | 1 |  | |  |  |
|  | Aus | 1 | 0 | 0 | 0 | 1.06589 | 1 | 0 | 1 |  | |  |  |
|  | RufiChina | 4 | 0.00628 | 0.00631 | 0.00996 | -0.31546 | 4 | 3 | 1 |  | |  |  |
|  | RufiIndia | 5 | 0.00805 | 0.00809 | 0.00901 | -0.30424 | 5 | 3 | 2 |  | |  |  |
|  | RufiSEAsia | 6 | 0.00947 | 0.00953 | 0.00844 | -0.72331 | 7 | 3 | 4 |  | |  |  |
|  | Nivara | 0 | 0 | 0 | 0 | na | 0 | 0 | 0 |  | |  |  |
|  | Barthii | 1 | 0.00645 | 0.00648 | 0.0053 | 0.85057 | 1 | 1 | 0 |  | |  |  |
|  | Meridionalis | 2 | 0.00807 | 0.00811 | 0.0066 | 1.89306 | 2 | 1 | 1 |  | |  |  |
|  |  |  |  |  |  |  |  |  |  |  | |  |  |
| **STS007** |  | # seg sites | pi silent | pi (JC) | theta silent | Taj D | polymor. loci | synonymous | replacement | |  | |  |
|  | SC | 0 | 0 | 0 | 0 | na | 0 | 0 | 0 |  | |  |  |
|  | ARred | 1 | 0 | 0 | 0 | -0.89904 | 0 | 0 | 0 |  | |  |  |
|  | CAred | 0 | 0 | 0 | 0 | na | 0 | 0 | 0 |  | |  |  |
|  |  |  |  |  |  |  | x | x | x |  | |  |  |
|  | Tempjap | 0 | 0 | 0 | 0 | na | 0 | 0 | 0 |  | |  |  |
|  |  | 1 | 0 | 0 | 0 | -0.81338 | x | x | x |  | |  |  |
|  |  | 0 | 0 | 0 | 0 | na | x | x | x |  | |  |  |
|  | CAcult | 1 | 0 | 0 | 0 | -0.64112 | 1 | 0 | 1 |  | |  |  |
|  | ARcult | 1 | 0 | 0 | 0 | 0.13869 | 1 | 0 | 1 |  | |  |  |
|  |  | 0 | 0 | 0 | 0 | na | x | x | x |  | |  |  |
|  | indica | 0 | 0 | 0 | 0 | na | 0 | 0 | 0 |  | |  |  |
|  | SHWeedy | 0 | 0 | 0 | 0 | na | 0 | 0 | 0 |  | |  |  |
|  | MixWeedy | 1 | 0 | 0 | 0 | 1.30268 | 1 | 0 | 1 |  | |  |  |
|  | BrHWeedy | 0 | 0 | 0 | 0 | na | 0 | 0 | 0 |  | |  |  |
|  | BHWeedy | 0 | 0 | 0 | 0 | na | 0 | 0 | 0 |  | |  |  |
|  | Rufi | 11 | 0.01157 | 0.01166 | 0.01307 | -0.23606 |  |  |  |  | |  |  |
|  |  | 1 | 0 | 0 | 0 | 1.63299 | x | x | x |  | |  |  |
|  | Glumae | 0 | 0 | 0 | 0 | na | 0 | 0 | 0 |  | |  |  |
|  |  | 3 | 0.00717 | 0.0072 | 0.00586 | 2.01187 | x | x | x |  | |  |  |
|  | glaber | 2 | 0 | 0 | 0 | 0.41421 | 2 | 0 | 2 |  | |  |  |
|  |  |  |  |  |  |  | x | x | x |  | |  |  |
|  | PA_rice | 0 | 0 | 0 | 0 |  | 0 | 0 | 0 |  | |  |  |
|  |  |  |  |  |  |  |  |  |  |  | |  |  |
|  | Updated Seq Set Analyses | | |  |  |  |  |  |  |  | |  |  |
|  |  |  |  |  |  |  |  |  |  |  | |  |  |
|  |  | # seg sites | pi silent | pi (JC) | theta silent | Taj D |  |  |  |  | |  |  |
|  | CAredWell | 0 | 0 | 0 | 0 | na | 0 | 0 | 0 |  | |  |  |
|  | DemmerWest1 | 0 | 0 | 0 | 0 | na | 0 | 0 | 0 |  | |  |  |
|  | DemmerWest2 | 0 | 0 | 0 | 0 | na | 0 | 0 | 0 |  | |  |  |
|  | DemmerEast | 0 | 0 | 0 | 0 | na | 0 | 0 | 0 |  | |  |  |
|  | TropJap | 1 | 0 | 0 | 0 | -0.8912 | 1 | 0 | 1 |  | |  |  |
|  | Aromatic | 0 | 0 | 0 | 0 | na | 0 | 0 | 0 |  | |  |  |
|  | Aus | 0 | 0 | 0 | 0 | na | 0 | 0 | 0 |  | |  |  |
|  | RufiChina | 8 | 0.00559 | 0.00561 | 0.0118 | -1.91776 | 9 | 5 | 4 |  | |  |  |
|  | RufiIndia | 10 | 0.01762 | 0.01783 | 0.01335 | 1.10875 | 11 | 6 | 5 |  | |  |  |
|  | RufiSEAsia | 5 | 0.01235 | 0.01245 | 0.00792 | 1.77222 | 5 | 3 | 2 |  | |  |  |
|  | Nivara | 1 | 0 | 0 | 0 | 0.85057 | 1 | 0 | 1 |  | |  |  |
|  | Barthii | 3 | 0.00573 | 0.00576 | 0.00471 | 1.12414 | 3 | 1 | 2 |  | |  |  |
|  | Meridionalis | 1 | 0.00717 | 0.0072 | 0.00587 | 1.63299 | 1 | 1 | 0 |  | |  |  |
|  |  |  |  |  |  |  |  |  |  |  | |  |  |
| **STS011** |  | # seg sites | pi silent | pi (JC) | theta silent | Taj D | polymor. loci | synonymous | replacement | unique alleles | | |  |
|  | SC | 0 | 0 | 0 | 0 | na | 0 | 0 | 0 |  | |  |  |
|  | ARred | 1 | 0.00022 | 0.00022 | 0.00058 | -0.64004 | 0 | 0 | 0 |  | |  |  |
|  | CAred | 0 | 0 | 0 | 0 | na | 0 | 0 | 0 |  | |  |  |
|  |  |  |  |  |  |  | x | x | x |  | |  |  |
|  | Tempjap | 1 | 0.00031 | 0.00031 | 0.00077 | -0.84519 | 1 | 1 | 0 |  | |  |  |
|  |  | 0 | 0 | 0 | 0 | na | x | x | x |  | |  |  |
|  |  | 0 | 0 | 0 | 0 | na | x | x | x |  | |  |  |
|  | CAcult | 0 | 0 | 0 | 0 | na | 0 | 0 | 0 |  | |  |  |
|  | ARcult | 0 | 0 | 0 | 0 | na | 0 | 0 | 0 |  | |  |  |
|  |  | 1 | 0.001 | 0.001 | 0.0011 | -0.19492 | x | x | x |  | |  |  |
|  | indica | 1 | 0.00031 | 0.00031 | 0.00077 | -0.84519 | 1 | 1 | 0 |  | |  |  |
|  | SHWeedy | 0 | 0 | 0 | 0 | na | 0 | 0 | 0 |  | |  |  |
|  | MixWeedy | 1 | 0.00176 | 0.00177 | 0.00117 | 1.30268 | 0 | 0 | 0 |  | |  |  |
|  | BrHWeedy | 0 | 0 | 0 | 0 | na | 0 | 0 | 0 |  | |  |  |
|  | BHWeedy | 0 | 0 | 0 | 0 | na | 0 | 0 | 0 |  | |  |  |
|  | Rufi | 9 | 0.00198 | 0.00198 | 0.00569 | -1.61405 |  |  |  |  | |  |  |
|  |  | 1 | 0.00221 | 0.00221 | 0.0018 | 1.63299 | x | x | x |  | |  |  |
|  | Glumae | 1 | 0 | 0 | 0 | 1.63299 | 1 | 0 | 1 |  | |  |  |
|  |  | 1 | 0.00221 | 0.00221 | 0.0018 | 1.63299 | x | x | x |  | |  |  |
|  | glaber | 0 | 0 | 0 | 0 | na | 0 | 0 | 0 |  | |  |  |
|  |  |  |  |  |  |  | x | x | x |  | |  |  |
|  | PA_rice | 0 | 0 | 0 | 0 |  | 0 | 0 | 0 |  | |  |  |
|  |  |  |  |  |  |  |  |  |  |  | |  |  |
|  | Updated Seq Set Analyses | | |  |  |  |  |  |  |  | |  |  |
|  |  |  |  |  |  |  |  |  |  |  | |  |  |
|  |  | # seg sites | pi silent | pi (JC) | theta silent | Taj D |  |  |  |  | |  |  |
|  | CAredWell | 0 | 0 | 0 | 0 | na | 0 | 0 | 0 |  | |  |  |
|  | DemmerWest1 | 0 | 0 | 0 | 0 | na | 0 | 0 | 0 |  | |  |  |
|  | DemmerWest2 | 0 | 0 | 0 | 0 | na | 0 | 0 | 0 |  | |  |  |
|  | DemmerEast | 0 | 0 | 0 | 0 | na | 0 | 0 | 0 |  | |  |  |
|  | TropJap | 0 | 0 | 0 | 0 | na | 0 | 0 | 0 |  | |  |  |
|  | Aromatic | 1 | 0.001 | 0.001 | 0.0011 | -0.19492 | 1 | 1 | 0 |  | |  |  |
|  | Aus | 1 | 0.00087 | 0.00087 | 0.00104 | -0.34144 | 1 | 1 | 0 |  | |  |  |
|  | RufiChina | 0 | 0 | 0 | 0 | na | 0 | 0 | 0 |  | |  |  |
|  | RufiIndia | 2 | 0.00115 | 0.00115 | 0.00164 | -0.60631 | 1 | 1 | 0 |  | |  |  |
|  | RufiSEAsia | 4 | 0.00236 | 0.00236 | 0.00307 | -0.54959 | 2 | 2 | 0 |  | |  |  |
|  | Nivara | 1 | 0.00176 | 0.00177 | 0.00145 | 0.85057 | 1 | 1 | 0 |  | |  |  |
|  | Barthii | 1 | 0.00176 | 0.00177 | 0.00145 | 0.85057 | 0 | 0 | 0 |  | |  |  |
|  | Meridionalis | 1 | 0.00221 | 0.00221 | 0.0018 | 1.63299 | 0 | 0 | 0 |  | |  |  |
| **STS012** |  | # seg sites | pi silent | pi (JC) | theta silent | Taj D | polymor. loci | synonymous | replacement | |  | |  |
|  | SC | 0 | 0 | 0 | 0 | na | 0 | 0 | 0 |  | |  |  |
|  | ARred | 0 | 0 | 0 | 0 | na | 0 | 0 | 0 |  | |  |  |
|  | CAred | 0 | 0 | 0 | 0 | na | 0 | 0 | 0 |  | |  |  |
|  |  |  |  |  |  |  | x | x | x |  | |  |  |
|  | Tempjap | 0 | 0 | 0 | 0 | na | 0 | 0 | 0 |  | |  |  |
|  |  | 0 | 0 | 0 | 0 | na | x | x | x |  | |  |  |
|  |  | 0 | 0 | 0 | 0 | na | x | x | x |  | |  |  |
|  | CAcult | 0 | 0 | 0 | 0 | na | 0 | 0 | 0 |  | |  |  |
|  | ARcult | 0 | 0 | 0 | 0 | na | 0 | 0 | 0 |  | |  |  |
|  |  | 0 | 0 | 0 | 0 | na | x | x | x |  | |  |  |
|  | indica | 0 | 0 | 0 | 0 | na | 0 | 0 | 0 |  | |  |  |
|  | SHWeedy | 0 | 0 | 0 | 0 | na | 0 | 0 | 0 |  | |  |  |
|  | MixWeedy | 0 | 0 | 0 | 0 | na | 0 | 0 | 0 |  | |  |  |
|  | BrHWeedy | 0 | 0 | 0 | 0 | na | 0 | 0 | 0 |  | |  |  |
|  | BHWeedy | 0 | 0 | 0 | 0 | na | 0 | 0 | 0 |  | |  |  |
|  | Rufi | 19 | 0.0027 | 0.00271 | 0.01307 | -2.2551 |  |  |  |  | |  |  |
|  |  | 0 | 0 | 0 | 0 | na | x | x | x |  | |  |  |
|  | Glumae | 0 | 0 | 0 | 0 | na | 0 | 0 | 0 |  | |  |  |
|  |  | 0 | 0 | 0 | 0 | na | x | x | x |  | |  |  |
|  | glaber | 0 | 0 | 0 | 0 | na | 0 | 0 | 0 |  | |  |  |
|  |  |  |  |  |  |  | x | x | x |  | |  |  |
|  | PA_rice | 0 | 0 | 0 | 0 |  | 0 | 0 | 0 |  | |  |  |
|  |  |  |  |  |  |  |  |  |  |  | |  |  |
|  | Updated Seq Set Analyses | | |  |  |  |  |  |  |  | |  |  |
|  |  |  |  |  |  |  |  |  |  |  | |  |  |
|  |  | # seg sites | pi silent | pi (JC) | theta silent | Taj D |  |  |  |  | |  |  |
|  | CAredWell | 0 | 0 | 0 | 0 | na | 0 | 0 | 0 |  | |  |  |
|  | DemmerWest1 | 0 | 0 | 0 | 0 | na | 0 | 0 | 0 |  | |  |  |
|  | DemmerWest2 | 0 | 0 | 0 | 0 | na | 0 | 0 | 0 |  | |  |  |
|  | DemmerEast | 0 | 0 | 0 | 0 | na | 0 | 0 | 0 |  | |  |  |
|  | TropJap | 0 | 0 | 0 | 0 | na | 0 | 0 | 0 |  | |  |  |
|  | Aromatic | 0 | 0 | 0 | 0 | na | 0 | 0 | 0 |  | |  |  |
|  | Aus | 0 | 0 | 0 | 0 | na | 0 | 0 | 0 |  | |  |  |
|  | RufiChina | 0 | 0 | 0 | 0 | na | 0 | 0 | 0 |  | |  |  |
|  | RufiIndia | 2 | 0 | 0 | 0 | -1.2671 | 2 | 0 | 2 |  | |  |  |
|  | RufiSEAsia | 5 | 0.00216 | 0.00216 | 0.0026 | -0.85049 | 6 | 2 | 4 |  | |  |  |
|  | Nivara | 0 | 0 | 0 | 0 | na | 0 | 0 | 0 |  | |  |  |
|  | Barthii | 1 | 0.00298 | 0.00299 | 0.00245 | 0.85057 | 0 | 0 | 0 |  | |  |  |
|  | Meridionalis | 1 | 0.00372 | 0.00373 | 0.00305 | 1.63299 | 1 | 1 | 0 |  | |  |  |
| **STS021** |  | # seg sites | pi silent | pi (JC) | theta silent | Taj D | polymor. loci | synonymous | replacement | |  | |  |
|  | SC | 0 | 0 | 0 | 0 | na | 0 | 0 | 0 |  | |  |  |
|  | ARred | 16 | 0.00237 | 0.00238 | 0.00807 | -1.92324 | 11 | 5 | 6 |  | |  |  |
|  | CAred | 0 | 0 | 0 | 0 | na | 0 | 0 | 0 |  | |  |  |
|  |  |  |  |  |  |  | x | x | x |  | |  |  |
|  | Tempjap | 3 | 0 | 0 | 0 | 2.52746 | 3 | 0 | 3 |  | |  |  |
|  |  | 13 | 0.01982 | 0.02009 | 0.01133 | 2.46172 | x | x | x |  | |  |  |
|  |  | 13 | 0.02247 | 0.02281 | 0.01534 | 1.99297 | x | x | x |  | |  |  |
|  | CAcult | 3 | 0 | 0 | 0 | -0.95384 | 3 | 0 | 3 |  | |  |  |
|  | ARcult | 19 | 0.02326 | 0.02363 | 0.01508 | 1.70428 | 13 | 6 | 7 |  | |  |  |
|  |  | 0 | 0 | 0 | 0 | na | x | x | x |  | |  |  |
|  | indica | 17 | 0.00991 | 0.00998 | 0.0134 | -0.54918 | 11 | 5 | 6 |  | |  |  |
|  | SHWeedy | 0 | 0 | 0 | 0 | na | 0 | 0 | 0 |  | |  |  |
|  | MixWeedy | 16 | 0.01648 | 0.01666 | 0.01638 | 0.02734 | 13 | 7 | 6 |  | |  |  |
|  | BrHWeedy | 0 | 0 | 0 | 0 | na | 0 | 0 | 0 |  | |  |  |
|  | BHWeedy | 0 | 0 | 0 | 0 | na | 0 | 0 | 0 |  | |  |  |
|  | Rufi | 18 | 0.01366 | 0.01378 | 0.01444 | -0.71646 |  |  |  |  | |  |  |
|  |  | 0 | 0 | 0 | 0 | na | x | x | x |  | |  |  |
|  | Glumae | 0 | 0 | 0 | 0 | na | 0 | 0 | 0 |  | |  |  |
|  |  | 0 | 0 | 0 | 0 | na | x | x | x |  | |  |  |
|  | glaber | 13 | 0.03089 | 0.03155 | 0.02528 | 1.68037 | 11 | 7 | 4 |  | |  |  |
|  |  |  |  |  |  |  | x | x | x |  | |  |  |
|  | PA_rice | 12 | 0.04223 | 0.04346 | 0.04223 |  | 8 | 5 | 3 |  | |  |  |
|  |  |  |  |  |  |  |  |  |  |  | |  |  |
|  | Updated Seq Set Analyses | | |  |  |  |  |  |  |  | |  |  |
|  |  |  |  |  |  |  |  |  |  |  | |  |  |
|  |  | # seg sites | pi silent | pi (JC) | theta silent | Taj D |  |  |  |  | |  |  |
|  | CAredWell | 0 | 0 | 0 | 0 | na | 0 | 0 | 0 |  | |  |  |
|  | DemmerWest1 | 0 | 0 | 0 | 0 | na | 0 | 0 | 0 |  | |  |  |
|  | DemmerWest2 | 0 | 0 | 0 | 0 | na | 0 | 0 | 0 |  | |  |  |
|  | DemmerEast | 0 | 0 | 0 | 0 | na | 0 | 0 | 0 |  | |  |  |
|  | TropJap | 13 | 0.01346 | 0.01358 | 0.01001 | 1.03052 | 10 | 7 | 3 |  | |  |  |
|  | Aromatic | 13 | 0.02247 | 0.02281 | 0.01534 | 1.99297 | 10 | 7 | 3 |  | |  |  |
|  | Aus | 0 | 0 | 0 | 0 | na | 0 | 0 | 0 |  | |  |  |
|  | RufiChina | 4 | 0.00517 | 0.00519 | 0.00369 | 1.23666 | 4 | 1 | 3 |  | |  |  |
|  | RufiIndia | 17 | 0.0112 | 0.01129 | 0.0184 | -1.1478 | 9 | 5 | 4 |  | |  |  |
|  | RufiSEAsia | 18 | 0.01243 | 0.01253 | 0.01619 | -0.42763 | 12 | 5 | 7 |  | |  |  |
|  | Nivara | 13 | 0.03089 | 0.03155 | 0.02528 | 2.25945 | 10 | 7 | 3 |  | |  |  |
|  | Barthii | 5 | 0.0031 | 0.00311 | 0.00254 | 2.12492 | 5 | 1 | 4 |  | |  |  |
|  | Meridionalis | 5 | 0.00621 | 0.00623 | 0.00508 | 2.12492 | 4 | 1 | 3 |  | |  |  |
| **STS023** |  | # seg sites | pi silent | pi (JC) | theta silent | Taj D | polymor. loci | synonymous | replacement | |  | |  |
|  | SC | 0 | 0 | 0 | 0 | na | 0 | 0 | 0 |  | |  |  |
|  | ARred | 4 | 0.00055 | 0.00055 | 0.00238 | -1.39082 | 3 | 3 | 0 |  | |  |  |
|  | CAred | 0 | 0 | 0 | 0 | na | 0 | 0 | 0 |  | |  |  |
|  |  |  |  |  |  |  | x | x | x |  | |  |  |
|  | Tempjap | 0 | 0 | 0 | 0 | na | 0 | 0 | 0 |  | |  |  |
|  |  | 2 | 0.00158 | 0.00158 | 0.00172 | -0.15405 | x | x | x |  | |  |  |
|  |  | 3 | 0.00317 | 0.00318 | 0.00347 | -0.27845 | x | x | x |  | |  |  |
|  | CAcult | 0 | 0 | 0 | 0 | na | 0 | 0 | 0 |  | |  |  |
|  | ARcult | 5 | 0.00281 | 0.00281 | 0.00472 | -1.17273 | 3 | 3 | 0 |  | |  |  |
|  |  | 0 | 0 | 0 | 0 | na | x | x | x |  | |  |  |
|  | indica | 5 | 0.00236 | 0.00236 | 0.00397 | -1.01999 | 3 | 3 | 0 |  | |  |  |
|  | SHWeedy | 1 | 0.00053 | 0.00053 | 0.00077 | -0.41765 | 1 | 1 | 0 |  | |  |  |
|  | MixWeedy | 3 | 0.00259 | 0.00259 | 0.00363 | -1.03446 | 2 | 2 | 0 |  | |  |  |
|  | BrHWeedy | 0 | 0 | 0 | 0 | na | 0 | 0 | 0 |  | |  |  |
|  | BHWeedy | 0 | 0 | 0 | 0 | na | 0 | 0 | 0 |  | |  |  |
|  | Rufi | 12 | 0.00511 | 0.00512 | 0.00799 | -1.05844 |  |  |  |  | |  |  |
|  |  | 0 | 0 | 0 | 0 | na | x | x | x |  | |  |  |
|  | Glumae | 1 | 0.00228 | 0.00228 | 0.00186 | 1.63299 | 0 | 0 | 0 |  | |  |  |
|  |  | 1 | 0 | 0 | 0 | 1.63299 | x | x | x |  | |  |  |
|  | glaber | 0 | 0 | 0 | 0 | na | 0 | 0 | 0 |  | |  |  |
|  |  |  |  |  |  |  | x | x | x |  | |  |  |
|  | PA_rice | 0 | 0 | 0 | 0 |  | 0 | 0 | 0 |  | |  |  |
|  |  |  |  |  |  |  |  |  |  |  | |  |  |
|  | Updated Seq Set Analyses | | |  |  |  |  |  |  |  | |  |  |
|  |  |  |  |  |  |  |  |  |  |  | |  |  |
|  |  | # seg sites | pi silent | pi (JC) | theta silent | Taj D |  |  |  |  | |  |  |
|  | CAredWell | 0 | 0 | 0 | 0 | na | 0 | 0 | 0 |  | |  |  |
|  | DemmerWest1 | 0 | 0 | 0 | 0 | na | 0 | 0 | 0 |  | |  |  |
|  | DemmerWest2 | 0 | 0 | 0 | 0 | na | 0 | 0 | 0 |  | |  |  |
|  | DemmerEast | 0 | 0 | 0 | 0 | na | 0 | 0 | 0 |  | |  |  |
|  | TropJap | 2 | 0.00122 | 0.00122 | 0.00152 | -0.34055 | 0 | 0 | 0 |  | |  |  |
|  | Aromatic | 3 | 0.00317 | 0.00318 | 0.00347 | -0.27845 | 2 | 2 | 0 |  | |  |  |
|  | Aus | 0 | 0 | 0 | 0 | na | 0 | 0 | 0 |  | |  |  |
|  | RufiChina | 4 | 0.00556 | 0.00558 | 0.00381 | 1.29103 | 2 | 2 | 0 |  | |  |  |
|  | RufiIndia | 4 | 0.0032 | 0.00321 | 0.00345 | -0.18246 | 3 | 3 | 0 |  | |  |  |
|  | RufiSEAsia | 9 | 0.00511 | 0.00513 | 0.00714 | -0.82259 | 5 | 5 | 0 |  | |  |  |
|  | Nivara | 1 | 0.00182 | 0.00182 | 0.0015 | 0.85057 | 1 | 1 | 0 |  | |  |  |
|  | Barthii | 1 | 0 | 0 | 0 | 0.85057 | 1 | 0 | 1 |  | |  |  |
|  | Meridionalis | 1 | 0.00228 | 0.00228 | 0.00186 | 1.63299 | 0 | 0 | 0 |  | |  |  |
| **sts024** |  | # seg sites | pi silent | pi (JC) | theta silent | Taj D | polymor. loci | synonymous | replacement | unique alleles | | |  |
|  | SC | 2 | 0.00277 | 0.00277 | 0.00202 | 1.75324 | 0 | 0 | 0 |  | |  |  |
|  | ARred | 2 | 0.0003 | 0.0003 | 0.0008 | -0.86508 | 0 | 0 | 0 |  | |  |  |
|  | CAred | 0 | 0 | 0 | 0 | na | 0 | 0 | 0 |  | |  |  |
|  |  |  |  |  |  |  | x | x | x |  | |  |  |
|  | Tempjap | 0 | 0 | 0 | 0 | na | 0 | 0 | 0 |  | |  |  |
|  |  | 1 | 0.00048 | 0.00048 | 0.00056 | -0.2105 | x | x | x |  | |  |  |
|  |  | 0 | 0 | 0 | 0 | na | x | x | x |  | |  |  |
|  | CAcult | 0 | 0 | 0 | 0 | na | 0 | 0 | 0 |  | |  |  |
|  | ARcult | 2 | 0.00119 | 0.00119 | 0.00142 | -0.35434 | 0 | 0 | 0 |  | |  |  |
|  |  | 1 | 0.00126 | 0.00126 | 0.00076 | 1.89122 | x | x | x |  | |  |  |
|  | indica | 5 | 0.00263 | 0.00263 | 0.00268 | -0.30475 | 0 | 0 | 0 |  | |  |  |
|  | SHWeedy | 0 | 0 | 0 | 0 | na | 0 | 0 | 0 |  | |  |  |
|  | MixWeedy | 2 | 0.00236 | 0.00236 | 0.00163 | 1.07659 | 0 | 0 | 0 |  | |  |  |
|  | BrHWeedy | 0 | 0 | 0 | 0 | na | 0 | 0 | 0 |  | |  |  |
|  | BHWeedy | 2 | 0.00123 | 0.00124 | 0.00104 | 1.04355 | 0 | 0 | 0 |  | |  |  |
|  | Rufi | 20 | 0.00443 | 0.00444 | 0.0094 | -1.57358 |  |  |  |  | |  |  |
|  |  | 2 | 0.00307 | 0.00308 | 0.00252 | 1.89306 | x | x | x |  | |  |  |
|  | Glumae | 0 | 0 | 0 | 0 | na | 0 | 0 | 0 |  | |  |  |
|  |  | 0 | 0 | 0 | 0 | na | x | x | x |  | |  |  |
|  | glaber | 0 | 0 | 0 | 0 | na | 0 | 0 | 0 |  | |  |  |
|  |  |  |  |  |  |  | x | x | x |  | |  |  |
|  | PA_rice | 1 | 0.00115 | 0.00115 | 0.00126 |  | 0 | 0 | 0 |  | |  |  |
|  |  |  |  |  |  |  |  |  |  |  | |  |  |
|  | Updated Seq Set Analyses | | |  |  |  |  |  |  |  | |  |  |
|  |  |  |  |  |  |  |  |  |  |  | |  |  |
|  |  | # seg sites | pi silent | pi (JC) | theta silent | Taj D |  |  |  |  | |  |  |
|  | CAredWell | 0 | 0 | 0 | 0 | na | 0 | 0 | 0 |  | |  |  |
|  | DemmerWest1 | 0 | 0 | 0 | 0 | na | 0 | 0 | 0 |  | |  |  |
|  | DemmerWest2 | 0 | 0 | 0 | 0 | na | 0 | 0 | 0 |  | |  |  |
|  | DemmerEast | 0 | 0 | 0 | 0 | na | 0 | 0 | 0 |  | |  |  |
|  | TropJap | 1 | 0.00029 | 0.00029 | 0.00049 | -0.52602 | 0 | 0 | 0 |  | |  |  |
|  | Aromatic | 0 | 0 | 0 | 0 | na | 0 | 0 | 0 |  | |  |  |
|  | Aus | 1 | 0.00122 | 0.00122 | 0.00073 | 1.83822 | 0 | 0 | 0 |  | |  |  |
|  | RufiChina | 5 | 0.00354 | 0.00355 | 0.00316 | 0.35796 | 0 | 0 | 0 |  | |  |  |
|  | RufiIndia | 6 | 0.00307 | 0.00307 | 0.00349 | -0.51685 | 0 | 0 | 0 |  | |  |  |
|  | RufiSEAsia | 7 | 0.00421 | 0.00422 | 0.00436 | -0.09838 | 0 | 0 | 0 |  | |  |  |
|  | Nivara | 2 | 0.00246 | 0.00246 | 0.00202 | 1.03194 | 0 | 0 | 0 |  | |  |  |
|  | Barthii | 3 | 0.00369 | 0.0037 | 0.00303 | 1.18059 | 0 | 0 | 0 |  | |  |  |
|  | Meridionalis | 2 | 0.00307 | 0.00308 | 0.00252 | 1.89306 | 0 | 0 | 0 |  | |  |  |
| **sts025** |  | # seg sites | pi silent | pi (JC) | theta silent | Taj D | polymor. loci | synonymous | replacement | |  | |  |
|  | SC | 0 | 0 | 0 | 0 | na | 0 | 0 | 0 |  | |  |  |
|  | ARred | 2 | 0.00063 | 0.00063 | 0.00107 | -0.5725 | 0 | 0 | 0 |  | |  |  |
|  | CAred | 0 | 0 | 0 | 0 | na | 0 | 0 | 0 |  | |  |  |
|  |  |  |  |  |  |  | x | x | x |  | |  |  |
|  | Tempjap | 0 | 0 | 0 | 0 | na | 0 | 0 | 0 |  | |  |  |
|  |  | 1 | 0.00062 | 0.00062 | 0.00074 | -0.23188 | x | x | x |  | |  |  |
|  |  | 0 | 0 | 0 | 0 | na | x | x | x |  | |  |  |
|  | CAcult | 0 | 0 | 0 | 0 | na | 0 | 0 | 0 |  | |  |  |
|  | ARcult | 1 | 0.00049 | 0.00049 | 0.00082 | -0.68111 | 0 | 0 | 0 |  | |  |  |
|  |  | 1 | 0.00093 | 0.00093 | 0.00101 | -0.19492 | x | x | x |  | |  |  |
|  | indica | 1 | 0.00171 | 0.00172 | 0.00107 | 0.84384 | 0 | 0 | 0 |  | |  |  |
|  | SHWeedy | 0 | 0 | 0 | 0 | na | 0 | 0 | 0 |  | |  |  |
|  | MixWeedy | 1 | 0.00109 | 0.00109 | 0.00108 | 0.01499 | 0 | 0 | 0 |  | |  |  |
|  | BrHWeedy | 0 | 0 | 0 | 0 | na | 0 | 0 | 0 |  | |  |  |
|  | BHWeedy | 0 | 0 | 0 | 0 | na | 0 | 0 | 0 |  | |  |  |
|  | Rufi | 4 | 0.0018 | 0.00181 | 0.00248 | -0.53755 |  |  |  |  | |  |  |
|  |  | 0 | 0 | 0 | 0 | na | x | x | x |  | |  |  |
|  | Glumae | 0 | 0 | 0 | 0 | na | 0 | 0 | 0 |  | |  |  |
|  |  | 2 | 0.00204 | 0.00204 | 0.00167 | 1.89306 | x | x | x |  | |  |  |
|  | glaber | 1 | 0 | 0 | 0 | 0.3335 | 1 | 0 | 1 |  | |  |  |
|  |  |  |  |  |  |  | x | x | x |  | |  |  |
|  | PA_rice | 0 | 0 | 0 | 0 |  | 0 | 0 | 0 |  | |  |  |
|  |  |  |  |  |  |  |  |  |  |  | |  |  |
|  | Updated Seq Set Analyses | | |  |  |  |  |  |  |  | |  |  |
|  |  |  |  |  |  |  |  |  |  |  | |  |  |
|  |  | # seg sites | pi silent | pi (JC) | theta silent | Taj D |  |  |  |  | |  |  |
|  | CAredWell | 0 | 0 | 0 | 0 | na | 0 | 0 | 0 |  | |  |  |
|  | DemmerWest1 | 0 | 0 | 0 | 0 | na | 0 | 0 | 0 |  | |  |  |
|  | DemmerWest2 | 0 | 0 | 0 | 0 | na | 0 | 0 | 0 |  | |  |  |
|  | DemmerEast | 0 | 0 | 0 | 0 | na | 0 | 0 | 0 |  | |  |  |
|  | TropJap | 1 | 0.0004 | 0.0004 | 0.00066 | -0.51132 | 0 | 0 | 0 |  | |  |  |
|  | Aromatic | 0 | 0 | 0 | 0 | na | 0 | 0 | 0 |  | |  |  |
|  | Aus | 1 | 0.00081 | 0.00081 | 0.00096 | -0.34144 | 0 | 0 | 0 |  | |  |  |
|  | RufiChina | 3 | 0.00245 | 0.00245 | 0.00252 | -0.31546 | 1 | 1 | 0 |  | |  |  |
|  | RufiIndia | 5 | 0.00252 | 0.00252 | 0.00386 | -1.0604 | 1 | 1 | 0 |  | |  |  |
|  | RufiSEAsia | 2 | 0.00141 | 0.00141 | 0.0015 | -0.10803 | 0 | 0 | 0 |  | |  |  |
|  | Nivara | 1 | 0.00163 | 0.00163 | 0.00134 | 0.85057 | 0 | 0 | 0 |  | |  |  |
|  | Barthii | 2 | 0.00163 | 0.00163 | 0.00134 | 1.03194 | 1 | 0 | 1 |  | |  |  |
| **sts031** |  | # seg sites | pi silent | pi (JC) | theta silent | Taj D | polymor. loci | synonymous | replacement | |  | |  |
|  | SC | 2 | 0.0047 | 0.00471 | 0.00343 | 1.75324 | 0 | 0 | 0 |  | |  |  |
|  | ARred | 3 | 0.00125 | 0.00125 | 0.00204 | -0.63291 | 0 | 0 | 0 |  | |  |  |
|  | CAred | 0 | 0 | 0 | 0 | na | 0 | 0 | 0 |  | |  |  |
|  |  |  |  |  |  |  | x | x | x |  | |  |  |
|  | Tempjap | 0 | 0 | 0 | 0 | na | 0 | 0 | 0 |  | |  |  |
|  |  | 0 | 0 | 0 | 0 | na | x | x | x |  | |  |  |
|  |  | 0 | 0 | 0 | 0 | na | x | x | x |  | |  |  |
|  | CAcult | 0 | 0 | 0 | 0 | na | 0 | 0 | 0 |  | |  |  |
|  | ARcult | 1 | 0.00119 | 0.00119 | 0.0011 | 0.13869 | 0 | 0 | 0 |  | |  |  |
|  |  | 1 | 0.00119 | 0.00119 | 0.0013 | -0.24805 | x | x | x |  | |  |  |
|  | indica | 3 | 0.00382 | 0.00383 | 0.00275 | 0.54732 | 0 | 0 | 0 |  | |  |  |
|  | SHWeedy | 0 | 0 | 0 | 0 | na | 0 | 0 | 0 |  | |  |  |
|  | MixWeedy | 3 | 0.00504 | 0.00506 | 0.00415 | 0.92759 | 0 | 0 | 0 |  | |  |  |
|  | BrHWeedy | 0 | 0 | 0 | 0 | na | 0 | 0 | 0 |  | |  |  |
|  | BHWeedy | 3 | 0.00335 | 0.00336 | 0.00266 | 0.59294 | 0 | 0 | 0 |  | |  |  |
|  | Rufi | 18 | 0.00536 | 0.00538 | 0.01346 | -1.71814 |  |  |  |  | |  |  |
|  |  | 2 | 0.00522 | 0.00524 | 0.00427 | 1.89306 | x | x | x |  | |  |  |
|  | Glumae | 0 | 0 | 0 | 0 | na | 0 | 0 | 0 |  | |  |  |
|  |  | 0 | 0 | 0 | 0 | na | x | x | x |  | |  |  |
|  | glaber | 0 | 0 | 0 | 0 | na | 0 | 0 | 0 |  | |  |  |
|  |  |  |  |  |  |  | x | x | x |  | |  |  |
|  | PA_rice | 3 | 0.00587 | 0.00589 | 0.0064 |  | 0 | 0 | 0 |  | |  |  |
|  |  |  |  |  |  |  |  |  |  |  | |  |  |
|  | Updated Seq Set Analyses | | |  |  |  |  |  |  |  | |  |  |
|  |  |  |  |  |  |  |  |  |  |  | |  |  |
|  |  | # seg sites | pi silent | pi (JC) | theta silent | Taj D |  |  |  |  | |  |  |
|  | CAredWell | 0 | 0 | 0 | 0 | na | 0 | 0 | 0 |  | |  |  |
|  | DemmerWest1 | 0 | 0 | 0 | 0 | na | 0 | 0 | 0 |  | |  |  |
|  | DemmerWest2 | 0 | 0 | 0 | 0 | na | 0 | 0 | 0 |  | |  |  |
|  | DemmerEast | 0 | 0 | 0 | 0 | na | 0 | 0 | 0 |  | |  |  |
|  | TropJap | 0 | 0 | 0 | 0 | na | 0 | 0 | 0 |  | |  |  |
|  | Aromatic | 0 | 0 | 0 | 0 | na | 0 | 0 | 0 |  | |  |  |
|  | Aus | 1 | 0.00103 | 0.00103 | 0.00123 | -0.43764 | 0 | 0 | 0 |  | |  |  |
|  | RufiChina | 6 | 0.00561 | 0.00563 | 0.00644 | -0.40109 | 1 | 1 | 0 |  | |  |  |
|  | RufiIndia | 8 | 0.00426 | 0.00427 | 0.00777 | -1.35272 | 2 | 2 | 0 |  | |  |  |
|  | RufiSEAsia | 9 | 0.00385 | 0.00386 | 0.00819 | -1.53129 | 0 | 0 | 0 |  | |  |  |
|  | Nivara | 2 | 0.00417 | 0.00419 | 0.00343 | 1.03194 | 0 | 0 | 0 |  | |  |  |
|  | Barthii | 0 | 0 | 0 | 0 | na | 0 | 0 | 0 |  | |  |  |
|  | Meridionalis | 1 | 0.00261 | 0.00261 | 0.00213 | 1.63299 | 0 | 0 | 0 |  | |  |  |
| **sts035** |  | # seg sites | pi silent | pi (JC) | theta silent | Taj D | polymor. loci | synonymous | replacement | |  | |  |
|  | SC | 0 | 0 | 0 | 0 | na | 0 | 0 | 0 |  | |  |  |
|  | ARred | 2 | 0.00276 | 0.00277 | 0.0035 | -0.2924 | 2 | 2 | 0 |  | |  |  |
|  | CAred | 0 | 0 | 0 | 0 | na | 0 | 0 | 0 |  | |  |  |
|  |  | 2 | 0.00662 | 0.00665 | 0.00789 | -0.43764 | x | x | x |  | |  |  |
|  | Tempjap | 2 | 0.00509 | 0.00511 | 0.00473 | 0.14543 | 2 | 2 | 0 |  | |  |  |
|  |  | 1 | 0.00108 | 0.00109 | 0.00242 | -0.81338 | x | x | x |  | |  |  |
|  |  | 0 | 0 | 0 | 0 | na | x | x | x |  | |  |  |
|  | CAcult | 2 | 0.00703 | 0.00707 | 0.00557 | 0.59464 | 2 | 2 | 0 |  | |  |  |
|  | ARcult | 3 | 0.00714 | 0.00717 | 0.00807 | -0.29186 | 3 | 3 | 0 |  | |  |  |
|  |  | 1 | 0.00487 | 0.00489 | 0.00333 | 1.3564 | x | x | x |  | |  |  |
|  | indica | 1 | 0.00458 | 0.00459 | 0.00234 | 1.84834 | 2 | 1 | 1 |  | |  |  |
|  | SHWeedy | 1 | 0.00085 | 0.00086 | 0.00229 | -1.14067 | 2 | 1 | 1 |  | |  |  |
|  | MixWeedy | 2 | 0.00737 | 0.00741 | 0.00711 | 0.69962 | 3 | 2 | 1 |  | |  |  |
|  | BrHWeedy | 0 | 0 | 0 | 0 | na | 0 | 0 | 0 |  | |  |  |
|  | BHWeedy | 0 | 0 | 0 | 0 | na | 0 | 0 | 0 |  | |  |  |
|  | Rufi | 15 | 0.01157 | 0.01166 | 0.02495 | -1.36857 |  |  |  |  | |  |  |
|  |  | 0 | 0 | 0 | 0 | na | x | x | x |  | |  |  |
|  | Glumae | 0 | 0 | 0 | 0 | na | 0 | 0 | 0 |  | |  |  |
|  |  | 1 | 0 | 0 | 0 | 1.63299 | x | x | x |  | |  |  |
|  | glaber | 1 | 0 | 0 | 0 | 0.3335 | 1 | 0 | 1 |  | |  |  |
|  |  | 2 | 0.00536 | 0.00538 | 0.00507 | 0.04709 | x | x | x |  | |  |  |
|  | PA_rice | 0 | 0 | 0 | 0 |  | 0 | 0 | 0 |  | |  |  |
|  |  |  |  |  |  |  |  |  |  |  | |  |  |
|  | Updated Seq Set Analyses | | |  |  |  |  |  |  |  | |  |  |
|  |  |  |  |  |  |  |  |  |  |  | |  |  |
|  |  | # seg sites | pi silent | pi (JC) | theta silent | Taj D |  |  |  |  | |  |  |
|  | CAredWell | 0 | 0 | 0 | 0 | na | 0 | 0 | 0 |  | |  |  |
|  | DemmerWest1 | 0 | 0 | 0 | 0 | na | 0 | 0 | 0 |  | |  |  |
|  | DemmerWest2 | 0 | 0 | 0 | 0 | na | 0 | 0 | 0 |  | |  |  |
|  | DemmerEast | 0 | 0 | 0 | 0 | na | 0 | 0 | 0 |  | |  |  |
|  | TropJap | 1 | 0.00127 | 0.00127 | 0.00216 | -0.94375 | 2 | 1 | 1 |  | |  |  |
|  | Aromatic | 0 | 0 | 0 | 0 | na | 0 | 0 | 0 |  | |  |  |
|  | Aus | 1 | 0.0053 | 0.00532 | 0.00316 | 1.83822 | 2 | 1 | 1 |  | |  |  |
|  | RufiChina | 10 | 0.01236 | 0.01246 | 0.02481 | -1.34555 | 11 | 9 | 2 |  | |  |  |
|  | RufiIndia | 7 | 0.00734 | 0.00737 | 0.01747 | -1.25153 | 10 | 9 | 1 |  | |  |  |
|  | RufiSEAsia | 10 | 0.01337 | 0.01349 | 0.02102 | -0.84143 | 11 | 9 | 2 |  | |  |  |
|  | Nivara | 0 | 0 | 0 | 0 | na | 1 | 0 | 1 |  | |  |  |
|  | Barthii | 1 | 0 | 0 | 0 | 0.85057 | 1 | 0 | 1 |  | |  |  |
|  | Meridionalis | 0 | 0 | 0 | 0 | na | 0 | 0 | 0 |  | |  |  |
| **sts036** |  | # seg sites | pi silent | pi (JC) | theta silent | Taj D | polymor. loci | synonymous | replacement | |  | |  |
|  | SC | 2 | 0.00194 | 0.00194 | 0.00255 | -1.13197 | 1 | 0 | 1 |  | |  |  |
|  | ARred | 2 | 0.00152 | 0.00152 | 0.00101 | 1.66417 | 1 | 0 | 1 |  | |  |  |
|  | CAred | 0 | 0 | 0 | 0 | na | 0 | 0 | 0 |  | |  |  |
|  |  |  |  |  |  |  | x | x | x |  | |  |  |
|  | Tempjap | 1 | 0.00126 | 0.00126 | 0.00155 | -0.27123 | 0 | 0 | 0 |  | |  |  |
|  |  | 1 | 0.00063 | 0.00063 | 0.0014 | -0.81338 | x | x | x |  | |  |  |
|  |  | 0 | 0 | 0 | 0 | na | x | x | x |  | |  |  |
|  | CAcult | 1 | 0.00181 | 0.00182 | 0.0016 | 0.23682 | 0 | 0 | 0 |  | |  |  |
|  | ARcult | 2 | 0.00285 | 0.00285 | 0.00156 | 0.46385 | 1 | 0 | 1 |  | |  |  |
|  |  | 1 | 0.00317 | 0.00318 | 0.00193 | 1.48617 | x | x | x |  | |  |  |
|  | indica | 2 | 0.00292 | 0.00292 | 0.00137 | 1.44283 | 1 | 0 | 1 |  | |  |  |
|  | SHWeedy | 1 | 0 | 0 | 0 | 0.53407 | 2 | 1 | 1 |  | |  |  |
|  | MixWeedy | 1 | 0.0031 | 0.00311 | 0.00206 | 1.30268 | 0 | 0 | 0 |  | |  |  |
|  | BrHWeedy | 1 | 0.0031 | 0.00311 | 0.00206 | 1.30268 | 0 | 0 | 0 |  | |  |  |
|  | BHWeedy | 1 | 0.00048 | 0.00048 | 0.00133 | -0.86644 | 0 | 0 | 0 |  | |  |  |
|  | Rufi | 6 | 0.01385 | 0.01398 | 0.00707 | 1.71925 |  |  |  |  | |  |  |
|  |  | 2 | 0.00388 | 0.00389 | 0.00317 | 1.89306 | x | x | x |  | |  |  |
|  | Glumae | 0 | 0 | 0 | 0 | na | 0 | 0 | 0 |  | |  |  |
|  |  | 0 | 0 | 0 | 0 | na | x | x | x |  | |  |  |
|  | glaber | 0 | 0 | 0 | 0 | na | 0 | 0 | 0 |  | |  |  |
|  |  |  |  |  |  |  | x | x | x |  | |  |  |
|  | PA_rice | 0 | 0 | 0 | 0 |  | 0 | 0 | 0 |  | |  |  |
|  |  |  |  |  |  |  |  |  |  |  | |  |  |
|  | Updated Seq Set Analyses | | |  |  |  |  |  |  |  | |  |  |
|  |  |  |  |  |  |  |  |  |  |  | |  |  |
|  |  | # seg sites | pi silent | pi (JC) | theta silent | Taj D |  |  |  |  | |  |  |
|  | CAredWell | 0 | 0 | 0 | 0 | na | 0 | 0 | 0 |  | |  |  |
|  | DemmerWest1 | 0 | 0 | 0 | 0 | na | 0 | 0 | 0 |  | |  |  |
|  | DemmerWest2 | 0 | 0 | 0 | 0 | na | 0 | 0 | 0 |  | |  |  |
|  | DemmerEast | 0 | 0 | 0 | 0 | na | 0 | 0 | 0 |  | |  |  |
|  | TropJap | 2 | 0.00145 | 0.00145 | 0.00249 | -0.71858 | 1 | 1 | 0 |  | |  |  |
|  | Aromatic | 0 | 0 | 0 | 0 | na | 0 | 0 | 0 |  | |  |  |
|  | Aus | 1 | 0.00307 | 0.00308 | 0.00183 | 1.43413 | 0 | 0 | 0 |  | |  |  |
|  | RufiChina | 10 | 0.00928 | 0.00934 | 0.01242 | -0.65003 | 6 | 3 | 3 |  | |  |  |
|  | RufiIndia | 6 | 0.01691 | 0.0171 | 0.00928 | 1.60343 | 6 | 3 | 3 |  | |  |  |
|  | RufiSEAsia | 17 | 0.01983 | 0.0201 | 0.01901 | 0.14495 | 8 | 4 | 4 |  | |  |  |
|  | Nivara | 2 | 0.0031 | 0.00311 | 0.00255 | 1.03194 | 1 | 0 | 1 |  | |  |  |
|  | Barthii | 0 | 0 | 0 | 0 | na | 0 | 0 | 0 |  | |  |  |
|  | Meridionalis | 16 | 0.05877 | 0.0612 | 0.04808 | 2.26818 | 12 | 10 | 2 |  | |  |  |
| **sts040** |  | # seg sites | pi silent | pi (JC) | theta silent | Taj D | polymor. loci | synonymous | replacement | |  | |  |
|  | SC | 0 | 0 | 0 | 0 | na | 0 | 0 | 0 |  | |  |  |
|  | ARred | 0 | 0 | 0 | 0 | na | 0 | 0 | 0 |  | |  |  |
|  | CAred | 0 | 0 | 0 | 0 | na | 0 | 0 | 0 |  | |  |  |
|  |  |  |  |  |  |  | x | x | x |  | |  |  |
|  | Tempjap | 2 | 0.00067 | 0.00067 | 0.00116 | -0.78344 | 0 | 0 | 0 |  | |  |  |
|  |  | 0 | 0 | 0 | 0 | na | x | x | x |  | |  |  |
|  |  | 0 | 0 | 0 | 0 | na | x | x | x |  | |  |  |
|  | CAcult | 0 | 0 | 0 | 0 | na | 0 | 0 | 0 |  | |  |  |
|  | ARcult | 1 | 0.0004 | 0.0004 | 0.00067 | -0.68111 | 0 | 0 | 0 |  | |  |  |
|  |  | 2 | 0.00076 | 0.00076 | 0.00083 | -0.24805 | x | x | x |  | |  |  |
|  | indica | 1 | 0.00023 | 0.00023 | 0.00058 | -0.84519 | 0 | 0 | 0 |  | |  |  |
|  | SHWeedy | 0 | 0 | 0 | 0 | na | 0 | 0 | 0 |  | |  |  |
|  | MixWeedy | 0 | 0 | 0 | 0 | na | 0 | 0 | 0 |  | |  |  |
|  | BrHWeedy | 0 | 0 | 0 | 0 | na | 0 | 0 | 0 |  | |  |  |
|  | BHWeedy | 0 | 0 | 0 | 0 | na | 0 | 0 | 0 |  | |  |  |
|  | Rufi | 12 | 0.0027 | 0.00271 | 0.00572 | -1.38946 |  |  |  |  | |  |  |
|  |  | 1 | 0.00125 | 0.00125 | 0.00136 | -0.61237 | x | x | x |  | |  |  |
|  | Glumae | 0 | 0 | 0 | 0 | na | 0 | 0 | 0 |  | |  |  |
|  |  | 1 | 0.00166 | 0.00166 | 0.00136 | 1.63299 | x | x | x |  | |  |  |
|  | glaber | 0 | 0 | 0 | 0 | na | 0 | 0 | 0 |  | |  |  |
|  |  |  |  |  |  |  | x | x | x |  | |  |  |
|  | PA_rice | 0 | 0 | 0 | 0 |  | 0 | 0 | 0 |  | |  |  |
|  |  |  |  |  |  |  |  |  |  |  | |  |  |
|  | Updated Seq Set Analyses | | |  |  |  |  |  |  |  | |  |  |
|  |  |  |  |  |  |  |  |  |  |  | |  |  |
|  |  | # seg sites | pi silent | pi (JC) | theta silent | Taj D |  |  |  |  | |  |  |
|  | CAredWell | 0 | 0 | 0 | 0 | na | 0 | 0 | 0 |  | |  |  |
|  | DemmerWest1 | 0 | 0 | 0 | 0 | na | 0 | 0 | 0 |  | |  |  |
|  | DemmerWest2 | 0 | 0 | 0 | 0 | na | 0 | 0 | 0 |  | |  |  |
|  | DemmerEast | 0 | 0 | 0 | 0 | na | 0 | 0 | 0 |  | |  |  |
|  | TropJap | 0 | 0 | 0 | 0 | na | 0 | 0 | 0 |  | |  |  |
|  | Aromatic | 0 | 0 | 0 | 0 | na | 0 | 0 | 0 |  | |  |  |
|  | Aus | 2 | 0.00066 | 0.00066 | 0.00078 | -0.43764 | 1 | 0 | 1 |  | |  |  |
|  | RufiChina | 2 | 0.00121 | 0.00121 | 0.00137 | -0.26344 | 0 | 0 | 0 |  | |  |  |
|  | RufiIndia | 3 | 0.00192 | 0.00192 | 0.00186 | 0.0783 | 0 | 0 | 0 |  | |  |  |
|  | RufiSEAsia | 7 | 0.00313 | 0.00314 | 0.00406 | -0.62548 | 0 | 0 | 0 |  | |  |  |
|  | Nivara | 2 | 0.00216 | 0.00216 | 0.00218 | -0.05002 | 0 | 0 | 0 |  | |  |  |
|  | Barthii | 1 | 0.00133 | 0.00133 | 0.00109 | 0.85057 | 0 | 0 | 0 |  | |  |  |
|  | Meridionalis | 0 | 0 | 0 | 0 | na | 0 | 0 | 0 |  | |  |  |
| **sts041** |  | # seg sites | pi silent | pi (JC) | theta silent | Taj D | polymor. loci | synonymous | replacement | |  | |  |
|  | SC | 0 | 0 | 0 | 0 | na | 0 | 0 | 0 |  | |  |  |
|  | ARred | 0 | 0 | 0 | 0 | na | 0 | 0 | 0 |  | |  |  |
|  | CAred | 0 | 0 | 0 | 0 | na | 0 | 0 | 0 |  | |  |  |
|  |  |  |  |  |  |  | x | x | x |  | |  |  |
|  | Tempjap | 3 | 0.00048 | 0.00048 | 0.00121 | -1.03425 | 2 | 0 | 2 |  | |  |  |
|  |  | 1 | 0.00264 | 0.00265 | 0.00125 | 1.62895 | x | x | x |  | |  |  |
|  |  | 0 | 0 | 0 | 0 | na | x | x | x |  | |  |  |
|  | CAcult | 2 | 0.00162 | 0.00162 | 0.00143 | 0.73766 | 1 | 0 | 1 |  | |  |  |
|  | ARcult | 2 | 0.00211 | 0.00211 | 0.00144 | 1.01428 | 1 | 0 | 1 |  | |  |  |
|  |  | 0 | 0 | 0 | 0 | na | x | x | x |  | |  |  |
|  | indica | 1 | 0 | 0 | 0 | 0.11193 | 1 | 0 | 1 |  | |  |  |
|  | SHWeedy | 0 | 0 | 0 | 0 | na | 0 | 0 | 0 |  | |  |  |
|  | MixWeedy | 0 | 0 | 0 | 0 | na | 0 | 0 | 0 |  | |  |  |
|  | BrHWeedy | 0 | 0 | 0 | 0 | na | 0 | 0 | 0 |  | |  |  |
|  | BHWeedy | 0 | 0 | 0 | 0 | na | 0 | 0 | 0 |  | |  |  |
|  | Rufi | 12 | 0.00459 | 0.0046 | 0.01058 | -1.4002 |  |  |  |  | |  |  |
|  |  | 11 | 0.02773 | 0.02826 | 0.02269 | 2.23308 | x | x | x |  | |  |  |
|  | Glumae | 0 | 0 | 0 | 0 | na | 0 | 0 | 0 |  | |  |  |
|  |  | 0 | 0 | 0 | 0 | na | x | x | x |  | |  |  |
|  | glaber | 0 | 0 | 0 | 0 | na | 0 | 0 | 0 |  | |  |  |
|  |  |  |  |  |  |  | x | x | x |  | |  |  |
|  | PA_rice | 0 | 0 | 0 | 0 |  | 0 | 0 | 0 |  | |  |  |
|  |  |  |  |  |  |  |  |  |  |  | |  |  |
|  | Updated Seq Set Analyses | | |  |  |  |  |  |  |  | |  |  |
|  |  |  |  |  |  |  |  |  |  |  | |  |  |
|  |  | # seg sites | pi silent | pi (JC) | theta silent | Taj D |  |  |  |  | |  |  |
|  | CAredWell | 0 | 0 | 0 | 0 | na | 0 | 0 | 0 |  | |  |  |
|  | DemmerWest1 | 0 | 0 | 0 | 0 | na | 0 | 0 | 0 |  | |  |  |
|  | DemmerWest2 | 0 | 0 | 0 | 0 | na | 0 | 0 | 0 |  | |  |  |
|  | DemmerEast | 0 | 0 | 0 | 0 | na | 0 | 0 | 0 |  | |  |  |
|  | TropJap | 2 | 0.00226 | 0.00227 | 0.00112 | 0.28415 | 1 | 0 | 1 |  | |  |  |
|  | Aromatic | 0 | 0 | 0 | 0 | na | 0 | 0 | 0 |  | |  |  |
|  | Aus | 0 | 0 | 0 | 0 | na | 0 | 0 | 0 |  | |  |  |
|  | RufiChina | 3 | 0.00263 | 0.00264 | 0.00143 | 1.57686 | 2 | 0 | 2 |  | |  |  |
|  | RufiIndia | 3 | 0.00126 | 0.00126 | 0.00258 | -1.18531 | 2 | 1 | 1 |  | |  |  |
|  | RufiSEAsia | 12 | 0.00607 | 0.00609 | 0.01007 | -1.14409 | 8 | 4 | 4 |  | |  |  |
|  | Nivara | 11 | 0.02218 | 0.02252 | 0.01822 | 1.31709 | 8 | 5 | 3 |  | |  |  |
|  | Barthii | 0 | 0 | 0 | 0 | na | 0 | 0 | 0 |  | |  |  |
|  | Meridionalis | 2 | 0 | 0 | 0 | 1.89306 | 1 | 0 | 1 |  | |  |  |
| **sts046** |  | # seg sites | pi silent | pi (JC) | theta silent | Taj D | polymor. loci | synonymous | replacement | |  | |  |
|  | SC | 3 | 0.00851 | 0.00856 | 0.00762 | 0.60031 | 0 | 0 | 0 |  | |  |  |
|  | ARred | 0 | 0 | 0 | 0 | na | 0 | 0 | 0 |  | |  |  |
|  | CAred | 0 | 0 | 0 | 0 | na | 0 | 0 | 0 |  | |  |  |
|  |  |  |  |  |  |  | x | x | x |  | |  |  |
|  | Tempjap | 2 | 0.00104 | 0.00104 | 0.00261 | -1.11882 | 0 | 0 | 0 |  | |  |  |
|  |  | 2 | 0.00474 | 0.00475 | 0.00271 | 1.45898 | x | x | x |  | |  |  |
|  |  | 0 | 0 | 0 | 0 | na | x | x | x |  | |  |  |
|  | CAcult | 1 | 0.00097 | 0.00097 | 0.00154 | -0.64112 | 0 | 0 | 0 |  | |  |  |
|  | ARcult | 2 | 0.00179 | 0.00179 | 0.00301 | -0.88989 | 0 | 0 | 0 |  | |  |  |
|  |  | 1 | 0.0017 | 0.0017 | 0.00186 | -0.19492 | x | x | x |  | |  |  |
|  | indica | 1 | 0.00141 | 0.00141 | 0.0013 | 0.14816 | 1 | 1 | 0 |  | |  |  |
|  | SHWeedy | 1 | 0.00051 | 0.00051 | 0.00135 | -0.86025 | 0 | 0 | 0 |  | |  |  |
|  | MixWeedy | 0 | 0 | 0 | 0 | na | 0 | 0 | 0 |  | |  |  |
|  | BrHWeedy | 0 | 0 | 0 | 0 | na | 1 | 0 | 1 |  | |  |  |
|  | BHWeedy | 1 | 0 | 0 | 0 | -0.85321 | 0 | 0 | 0 |  | |  |  |
|  | Rufi | 13 | 0.00359 | 0.0036 | 0.01094 | -1.88843 |  |  |  |  | |  |  |
|  |  | 1 | 0.00374 | 0.00375 | 0.00306 | 1.63299 | x | x | x |  | |  |  |
|  | Glumae | 1 | 0.00374 | 0.00375 | 0.00306 | 1.63299 | 1 | 1 | 0 |  | |  |  |
|  |  | 3 | 0.01123 | 0.01131 | 0.00918 | 2.01187 | x | x | x |  | |  |  |
|  | glaber | 0 | 0 | 0 | 0 | na | 0 | 0 | 0 |  | |  |  |
|  |  |  |  |  |  |  | x | x | x |  | |  |  |
|  | PA_rice | 0 | 0 | 0 | 0 |  | 0 | 0 | 0 |  | |  |  |
|  |  |  |  |  |  |  |  |  |  |  | |  |  |
|  | Updated Seq Set Analyses | | |  |  |  |  |  |  |  | |  |  |
|  |  |  |  |  |  |  |  |  |  |  | |  |  |
|  |  | # seg sites | pi silent | pi (JC) | theta silent | Taj D |  |  |  |  | |  |  |
|  | CAredWell | 0 | 0 | 0 | 0 | na | 0 | 0 | 0 |  | |  |  |
|  | DemmerWest1 | 0 | 0 | 0 | 0 | na | 0 | 0 | 0 |  | |  |  |
|  | DemmerWest2 | 0 | 0 | 0 | 0 | na | 0 | 0 | 0 |  | |  |  |
|  | DemmerEast | 0 | 0 | 0 | 0 | na | 0 | 0 | 0 |  | |  |  |
|  | TropJap | 2 | 0.0052 | 0.00522 | 0.00241 | 1.98341 | 0 | 0 | 0 |  | |  |  |
|  | Aromatic | 0 | 0 | 0 | 0 | na | 0 | 0 | 0 |  | |  |  |
|  | Aus | 1 | 0.00148 | 0.00148 | 0.00176 | -0.34144 | 0 | 0 | 0 |  | |  |  |
|  | RufiChina | 3 | 0.00532 | 0.00534 | 0.00462 | -0.16737 | 3 | 3 | 0 |  | |  |  |
|  | RufiIndia | 7 | 0.00682 | 0.00686 | 0.0073 | -0.66446 | 6 | 4 | 2 |  | |  |  |
|  | RufiSEAsia | 2 | 0.00055 | 0.00055 | 0.00137 | -1.11882 | 2 | 1 | 1 |  | |  |  |
|  | Nivara | 2 | 0.00599 | 0.00601 | 0.00492 | 1.03194 | 1 | 1 | 0 |  | |  |  |
|  | Barthii | 3 | 0.00898 | 0.00903 | 0.00737 | 1.12414 | 1 | 1 | 0 |  | |  |  |
|  | Meridionalis | 0 | 0 | 0 | 0 | na | 0 | 0 | 0 |  | |  |  |
| **sts047** |  | # seg sites | pi silent | pi (JC) | theta silent | Taj D | polymor. loci | synonymous | replacement | |  | |  |
|  | SC | 0 | 0 | 0 | 0 | na | 0 | 0 | 0 |  | |  |  |
|  | ARred | 2 | 0.00068 | 0.00068 | 0.0011 | -0.53249 | 1 | 1 | 0 |  | |  |  |
|  | CAred | 0 | 0 | 0 | 0 | na | 0 | 0 | 0 |  | |  |  |
|  |  | 0 | 0 | 0 | 0 | na | x | x | x |  | |  |  |
|  | Tempjap | 2 | 0.00085 | 0.00085 | 0.00147 | -0.78344 | 2 | 2 | 0 |  | |  |  |
|  |  | 1 | 0.00034 | 0.00034 | 0.00076 | -0.81338 | x | x | x |  | |  |  |
|  |  | 0 | 0 | 0 | 0 | na | x | x | x |  | |  |  |
|  | CAcult | 2 | 0.00264 | 0.00265 | 0.00174 | 1.1667 | 2 | 2 | 0 |  | |  |  |
|  | ARcult | 2 | 0.00238 | 0.00239 | 0.00169 | 0.89527 | 2 | 2 | 0 |  | |  |  |
|  |  | 1 | 0.00096 | 0.00096 | 0.00105 | -0.19492 | x | x | x |  | |  |  |
|  | indica | 3 | 0.00059 | 0.00059 | 0.00147 | -1.29256 | 2 | 1 | 1 |  | |  |  |
|  | SHWeedy | 0 | 0 | 0 | 0 | na | 0 | 0 | 0 |  | |  |  |
|  | MixWeedy | 2 | 0.00281 | 0.00282 | 0.00224 | 0.83017 | 1 | 1 | 0 |  | |  |  |
|  | BrHWeedy | 0 | 0 | 0 | 0 | na | 0 | 0 | 0 |  | |  |  |
|  | BHWeedy | 2 | 0.00311 | 0.00311 | 0.00146 | 2.02647 | 1 | 1 | 0 |  | |  |  |
|  | Rufi | 20 | 0.00293 | 0.00294 | 0.00974 | -1.64931 |  |  |  |  | |  |  |
|  |  | 0 | 0 | 0 | 0 | na | x | x | x |  | |  |  |
|  | Glumae | 1 | 0.00211 | 0.00211 | 0.00173 | 1.63299 | 0 | 0 | 0 |  | |  |  |
|  |  | 0 | 0 | 0 | 0 | na | x | x | x |  | |  |  |
|  | glaber | 0 | 0 | 0 | 0 | na | 0 | 0 | 0 |  | |  |  |
|  |  | 3 | 0.00221 | 0.00221 | 0.0024 | -0.18152 | x | x | x |  | |  |  |
|  | PA_rice | 0 | 0 | 0 | 0 |  | 0 | 0 | 0 |  | |  |  |
|  |  |  |  |  |  |  |  |  |  |  | |  |  |
|  | Updated Seq Set Analyses | | |  |  |  |  |  |  |  | |  |  |
|  |  |  |  |  |  |  |  |  |  |  | |  |  |
|  |  | # seg sites | pi silent | pi (JC) | theta silent | Taj D |  |  |  |  | |  |  |
|  | CAredWell | 0 | 0 | 0 | 0 | na | 0 | 0 | 0 |  | |  |  |
|  | DemmerWest1 | 0 | 0 | 0 | 0 | na | 0 | 0 | 0 |  | |  |  |
|  | DemmerWest2 | 0 | 0 | 0 | 0 | na | 0 | 0 | 0 |  | |  |  |
|  | DemmerEast | 0 | 0 | 0 | 0 | na | 0 | 0 | 0 |  | |  |  |
|  | TropJap | 2 | 0.0008 | 0.0008 | 0.00136 | -0.70057 | 2 | 2 | 0 |  | |  |  |
|  | Aromatic | 0 | 0 | 0 | 0 | na | 0 | 0 | 0 |  | |  |  |
|  | Aus | 1 | 0.00083 | 0.00083 | 0.00099 | -0.34144 | 0 | 0 | 0 |  | |  |  |
|  | RufiChina | 6 | 0.00208 | 0.00208 | 0.0026 | -0.36034 | 6 | 3 | 3 |  | |  |  |
|  | RufiIndia | 6 | 0.0017 | 0.0017 | 0.00314 | -0.70311 | 3 | 1 | 2 |  | |  |  |
|  | RufiSEAsia | 9 | 0.00192 | 0.00192 | 0.00452 | -1.10183 | 7 | 4 | 3 |  | |  |  |
|  | Nivara | 1 | 0.00169 | 0.00169 | 0.00139 | 0.85057 | 0 | 0 | 0 |  | |  |  |
|  | Barthii | 0 | 0 | 0 | 0 | na | 0 | 0 | 0 |  | |  |  |
|  | Meridionalis | 1 | 0.00211 | 0.00211 | 0.00173 | 1.63299 | 0 | 0 | 0 |  | |  |  |
| **sts051** |  | # seg sites | pi silent | pi (JC) | theta silent | Taj D | polymor. loci | synonymous | replacement | |  | |  |
|  | SC | 0 | 0 | 0 | 0 | na | 0 | 0 | 0 |  | |  |  |
|  | ARred | 3 | 0.00092 | 0.00092 | 0.00115 | -0.32702 | 0 | 0 | 0 |  | |  |  |
|  | CAred | 0 | 0 | 0 | 0 | na | 0 | 0 | 0 |  | |  |  |
|  |  | 3 | 0.00202 | 0.00202 | 0.00241 | -0.49373 | x | x | x |  | |  |  |
|  | Tempjap | 2 | 0.00041 | 0.00041 | 0.00102 | -1.11882 | 0 | 0 | 0 |  | |  |  |
|  |  | 2 | 0.00048 | 0.00048 | 0.00106 | -1.07337 | x | x | x |  | |  |  |
|  |  | 0 | 0 | 0 | 0 | na | x | x | x |  | |  |  |
|  | CAcult | 2 | 0.00186 | 0.00186 | 0.00123 | 1.1667 | 0 | 0 | 0 |  | |  |  |
|  | ARcult | 2 | 0.00212 | 0.00212 | 0.00122 | 1.60933 | 0 | 0 | 0 |  | |  |  |
|  |  | 2 | 0.00133 | 0.00134 | 0.00146 | -0.24805 | x | x | x |  | |  |  |
|  | indica | 2 | 0.00121 | 0.00121 | 0.00102 | 0.33448 | 0 | 0 | 0 |  | |  |  |
|  | SHWeedy | 0 | 0 | 0 | 0 | na | 0 | 0 | 0 |  | |  |  |
|  | MixWeedy | 3 | 0.00274 | 0.00274 | 0.00233 | 0.62422 | 0 | 0 | 0 |  | |  |  |
|  | BrHWeedy | 0 | 0 | 0 | 0 | na | 0 | 0 | 0 |  | |  |  |
|  | BHWeedy | 2 | 0.00211 | 0.00211 | 0.00099 | 2.02647 | 0 | 0 | 0 |  | |  |  |
|  | Rufi | 16 | 0.00301 | 0.00301 | 0.00677 | -1.54387 |  |  |  |  | |  |  |
|  |  | 0 | 0 | 0 | 0 | na | x | x | x |  | |  |  |
|  | Glumae | 0 | 0 | 0 | 0 | na | 0 | 0 | 0 |  | |  |  |
|  |  | 1 | 0.00147 | 0.00147 | 0.0012 | 1.63299 | x | x | x |  | |  |  |
|  | glaber | 0 | 0 | 0 | 0 | na | 0 | 0 | 0 |  | |  |  |
|  |  | 3 | 0.00137 | 0.00138 | 0.0017 | -0.45642 | x | x | x |  | |  |  |
|  | PA_rice | 0 | 0 | 0 | 0 |  | 0 | 0 | 0 |  | |  |  |
|  |  |  |  |  |  |  |  |  |  |  | |  |  |
|  | Updated Seq Set Analyses | | |  |  |  |  |  |  |  | |  |  |
|  |  |  |  |  |  |  |  |  |  |  | |  |  |
|  |  | # seg sites | pi silent | pi (JC) | theta silent | Taj D |  |  |  |  | |  |  |
|  | CAredWell | 0 | 0 | 0 | 0 | na | 0 | 0 | 0 |  | |  |  |
|  | DemmerWest1 | 0 | 0 | 0 | 0 | na | 0 | 0 | 0 |  | |  |  |
|  | DemmerWest2 | 0 | 0 | 0 | 0 | na | 0 | 0 | 0 |  | |  |  |
|  | DemmerEast | 0 | 0 | 0 | 0 | na | 0 | 0 | 0 |  | |  |  |
|  | TropJap | 2 | 0.00058 | 0.00058 | 0.00095 | -0.68063 | 0 | 0 | 0 |  | |  |  |
|  | Aromatic | 0 | 0 | 0 | 0 | na | 0 | 0 | 0 |  | |  |  |
|  | Aus | 2 | 0.00116 | 0.00116 | 0.00138 | -0.43764 | 0 | 0 | 0 |  | |  |  |
|  | RufiChina | 3 | 0.00234 | 0.00235 | 0.00181 | 0.76051 | 0 | 0 | 0 |  | |  |  |
|  | RufiIndia | 7 | 0.00222 | 0.00222 | 0.00384 | -1.22903 | 0 | 0 | 0 |  | |  |  |
|  | RufiSEAsia | 8 | 0.00233 | 0.00233 | 0.00409 | -1.21678 | 0 | 0 | 0 |  | |  |  |
|  | Nivara | 2 | 0.00235 | 0.00236 | 0.00193 | 1.03194 | 0 | 0 | 0 |  | |  |  |
|  | Barthii | 1 | 0.00117 | 0.00117 | 0.00096 | 0.85057 | 0 | 0 | 0 |  | |  |  |
|  | Meridionalis | 0 | 0 | 0 | 0 | na | 0 | 0 | 0 |  | |  |  |
| **sts052** |  | # seg sites | pi silent | pi (JC) | theta silent | Taj D | polymor. loci | synonymous | replacement | |  | |  |
|  | SC | 2 | 0.00201 | 0.00202 | 0.00165 | 1.12414 | 3 | 1 | 2 |  | |  |  |
|  | ARred | 2 | 0.0009 | 0.0009 | 0.00066 | 0.51827 | 2 | 1 | 1 |  | |  |  |
|  | CAred | 0 | 0 | 0 | 0 | na | 0 | 0 | 0 |  | |  |  |
|  |  |  |  |  |  |  | x | x | x |  | |  |  |
|  | Tempjap | 0 | 0 | 0 | 0 | na | 1 | 0 | 1 |  | |  |  |
|  |  | 0 | 0 | 0 | 0 | na | x | x | x |  | |  |  |
|  |  | 0 | 0 | 0 | 0 | na | x | x | x |  | |  |  |
|  | CAcult | 0 | 0 | 0 | 0 | na | 0 | 0 | 0 |  | |  |  |
|  | ARcult | 0 | 0 | 0 | 0 | na | 0 | 0 | 0 |  | |  |  |
|  |  | 0 | 0 | 0 | 0 | na | x | x | x |  | |  |  |
|  | indica | 2 | 0.00186 | 0.00187 | 0.00088 | 2.42058 | 3 | 1 | 2 |  | |  |  |
|  | SHWeedy | 2 | 0.00032 | 0.00032 | 0.00086 | -1.31955 | 3 | 1 | 2 |  | |  |  |
|  | MixWeedy | 2 | 0.00134 | 0.00134 | 0.00133 | 0.02107 | 3 | 1 | 2 |  | |  |  |
|  | BrHWeedy | 0 | 0 | 0 | 0 | na | 0 | 0 | 0 |  | |  |  |
|  | BHWeedy | 1 | 0 | 0 | 0 | 1.18518 | 1 | 0 | 1 |  | |  |  |
|  | Rufi | 24 | 0.00554 | 0.00556 | 0.01785 | -1.96414 |  |  |  |  | |  |  |
|  |  | 0 | 0 | 0 | 0 | na | x | x | x |  | |  |  |
|  | Glumae | 10 | 0.01983 | 0.0201 | 0.01885 | 0.95719 | 7 | 4 | 3 |  | |  |  |
|  |  | 0 | 0 | 0 | 0 | na | x | x | x |  | |  |  |
|  | glaber | 0 | 0 | 0 | 0 | na | 0 | 0 | 0 |  | |  |  |
|  |  |  |  |  |  |  | x | x | x |  | |  |  |
|  | PA_rice | 1 | 0.00253 | 0.00253 | 0.00207 |  | 1 | 1 | 0 |  | |  |  |
|  |  |  |  |  |  |  |  |  |  |  | |  |  |
|  | Updated Seq Set Analyses | | |  |  |  |  |  |  |  | |  |  |
|  |  |  |  |  |  |  |  |  |  |  | |  |  |
|  |  | # seg sites | pi silent | pi (JC) | theta silent | Taj D |  |  |  |  | |  |  |
|  | CAredWell | 0 | 0 | 0 | 0 | na | 0 | 0 | 0 |  | |  |  |
|  | DemmerWest1 | 0 | 0 | 0 | 0 | na | 0 | 0 | 0 |  | |  |  |
|  | DemmerWest2 | 0 | 0 | 0 | 0 | na | 0 | 0 | 0 |  | |  |  |
|  | DemmerEast | 0 | 0 | 0 | 0 | na | 0 | 0 | 0 |  | |  |  |
|  | TropJap | 2 | 0.00026 | 0.00026 | 0.00082 | -0.99876 | 3 | 1 | 2 |  | |  |  |
|  | Aromatic | 0 | 0 | 0 | 0 | na | 0 | 0 | 0 |  | |  |  |
|  | Aus | 0 | 0 | 0 | 0 | na | 0 | 0 | 0 |  | |  |  |
|  | RufiChina | 3 | 0.00172 | 0.00172 | 0.00104 | -0.48631 | 5 | 2 | 3 |  | |  |  |
|  | RufiIndia | 10 | 0.0035 | 0.0035 | 0.00472 | -0.65763 | 9 | 3 | 6 |  | |  |  |
|  | RufiSEAsia | 9 | 0.0037 | 0.0037 | 0.00263 | -0.73353 | 11 | 4 | 7 |  | |  |  |
|  | Nivara | 1 | 0 | 0 | 0 | 1.03194 | 2 | 0 | 2 |  | |  |  |
|  | Barthii | 1 | 0.00201 | 0.00202 | 0.00165 | 0.85057 | 0 | 0 | 0 |  | |  |  |
|  | Meridionalis | |  |  |  |  | 6 | 6 | 0 |  | |  |  |
| **sts059** |  | # seg sites | pi silent | pi (JC) | theta silent | Taj D | polymor. loci | synonymous | replacement | |  | |  |
|  | SC | 0 | 0 | 0 | 0 | na | 0 | 0 | 0 |  | |  |  |
|  | ARred | 0 | 0 | 0 | 0 | na | 0 | 0 | 0 |  | |  |  |
|  | CAred | 0 | 0 | 0 | 0 | na | 0 | 0 | 0 |  | |  |  |
|  |  |  |  |  |  |  | x | x | x |  | |  |  |
|  | Tempjap | 1 | 0 | 0 | 0 | 1.35055 | 1 | 0 | 1 |  | |  |  |
|  |  | 0 | 0 | 0 | 0 | na | x | x | x |  | |  |  |
|  |  | 0 | 0 | 0 | 0 | na | x | x | x |  | |  |  |
|  | CAcult | 0 | 0 | 0 | 0 | na | 0 | 0 | 0 |  | |  |  |
|  | ARcult | 1 | 0 | 0 | 0 | -0.68111 | 1 | 0 | 1 |  | |  |  |
|  |  | 0 | 0 | 0 | 0 | na | x | x | x |  | |  |  |
|  | indica | 0 | 0 | 0 | 0 | na | 0 | 0 | 0 |  | |  |  |
|  | SHWeedy | 0 | 0 | 0 | 0 | na | 0 | 0 | 0 |  | |  |  |
|  | MixWeedy | 0 | 0 | 0 | 0 | na | 0 | 0 | 0 |  | |  |  |
|  | BrHWeedy | 0 | 0 | 0 | 0 | na | 0 | 0 | 0 |  | |  |  |
|  | BHWeedy | 0 | 0 | 0 | 0 | na | 0 | 0 | 0 |  | |  |  |
|  | Rufi | 14 | 0.00178 | 0.00179 | 0.00795 | -2.11571 |  |  |  |  | |  |  |
|  |  | 0 | 0 | 0 | 0 | na | x | x | x |  | |  |  |
|  | Glumae | 2 | 0.00458 | 0.00459 | 0.00375 | 1.89306 | 0 | 0 | 0 |  | |  |  |
|  |  | 0 | 0 | 0 | 0 | na | x | x | x |  | |  |  |
|  | glaber | 0 | 0 | 0 | 0 | na | 0 | 0 | 0 |  | |  |  |
|  |  |  |  |  |  |  | x | x | x |  | |  |  |
|  | PA_rice | 0 | 0 | 0 | 0 |  | 0 | 0 | 0 |  | |  |  |
|  |  |  |  |  |  |  |  |  |  |  | |  |  |
|  | Updated Seq Set Analyses | | |  |  |  |  |  |  |  | |  |  |
|  |  |  |  |  |  |  |  |  |  |  | |  |  |
|  |  | # seg sites | pi silent | pi (JC) | theta silent | Taj D |  |  |  |  | |  |  |
|  | CAredWell | 0 | 0 | 0 | 0 | na | 0 | 0 | 0 |  | |  |  |
|  | DemmerWest1 | 0 | 0 | 0 | 0 | na | 0 | 0 | 0 |  | |  |  |
|  | DemmerWest2 | 0 | 0 | 0 | 0 | na | 0 | 0 | 0 |  | |  |  |
|  | DemmerEast | 0 | 0 | 0 | 0 | na | 0 | 0 | 0 |  | |  |  |
|  | TropJap | 0 | 0 | 0 | 0 | na | 0 | 0 | 0 |  | |  |  |
|  | Aromatic | 0 | 0 | 0 | 0 | na | 0 | 0 | 0 |  | |  |  |
|  | Aus | 0 | 0 | 0 | 0 | na | 0 | 0 | 0 |  | |  |  |
|  | RufiChina | 0 | 0 | 0 | 0 | na | 0 | 0 | 0 |  | |  |  |
|  | RufiIndia | 2 | 0.00154 | 0.00155 | 0.00171 | -0.19026 | 0 | 0 | 0 |  | |  |  |
|  | RufiSEAsia | 4 | 0.00082 | 0.00082 | 0.00323 | -1.76151 | 0 | 0 | 0 |  | |  |  |
|  | Nivara | 1 | 0.00183 | 0.00183 | 0.0015 | 0.85057 | 0 | 0 | 0 |  | |  |  |
|  | Barthii | 0 | 0 | 0 | 0 | na | 0 | 0 | 0 |  | |  |  |
|  | Meridionalis | 0 | 0 | 0 | 0 | na | 0 | 0 | 0 |  | |  |  |
| **sts060** |  | # seg sites | pi silent | pi (JC) | theta silent | Taj D | polymor. loci | synonymous | replacement | |  | |  |
|  | SC | 0 | 0 | 0 | 0 | na | 0 | 0 | 0 |  | |  |  |
|  | ARred | 2 | 0.0001 | 0.0001 | 0.00054 | -0.86768 | 1 | 0 | 1 |  | |  |  |
|  | CAred | 0 | 0 | 0 | 0 | na | 0 | 0 | 0 |  | |  |  |
|  |  | 2 | 0.00166 | 0.00166 | 0.00137 | 1.03194 | x | x | x |  | |  |  |
|  | Tempjap | 5 | 0.0034 | 0.00341 | 0.00297 | 0.67278 | 3 | 1 | 2 |  | |  |  |
|  |  | 4 | 0.00233 | 0.00234 | 0.00229 | 0.62845 | x | x | x |  | |  |  |
|  |  | 0 | 0 | 0 | 0 | na | x | x | x |  | |  |  |
|  | CAcult | 5 | 0.00373 | 0.00374 | 0.00338 | 0.55496 | 2 | 1 | 1 |  | |  |  |
|  | ARcult | 4 | 0.00478 | 0.00479 | 0.00252 | 2.44555 | 1 | 0 | 1 |  | |  |  |
|  |  | 4 | 0.00165 | 0.00165 | 0.00109 | 1.47066 | x | x | x |  | |  |  |
|  | indica | 4 | 0.00188 | 0.00189 | 0.00228 | -0.03104 | 2 | 1 | 1 |  | |  |  |
|  | SHWeedy | 0 | 0 | 0 | 0 | na | 0 | 0 | 0 |  | |  |  |
|  | MixWeedy | 4 | 0.00328 | 0.00329 | 0.00327 | 0.02248 | 1 | 0 | 1 |  | |  |  |
|  | BrHWeedy | 0 | 0 | 0 | 0 | na | 0 | 0 | 0 |  | |  |  |
|  | BHWeedy | 2 | 0.00048 | 0.00048 | 0.00069 | -0.55421 | 1 | 0 | 1 |  | |  |  |
|  | Rufi | 14 | 0.00247 | 0.00248 | 0.00579 | -1.59126 |  |  |  |  | |  |  |
|  |  | 0 | 0 | 0 | 0 | na | x | x | x |  | |  |  |
|  | Glumae |  |  |  |  |  |  |  |  |  | |  |  |
|  |  |  |  |  |  |  | x | x | x |  | |  |  |
|  | glaber |  |  |  |  |  |  |  |  |  | |  |  |
|  |  | 6 | 0.00182 | 0.00182 | 0.00234 | -1.12484 | x | x | x |  | |  |  |
|  | PA_rice | 2 | 0.00412 | 0.00413 | 0.00337 |  | 0 | 0 | 0 |  | |  |  |
|  |  |  |  |  |  |  |  |  |  |  | |  |  |
|  | Updated Seq Set Analyses | | |  |  |  |  |  |  |  | |  |  |
|  |  |  |  |  |  |  |  |  |  |  | |  |  |
|  |  | # seg sites | pi silent | pi (JC) | theta silent | Taj D |  |  |  |  | |  |  |
|  | CAredWell | 0 | 0 | 0 | 0 | na | 0 | 0 | 0 |  | |  |  |
|  | DemmerWest1 | 0 | 0 | 0 | 0 | na | 0 | 0 | 0 |  | |  |  |
|  | DemmerWest2 | 0 | 0 | 0 | 0 | na | 0 | 0 | 0 |  | |  |  |
|  | DemmerEast | 0 | 0 | 0 | 0 | na | 0 | 0 | 0 |  | |  |  |
|  | TropJap | 4 | 0.00171 | 0.00171 | 0.00201 | -0.07379 | 1 | 0 | 1 |  | |  |  |
|  | Aromatic | 0 | 0 | 0 | 0 | na | 0 | 0 | 0 |  | |  |  |
|  | Aus | 4 | 0.0015 | 0.0015 | 0.00102 | 1.63255 | 3 | 0 | 3 |  | |  |  |
|  | RufiChina | 6 | 0.00231 | 0.00231 | 0.00338 | -0.50705 | 2 | 0 | 2 |  | |  |  |
|  | RufiIndia | 2 | 0.00164 | 0.00164 | 0.00135 | 1.12414 | 1 | 0 | 1 |  | |  |  |
|  | RufiSEAsia | 8 | 0.00263 | 0.00264 | 0.00374 | -0.66687 | 4 | 0 | 4 |  | |  |  |
|  | Nivara | 0 | 0 | 0 | 0 | na | 0 | 0 | 0 |  | |  |  |
|  | Barthii |  |  |  |  |  |  |  |  |  | |  |  |
|  | Meridionalis | 3 | 0.00205 | 0.00206 | 0.00168 | 2.08033 | 4 | 1 | 3 |  | |  |  |
| **sts061** |  | # seg sites | pi silent | pi (JC) | theta silent | Taj D | polymor. loci | synonymous | replacement | |  | |  |
|  | SC | 0 | 0 | 0 | 0 | na | 0 | 0 | 0 |  | |  |  |
|  | ARred | 0 | 0 | 0 | 0 | na | 0 | 0 | 0 |  | |  |  |
|  | CAred | 0 | 0 | 0 | 0 | na | 0 | 0 | 0 |  | |  |  |
|  |  |  |  |  |  |  | x | x | x |  | |  |  |
|  | Tempjap | 3 | 0.00097 | 0.00097 | 0.00242 | -1.29256 | 0 | 0 | 0 |  | |  |  |
|  |  | 0 | 0 | 0 | 0 | na | x | x | x |  | |  |  |
|  |  | 3 | 0.00571 | 0.00573 | 0.00347 | 2.12303 | x | x | x |  | |  |  |
|  | CAcult | 0 | 0 | 0 | 0 | na | 0 | 0 | 0 |  | |  |  |
|  | ARcult | 1 | 0.00103 | 0.00103 | 0.00095 | 0.13869 | 0 | 0 | 0 |  | |  |  |
|  |  | 0 | 0 | 0 | 0 | na | x | x | x |  | |  |  |
|  | indica | 0 | 0 | 0 | 0 | na | 0 | 0 | 0 |  | |  |  |
|  | SHWeedy | 0 | 0 | 0 | 0 | na | 0 | 0 | 0 |  | |  |  |
|  | MixWeedy | 1 | 0.00124 | 0.00124 | 0.00123 | 0.01499 | 0 | 0 | 0 |  | |  |  |
|  | BrHWeedy | 0 | 0 | 0 | 0 | na | 0 | 0 | 0 |  | |  |  |
|  | BHWeedy | 0 | 0 | 0 | 0 | na | 0 | 0 | 0 |  | |  |  |
|  | Rufi | 10 | 0.00161 | 0.00161 | 0.00398 | -1.7882 |  |  |  |  | |  |  |
|  |  | 0 | 0 | 0 | 0 | na | x | x | x |  | |  |  |
|  | Glumae | 0 | 0 | 0 | 0 | na | 0 | 0 | 0 |  | |  |  |
|  |  | 1 | 0 | 0 | 0 | 1.63299 | x | x | x |  | |  |  |
|  | glaber | 0 | 0 | 0 | 0 | na | 0 | 0 | 0 |  | |  |  |
|  |  |  |  |  |  |  | x | x | x |  | |  |  |
|  | PA_rice | 0 | 0 | 0 | 0 |  | 0 | 0 | 0 |  | |  |  |
|  |  |  |  |  |  |  |  |  |  |  | |  |  |
|  | Updated Seq Set Analyses | | |  |  |  |  |  |  |  | |  |  |
|  |  |  |  |  |  |  |  |  |  |  | |  |  |
|  |  | # seg sites | pi silent | pi (JC) | theta silent | Taj D |  |  |  |  | |  |  |
|  | CAredWell | 0 | 0 | 0 | 0 | na | 0 | 0 | 0 |  | |  |  |
|  | DemmerWest1 | 0 | 0 | 0 | 0 | na | 0 | 0 | 0 |  | |  |  |
|  | DemmerWest2 | 0 | 0 | 0 | 0 | na | 0 | 0 | 0 |  | |  |  |
|  | DemmerEast | 0 | 0 | 0 | 0 | na | 0 | 0 | 0 |  | |  |  |
|  | TropJap | 0 | 0 | 0 | 0 | na | 0 | 0 | 0 |  | |  |  |
|  | Aromatic | 3 | 0.00571 | 0.00573 | 0.00347 | 2.12303 | 0 | 0 | 0 |  | |  |  |
|  | Aus | 0 | 0 | 0 | 0 | na | 0 | 0 | 0 |  | |  |  |
|  | RufiChina | 3 | 0.00315 | 0.00315 | 0.00285 | 0.27069 | 0 | 0 | 0 |  | |  |  |
|  | RufiIndia | 2 | 0.00084 | 0.00084 | 0.00172 | -1.36672 | 1 | 1 | 0 |  | |  |  |
|  | RufiSEAsia | 5 | 0.00097 | 0.00097 | 0.00242 | -1.4275 | 2 | 0 | 2 |  | |  |  |
|  | Nivara | 0 | 0 | 0 | 0 | na | 0 | 0 | 0 |  | |  |  |
|  | Barthii | 1 | 0 | 0 | 0 | 0.85057 | 1 | 0 | 1 |  | |  |  |
|  | Meridionalis | 0 | 0 | 0 | 0 | na | 0 | 0 | 0 |  | |  |  |
| **sts063** |  | # seg sites | pi silent | pi (JC) | theta silent | Taj D | polymor. loci | synonymous | replacement | |  | |  |
|  | SC | 0 | 0 | 0 | 0 | na | 0 | 0 | 0 |  | |  |  |
|  | ARred | 0 | 0 | 0 | 0 | na | 0 | 0 | 0 |  | |  |  |
|  | CAred | 0 | 0 | 0 | 0 | na | 0 | 0 | 0 |  | |  |  |
|  |  |  |  |  |  |  | x | x | x |  | |  |  |
|  | Tempjap | 0 | 0 | 0 | 0 | na | 0 | 0 | 0 |  | |  |  |
|  |  | 0 | 0 | 0 | 0 | na | x | x | x |  | |  |  |
|  |  | 19 | 0.02085 | 0.02114 | 0.02278 | -0.37286 | x | x | x |  | |  |  |
|  | CAcult | 0 | 0 | 0 | 0 | na | 0 | 0 | 0 |  | |  |  |
|  | ARcult | 0 | 0 | 0 | 0 | na | 0 | 0 | 0 |  | |  |  |
|  |  | 2 | 0.00233 | 0.00233 | 0.00254 | -0.24805 | x | x | x |  | |  |  |
|  | indica | 1 | 0.00067 | 0.00067 | 0.00088 | -0.33848 | 0 | 0 | 0 |  | |  |  |
|  | SHWeedy | 0 | 0 | 0 | 0 | na | 0 | 0 | 0 |  | |  |  |
|  | MixWeedy | 0 | 0 | 0 | 0 | na | 0 | 0 | 0 |  | |  |  |
|  | BrHWeedy | 0 | 0 | 0 | 0 | na | 0 | 0 | 0 |  | |  |  |
|  | BHWeedy | 0 | 0 | 0 | 0 | na | 0 | 0 | 0 |  | |  |  |
|  | Rufi | 25 | 0.02764 | 0.02816 |  | 0.08701 |  |  |  |  | |  |  |
|  |  | 0 | 0 | 0 | 0 | na | x | x | x |  | |  |  |
|  | Glumae | 0 | 0 | 0 | 0 | na | 0 | 0 | 0 |  | |  |  |
|  |  | 0 | 0 | 0 | 0 | na | x | x | x |  | |  |  |
|  | glaber | 0 | 0 | 0 | 0 | na | 0 | 0 | 0 |  | |  |  |
|  |  |  |  |  |  |  | x | x | x |  | |  |  |
|  | PA_rice | 0 | 0 | 0 | 0 |  | 0 | 0 | 0 |  | |  |  |
|  |  |  |  |  |  |  |  |  |  |  | |  |  |
|  | Updated Seq Set Analyses | | |  |  |  |  |  |  |  | |  |  |
|  |  |  |  |  |  |  |  |  |  |  | |  |  |
|  |  | # seg sites | pi silent | pi (JC) | theta silent | Taj D |  |  |  |  | |  |  |
|  | CAredWell | 0 | 0 | 0 | 0 | na | 0 | 0 | 0 |  | |  |  |
|  | DemmerWest1 | 0 | 0 | 0 | 0 | na | 0 | 0 | 0 |  | |  |  |
|  | DemmerWest2 | 0 | 0 | 0 | 0 | na | 0 | 0 | 0 |  | |  |  |
|  | DemmerEast | 0 | 0 | 0 | 0 | na | 0 | 0 | 0 |  | |  |  |
|  | TropJap | 0 | 0 | 0 | 0 | na | 0 | 0 | 0 |  | |  |  |
|  | Aromatic | 19 | 0.02085 | 0.02114 | 0.02278 | -0.37286 | 8 | 6 | 2 |  | |  |  |
|  | Aus | 2 | 0.00202 | 0.00203 | 0.00241 | -0.43764 | 0 | 0 | 0 |  | |  |  |
|  | RufiChina | 4 | 0.00131 | 0.00131 | 0.00208 | -1.09607 | 4 | 1 | 3 |  | |  |  |
|  | RufiIndia | 23 | 0.03101 | 0.03167 | 0.01906 | 1.90463 | 10 | 6 | 4 |  | |  |  |
|  | RufiSEAsia | 22 | 0.03303 | 0.03378 |  | 0.61691 | 12 | 6 | 6 |  | |  |  |
|  | Nivara | 0 | 0 | 0 | 0 | na | 0 | 0 | 0 |  | |  |  |
|  | Barthii | 0 | 0 | 0 | 0 | na | 0 | 0 | 0 |  | |  |  |
|  | Meridionalis | 1 | 0.00252 | 0.00252 | 0.00206 | 1.63299 | 0 | 0 | 0 |  | |  |  |
| **sts065** |  | # seg sites | pi silent | pi (JC) | theta silent | Taj D | polymor. loci | synonymous | replacement | |  | |  |
|  | SC | 0 | 0 | 0 | 0 | na | 0 | 0 | 0 |  | |  |  |
|  | ARred | 0 | 0 | 0 | 0 | na | 0 | 0 | 0 |  | |  |  |
|  | CAred | 0 | 0 | 0 | 0 | na | 0 | 0 | 0 |  | |  |  |
|  |  |  |  |  |  |  | x | x | x |  | |  |  |
|  | Tempjap | 0 | 0 | 0 | 0 | na | 0 | 0 | 0 |  | |  |  |
|  |  | 0 | 0 | 0 | 0 | na | x | x | x |  | |  |  |
|  |  | 0 | 0 | 0 | 0 | na | x | x | x |  | |  |  |
|  | CAcult | 0 | 0 | 0 | 0 | na | 0 | 0 | 0 |  | |  |  |
|  | ARcult | 3 | 0.00124 | 0.00124 | 0.00209 | -1.01787 | 0 | 0 | 0 |  | |  |  |
|  |  | 3 | 0.0023 | 0.00231 | 0.00251 | -0.27845 | x | x | x |  | |  |  |
|  | indica | 1 | 0.00024 | 0.00024 | 0.00059 | -0.84519 | 0 | 0 | 0 |  | |  |  |
|  | SHWeedy | 0 | 0 | 0 | 0 | na | 0 | 0 | 0 |  | |  |  |
|  | MixWeedy | 0 | 0 | 0 | 0 | na | 0 | 0 | 0 |  | |  |  |
|  | BrHWeedy | 0 | 0 | 0 | 0 | na | 0 | 0 | 0 |  | |  |  |
|  | BHWeedy | 0 | 0 | 0 | 0 | na | 0 | 0 | 0 |  | |  |  |
|  | Rufi | 16 | 0.00504 | 0.00506 | 0.0101 | -1.41141 |  |  |  |  | |  |  |
|  |  | 0 | 0 | 0 | 0 | na | x | x | x |  | |  |  |
|  | Glumae | 2 | 0.00338 | 0.00339 | 0.00277 | 1.89306 | 0 | 0 | 0 |  | |  |  |
|  |  | 2 | 0.00338 | 0.00338 | 0.00276 | 1.89306 | x | x | x |  | |  |  |
|  | glaber | 2 | 0.00253 | 0.00254 | 0.00195 | 1.10394 | 0 | 0 | 0 |  | |  |  |
|  |  |  |  |  |  |  | x | x | x |  | |  |  |
|  | PA_rice | 0 | 0 | 0 | 0 |  | 0 | 0 | 0 |  | |  |  |
|  |  |  |  |  |  |  |  |  |  |  | |  |  |
|  | Updated Seq Set Analyses | | |  |  |  |  |  |  |  | |  |  |
|  |  |  |  |  |  |  |  |  |  |  | |  |  |
|  |  | # seg sites | pi silent | pi (JC) | theta silent | Taj D |  |  |  |  | |  |  |
|  | CAredWell | 0 | 0 | 0 | 0 | na | 0 | 0 | 0 |  | |  |  |
|  | DemmerWest1 | 0 | 0 | 0 | 0 | na | 0 | 0 | 0 |  | |  |  |
|  | DemmerWest2 | 0 | 0 | 0 | 0 | na | 0 | 0 | 0 |  | |  |  |
|  | DemmerEast | 0 | 0 | 0 | 0 | na | 0 | 0 | 0 |  | |  |  |
|  | TropJap | 0 | 0 | 0 | 0 | na | 0 | 0 | 0 |  | |  |  |
|  | Aromatic | 0 | 0 | 0 | 0 | na | 0 | 0 | 0 |  | |  |  |
|  | Aus | 3 | 0.002 | 0.00201 | 0.00239 | -0.49373 | 0 | 0 | 0 |  | |  |  |
|  | RufiChina | 4 | 0.00173 | 0.00173 | 0.00278 | -1.05777 | 0 | 0 | 0 |  | |  |  |
|  | RufiIndia | 6 | 0.00366 | 0.00367 | 0.00398 | -0.22444 | 0 | 0 | 0 |  | |  |  |
|  | RufiSEAsia | 12 | 0.00543 | 0.00545 | 0.00894 | -1.21395 | 0 | 0 | 0 |  | |  |  |
|  | Nivara | 2 | 0.00269 | 0.0027 | 0.00221 | 1.03194 | 0 | 0 | 0 |  | |  |  |
|  | Barthii | 2 | 0.0027 | 0.00271 | 0.00222 | 1.03194 | 0 | 0 | 0 |  | |  |  |
|  | Meridionalis | |  |  |  |  |  |  |  |  | |  |  |
| **sts066** |  | # seg sites | pi silent | pi (JC) | theta silent | Taj D | #mutations | synonymous | replacement | |  | |  |
|  | SC | 0 | 0 | 0 | 0 | na | 0 | 0 | 0 |  | |  |  |
|  | ARred | 4 | 0.00071 | 0.00071 | 0.00179 | -1.09946 | 0 | 0 | 0 |  | |  |  |
|  | CAred | 0 | 0 | 0 | 0 | na | 0 | 0 | 0 |  | |  |  |
|  |  | 5 | 0.00592 | 0.00594 | 0.00507 | 0.75632 | x | x | x |  | |  |  |
|  | Tempjap | 3 | 0.00072 | 0.00072 | 0.0018 | -1.29256 | 0 | 0 | 0 |  | |  |  |
|  |  | 3 | 0.00083 | 0.00084 | 0.00186 | -1.23707 | x | x | x |  | |  |  |
|  |  | 11 | 0.01234 | 0.01244 | 0.00939 | 1.18113 | x | x | x |  | |  |  |
|  | CAcult | 0 | 0 | 0 | 0 | na | 0 | 0 | 0 |  | |  |  |
|  | ARcult | 0 | 0 | 0 | 0 | na | 0 | 0 | 0 |  | |  |  |
|  |  | 2 | 0.00163 | 0.00164 | 0.00179 | -0.24805 | x | x | x |  | |  |  |
|  | indica | 12 | 0.00548 | 0.0055 | 0.00752 | -0.68617 | 0 | 0 | 0 |  | |  |  |
|  | SHWeedy | 0 | 0 | 0 | 0 | na | 0 | 0 | 0 |  | |  |  |
|  | MixWeedy | 3 | 0.00361 | 0.00362 | 0.00273 | 1.15198 | 0 | 0 | 0 |  | |  |  |
|  | BrHWeedy | 0 | 0 | 0 | 0 | na | 0 | 0 | 0 |  | |  |  |
|  | BHWeedy | 3 | 0.00063 | 0.00063 | 0.00174 | -1.46071 | 0 | 0 | 0 |  | |  |  |
|  | Rufi | 17 | 0.01024 | 0.01031 | 0.0083 | 0.3166 |  |  |  |  | |  |  |
|  |  | 3 | 0.00473 | 0.00474 | 0.00422 | 1.08976 | x | x | x |  | |  |  |
|  | Glumae |  |  |  |  |  |  |  |  |  | |  |  |
|  |  | 11 | 0.0189 | 0.01914 | 0.01546 | 2.24122 | x | x | x |  | |  |  |
|  | glaber | 11 | 0.01215 | 0.01225 | 0.01093 | 0.55962 | 0 | 0 | 0 |  | |  |  |
|  |  | 11 | 0.00445 | 0.00446 | 0.00729 | -1.31172 | x | x | x |  | |  |  |
|  | PA_rice | 3 | 0.00515 | 0.00517 | 0.00422 |  | 0 | 0 | 0 |  | |  |  |
|  |  |  |  |  |  |  |  |  |  |  | |  |  |
|  | Updated Seq Set Analyses | | |  |  |  |  |  |  |  | |  |  |
|  |  |  |  |  |  |  |  |  |  |  | |  |  |
|  |  | # seg sites | pi silent | pi (JC) | theta silent | Taj D |  |  |  |  | |  |  |
|  | CAredWell | 0 | 0 | 0 | 0 | na | 0 | 0 | 0 |  | |  |  |
|  | DemmerWest1 | 0 | 0 | 0 | 0 | na | 0 | 0 | 0 |  | |  |  |
|  | DemmerWest2 | 0 | 0 | 0 | 0 | na | 0 | 0 | 0 |  | |  |  |
|  | DemmerEast | 0 | 0 | 0 | 0 | na | 0 | 0 | 0 |  | |  |  |
|  | TropJap | 3 | 0.00052 | 0.00052 | 0.00167 | -1.37193 | 0 | 0 | 0 |  | |  |  |
|  | Aromatic | 11 | 0.01234 | 0.01244 | 0.00939 | 1.18113 | 0 | 0 | 0 |  | |  |  |
|  | Aus | 2 | 0.00142 | 0.00142 | 0.0017 | -0.43764 | 0 | 0 | 0 |  | |  |  |
|  | RufiChina | 6 | 0.00587 | 0.00589 | 0.00447 | 1.15733 | 0 | 0 | 0 |  | |  |  |
|  | RufiIndia | 12 | 0.00756 | 0.0076 | 0.0081 | -0.38688 | 1 | 1 | 0 |  | |  |  |
|  | RufiSEAsia | 13 | 0.01123 | 0.01132 | 0.00821 | 1.01569 | 0 | 0 | 0 |  | |  |  |
|  | Nivara | 11 | 0.0146 | 0.01475 | 0.01242 | 1.06601 | 0 | 0 | 0 |  | |  |  |
|  | Barthii | 12 | 0.01512 | 0.01527 | 0.01242 | 1.33148 | 1 | 0 | 1 |  | |  |  |
|  | Meridionalis | 1 | 0.00172 | 0.00172 | 0.00141 | 1.63299 | 0 | 0 | 0 |  | |  |  |
| **sts068** |  | # seg sites | pi silent | pi (JC) | theta silent | Taj D | polymor. loci | synonymous | replacement | |  | |  |
|  | SC | 0 | 0 | 0 | 0 | na | 0 | 0 | 0 |  | |  |  |
|  | ARred | 1 | 0.00031 | 0.00031 | 0.00096 | -0.70363 | 0 | 0 | 0 |  | |  |  |
|  | CAred | 0 | 0 | 0 | 0 | na | 0 | 0 | 0 |  | |  |  |
|  |  |  |  |  |  |  | x | x | x |  | |  |  |
|  | Tempjap | 0 | 0 | 0 | 0 | na | 0 | 0 | 0 |  | |  |  |
|  |  | 0 | 0 | 0 | 0 | na | x | x | x |  | |  |  |
|  |  | 0 | 0 | 0 | 0 | na | x | x | x |  | |  |  |
|  | CAcult | 0 | 0 | 0 | 0 | na | 0 | 0 | 0 |  | |  |  |
|  | ARcult | 1 | 0.00088 | 0.00088 | 0.00148 | -0.68111 | 0 | 0 | 0 |  | |  |  |
|  |  | 1 | 0.00268 | 0.00268 | 0.00183 | 1.06589 | x | x | x |  | |  |  |
|  | indica | 2 | 0.00334 | 0.00334 | 0.00257 | 0.55807 | 0 | 0 | 0 |  | |  |  |
|  | SHWeedy | 0 | 0 | 0 | 0 | na | 0 | 0 | 0 |  | |  |  |
|  | MixWeedy | 0 | 0 | 0 | 0 | na | 0 | 0 | 0 |  | |  |  |
|  | BrHWeedy | 0 | 0 | 0 | 0 | na | 0 | 0 | 0 |  | |  |  |
|  | BHWeedy | 0 | 0 | 0 | 0 | na | 0 | 0 | 0 |  | |  |  |
|  | Rufi | 13 | 0.00605 | 0.00608 | 0.01343 | -1.49883 |  |  |  |  | |  |  |
|  |  | 1 | 0.00368 | 0.00369 | 0.00301 | 1.63299 | x | x | x |  | |  |  |
|  | Glumae | 0 | 0 | 0 | 0 | na | 0 | 0 | 0 |  | |  |  |
|  |  | 0 | 0 | 0 | 0 | na | x | x | x |  | |  |  |
|  | glaber | 0 | 0 | 0 | 0 | na | 0 | 0 | 0 |  | |  |  |
|  |  |  |  |  |  |  | x | x | x |  | |  |  |
|  | PA_rice | 1 | 0.00276 | 0.00277 | 0.00301 |  | 0 | 0 | 0 |  | |  |  |
|  |  |  |  |  |  |  |  |  |  |  | |  |  |
|  | Updated Seq Set Analyses | | |  |  |  |  |  |  |  | |  |  |
|  |  |  |  |  |  |  |  |  |  |  | |  |  |
|  |  | # seg sites | pi silent | pi (JC) | theta silent | Taj D |  |  |  |  | |  |  |
|  | CAredWell | 0 | 0 | 0 | 0 | na | 0 | 0 | 0 |  | |  |  |
|  | DemmerWest1 | 0 | 0 | 0 | 0 | na | 0 | 0 | 0 |  | |  |  |
|  | DemmerWest2 | 0 | 0 | 0 | 0 | na | 0 | 0 | 0 |  | |  |  |
|  | DemmerEast | 0 | 0 | 0 | 0 | na | 0 | 0 | 0 |  | |  |  |
|  | TropJap | 0 | 0 | 0 | 0 | na | 0 | 0 | 0 |  | |  |  |
|  | Aromatic | 0 | 0 | 0 | 0 | na | 1 | 1 | 0 |  | |  |  |
|  | Aus | 1 | 0.00291 | 0.00292 | 0.00174 | 1.43413 | 0 | 0 | 0 |  | |  |  |
|  | RufiChina | 1 | 0.00149 | 0.00149 | 0.00165 | -0.17472 | 0 | 0 | 0 |  | |  |  |
|  | RufiIndia | 2 | 0.00297 | 0.00298 | 0.00274 | 0.16869 | 1 | 1 | 0 |  | |  |  |
|  | RufiSEAsia | 7 | 0.00543 | 0.00545 | 0.00899 | -1.08529 | 4 | 4 | 0 |  | |  |  |
|  | Nivara | 7 | 0.01395 | 0.01409 | 0.01273 | 0.83517 | 6 | 3 | 3 |  | |  |  |
|  | Barthii | 0 | 0 | 0 | 0 | na | 0 | 0 | 0 |  | |  |  |
|  | Meridionalis | |  |  |  |  |  |  |  |  | |  |  |
| **sts070** |  | # seg sites | pi silent | pi (JC) | theta silent | Taj D | polymor. loci | synonymous | replacement | |  | |  |
|  | SC | 0 | 0 | 0 | 0 | na | 0 | 0 | 0 |  | |  |  |
|  | ARred | 1 | 0.00038 | 0.00038 | 0.00052 | -0.27467 | 0 | 0 | 0 |  | |  |  |
|  | CAred | 0 | 0 | 0 | 0 | na | 0 | 0 | 0 |  | |  |  |
|  |  |  |  |  |  |  | x | x | x |  | |  |  |
|  | Tempjap | 0 | 0 | 0 | 0 | na | 0 | 0 | 0 |  | |  |  |
|  |  | 0 | 0 | 0 | 0 | na | x | x | x |  | |  |  |
|  |  | 0 | 0 | 0 | 0 | na | x | x | x |  | |  |  |
|  | CAcult | 0 | 0 | 0 | 0 | na | 0 | 0 | 0 |  | |  |  |
|  | ARcult | 0 | 0 | 0 | 0 | na | 0 | 0 | 0 |  | |  |  |
|  |  | 1 | 0.00146 | 0.00146 | 0.00099 | 1.06589 | x | x | x |  | |  |  |
|  | indica | 0 | 0 | 0 | 0 | na | 0 | 0 | 0 |  | |  |  |
|  | SHWeedy | 0 | 0 | 0 | 0 | na | 0 | 0 | 0 |  | |  |  |
|  | MixWeedy | 1 | 0.0016 | 0.0016 | 0.00106 | 1.30268 | 0 | 0 | 0 |  | |  |  |
|  | BrHWeedy | 0 | 0 | 0 | 0 | na | 0 | 0 | 0 |  | |  |  |
|  | BHWeedy | 1 | 0.00085 | 0.00085 | 0.00068 | 0.35171 | 0 | 0 | 0 |  | |  |  |
|  | Rufi | 21 | 0.00403 | 0.00404 | 0.01317 | -2.01372 |  |  |  |  | |  |  |
|  |  | 0 | 0 | 0 | 0 | na | x | x | x |  | |  |  |
|  | Glumae | 0 | 0 | 0 | 0 | na | 0 | 0 | 0 |  | |  |  |
|  |  | 0 | 0 | 0 | 0 | na | x | x | x |  | |  |  |
|  | glaber | 0 | 0 | 0 | 0 | na | 0 | 0 | 0 |  | |  |  |
|  |  |  |  |  |  |  | x | x | x |  | |  |  |
|  | PA_rice | 0 | 0 | 0 | 0 |  | 0 | 0 | 0 |  | |  |  |
|  |  |  |  |  |  |  |  |  |  |  | |  |  |
|  | Updated Seq Set Analyses | | |  |  |  |  |  |  |  | |  |  |
|  |  |  |  |  |  |  |  |  |  |  | |  |  |
|  |  | # seg sites | pi silent | pi (JC) | theta silent | Taj D |  |  |  |  | |  |  |
|  | CAredWell | 0 | 0 | 0 | 0 | na | 0 | 0 | 0 |  | |  |  |
|  | DemmerWest1 | 0 | 0 | 0 | 0 | na | 0 | 0 | 0 |  | |  |  |
|  | DemmerWest2 | 0 | 0 | 0 | 0 | na | 0 | 0 | 0 |  | |  |  |
|  | DemmerEast | 0 | 0 | 0 | 0 | na | 0 | 0 | 0 |  | |  |  |
|  | TropJap | 0 | 0 | 0 | 0 | na | 0 | 0 | 0 |  | |  |  |
|  | Aromatic | 0 | 0 | 0 | 0 | na | 0 | 0 | 0 |  | |  |  |
|  | Aus | 1 | 0.00132 | 0.00132 | 0.00094 | 0.84228 | 0 | 0 | 0 |  | |  |  |
|  | RufiChina | 3 | 0.0022 | 0.0022 | 0.00256 | -0.37679 | 0 | 0 | 0 |  | |  |  |
|  | RufiIndia | 2 | 0.00187 | 0.00187 | 0.0015 | 0.495 | 0 | 0 | 0 |  | |  |  |
|  | RufiSEAsia | 3 | 0.00213 | 0.00213 | 0.00226 | -0.1266 | 0 | 0 | 0 |  | |  |  |
|  | Nivara | 0 | 0 | 0 | 0 | na | 0 | 0 | 0 |  | |  |  |
|  | Barthii | 0 | 0 | 0 | 0 | na | 0 | 0 | 0 |  | |  |  |
|  | Meridionalis | 2 | 0.00402 | 0.00403 | 0.00329 | 1.89306 | 0 | 0 | 0 |  | |  |  |
| **sts071** |  | # seg sites | pi silent | pi (JC) | theta silent | Taj D | polymor. loci | synonymous | replacement | |  | |  |
|  | SC | 0 | 0 | 0 | 0 | na | 0 | 0 | 0 |  | |  |  |
|  | ARred | 14 | 0.0025 | 0.00251 | 0.00809 | -1.76252 | 0 | 0 | 0 |  | |  |  |
|  | CAred | 0 | 0 | 0 | 0 | na | 0 | 0 | 0 |  | |  |  |
|  |  | 0 | 0 | 0 | 0 | na | x | x | x |  | |  |  |
|  | Tempjap | 2 | 0.00062 | 0.00062 | 0.00155 | -1.11882 | 0 | 0 | 0 |  | |  |  |
|  |  | 16 | 0.01632 | 0.0165 | 0.01305 | 0.81532 | x | x | x |  | |  |  |
|  |  | 1 | 0.00118 | 0.00118 | 0.00117 | 0.01889 | x | x | x |  | |  |  |
|  | CAcult | 2 | 0.00339 | 0.0034 | 0.00179 | 0.3329 | 0 | 0 | 0 |  | |  |  |
|  | ARcult | 15 | 0.02488 | 0.0253 | 0.01584 | 2.19019 | 0 | 0 | 0 |  | |  |  |
|  |  | 2 | 0.00201 | 0.00201 | 0.0022 | -0.27845 | x | x | x |  | |  |  |
|  | indica | 0 | 0 | 0 | 0 | na | 0 | 0 | 0 |  | |  |  |
|  | SHWeedy | 0 | 0 | 0 | 0 | na | 0 | 0 | 0 |  | |  |  |
|  | MixWeedy | 1 | 0.00189 | 0.0019 | 0.00128 | 1.79366 | 0 | 0 | 0 |  | |  |  |
|  | BrHWeedy | 0 | 0 | 0 | 0 | na | 0 | 0 | 0 |  | |  |  |
|  | BHWeedy | 16 | 0.0051 | 0.00511 | 0.01219 | -1.66374 | 0 | 0 | 0 |  | |  |  |
|  | Rufi | 24 | 0.0137 | 0.01382 | 0.01762 | -0.58778 |  |  |  |  | |  |  |
|  |  | 0 | 0 | 0 | 0 | na | x | x | x |  | |  |  |
|  | Glumae | 0 | 0 | 0 | 0 | na | 0 | 0 | 0 |  | |  |  |
|  |  | 0 | 0 | 0 | 0 | na | x | x | x |  | |  |  |
|  | glaber | 0 | 0 | 0 | 0 | na | 0 | 0 | 0 |  | |  |  |
|  |  | 16 | 0.02538 | 0.02582 | 0.01366 | 2.68992 | x | x | x |  | |  |  |
|  | PA_rice | 0 | 0 | 0 | 0 |  | 0 | 0 | 0 |  | |  |  |
|  |  |  |  |  |  |  |  |  |  |  | |  |  |
|  | Updated Seq Set Analyses | | |  |  |  |  |  |  |  | |  |  |
|  |  |  |  |  |  |  |  |  |  |  | |  |  |
|  |  | # seg sites | pi silent | pi (JC) | theta silent | Taj D |  |  |  |  | |  |  |
|  | CAredWell | 0 | 0 | 0 | 0 | na | 0 | 0 | 0 |  | |  |  |
|  | DemmerWest1 | 0 | 0 | 0 | 0 | na | 0 | 0 | 0 |  | |  |  |
|  | DemmerWest2 | 0 | 0 | 0 | 0 | na | 0 | 0 | 0 |  | |  |  |
|  | DemmerEast | 0 | 0 | 0 | 0 | na | 0 | 0 | 0 |  | |  |  |
|  | TropJap | 16 | 0.02189 | 0.02222 | 0.0116 | 2.68421 | 0 | 0 | 0 |  | |  |  |
|  | Aromatic | 1 | 0.00118 | 0.00118 | 0.00117 | 0.01889 | 0 | 0 | 0 |  | |  |  |
|  | Aus | 2 | 0.00233 | 0.00234 | 0.00208 | 0.6474 | 0 | 0 | 0 |  | |  |  |
|  | RufiChina | 16 | 0.01263 | 0.01273 | 0.01631 | -0.89527 | 0 | 0 | 0 |  | |  |  |
|  | RufiIndia | 14 | 0.01254 | 0.01264 | 0.01233 | 0.01875 | 0 | 0 | 0 |  | |  |  |
|  | RufiSEAsia | 18 | 0.0074 | 0.00744 | 0.01547 | -1.57005 | 0 | 0 | 0 |  | |  |  |
|  | Nivara | 0 | 0 | 0 | 0 | na | 0 | 0 | 0 |  | |  |  |
|  | Barthii | 0 | 0 | 0 | 0 | na | 0 | 0 | 0 |  | |  |  |
|  | Meridionalis | |  |  |  |  |  |  |  |  | |  |  |
| **sts072** |  | # seg sites | pi silent | pi (JC) | theta silent | Taj D | polymor. loci | synonymous | replacement | |  | |  |
|  | SC | 3 | 0.00512 | 0.00514 | 0.00388 | 1.64797 | 0 | 0 | 0 |  | |  |  |
|  | ARred | 4 | 0.00013 | 0.00013 | 0.00203 | -1.69384 | 0 | 0 | 0 |  | |  |  |
|  | CAred | 0 | 0 | 0 | 0 | na | 0 | 0 | 0 |  | |  |  |
|  |  | 2 | 0.00314 | 0.00315 | 0.00208 | 1.83053 | x | x | x |  | |  |  |
|  | Tempjap | 4 | 0.00114 | 0.00114 | 0.00274 | -1.49001 | 0 | 0 | 0 |  | |  |  |
|  |  | 4 | 0.00351 | 0.00351 | 0.00286 | 0.55775 | x | x | x |  | |  |  |
|  |  | 0 | 0 | 0 | 0 | na | x | x | x |  | |  |  |
|  | CAcult | 5 | 0.00216 | 0.00216 | 0.00383 | -1.23671 | 0 | 0 | 0 |  | |  |  |
|  | ARcult | 4 | 0.00244 | 0.00245 | 0.00339 | -0.84166 | 0 | 0 | 0 |  | |  |  |
|  |  | 0 | 0 | 0 | 0 | na | x | x | x |  | |  |  |
|  | indica | 4 | 0.00588 | 0.00591 | 0.00271 | 2.9369 | 0 | 0 | 0 |  | |  |  |
|  | SHWeedy | 0 | 0 | 0 | 0 | na | 0 | 0 | 0 |  | |  |  |
|  | MixWeedy | 4 | 0.00623 | 0.00625 | 0.00413 | 2.04014 | 0 | 0 | 0 |  | |  |  |
|  | BrHWeedy | 0 | 0 | 0 | 0 | na | 0 | 0 | 0 |  | |  |  |
|  | BHWeedy | 0 | 0 | 0 | 0 | na | 0 | 0 | 0 |  | |  |  |
|  | Rufi | 12 | 0.00675 | 0.00678 | 0.00669 | 0.36001 |  |  |  |  | |  |  |
|  |  | 0 | 0 | 0 | 0 | na | x | x | x |  | |  |  |
|  | Glumae | 0 | 0 | 0 | 0 | na | 0 | 0 | 0 |  | |  |  |
|  |  | 0 | 0 | 0 | 0 | na | x | x | x |  | |  |  |
|  | glaber | 0 | 0 | 0 | 0 | na | 0 | 0 | 0 |  | |  |  |
|  |  | 4 | 0.00182 | 0.00183 | 0.00295 | -1.09827 | x | x | x |  | |  |  |
|  | PA_rice | 0 | 0 | 0 | 0 |  | 0 | 0 | 0 |  | |  |  |
|  |  |  |  |  |  |  |  |  |  |  | |  |  |
|  | Updated Seq Set Analyses | | |  |  |  |  |  |  |  | |  |  |
|  |  |  |  |  |  |  |  |  |  |  | |  |  |
|  |  | # seg sites | pi silent | pi (JC) | theta silent | Taj D |  |  |  |  | |  |  |
|  | CAredWell | 0 | 0 | 0 | 0 | na | 0 | 0 | 0 |  | |  |  |
|  | DemmerWest1 | 0 | 0 | 0 | 0 | na | 0 | 0 | 0 |  | |  |  |
|  | DemmerWest2 | 0 | 0 | 0 | 0 | na | 0 | 0 | 0 |  | |  |  |
|  | DemmerEast | 0 | 0 | 0 | 0 | na | 0 | 0 | 0 |  | |  |  |
|  | TropJap | 4 | 0.00221 | 0.00222 | 0.00253 | -0.27761 | 0 | 0 | 0 |  | |  |  |
|  | Aromatic | 0 | 0 | 0 | 0 | na | 0 | 0 | 0 |  | |  |  |
|  | Aus | 0 | 0 | 0 | 0 | na | 0 | 0 | 0 |  | |  |  |
|  | RufiChina | 5 | 0.00226 | 0.00226 | 0.004 | -1.31397 | 0 | 0 | 0 |  | |  |  |
|  | RufiIndia | 7 | 0.00656 | 0.00659 | 0.00507 | 1.09579 | 0 | 0 | 0 |  | |  |  |
|  | RufiSEAsia | 8 | 0.00663 | 0.00666 | 0.00543 | 0.9548 | 0 | 0 | 0 |  | |  |  |
|  | Nivara | 3 | 0.00467 | 0.00468 | 0.00383 | 1.18059 | 0 | 0 | 0 |  | |  |  |
|  | Barthii | 0 | 0 | 0 | 0 | na | 0 | 0 | 0 |  | |  |  |
|  | Meridionalis | 0 | 0 | 0 | 0 | na | 0 | 0 | 0 |  | |  |  |
| **sts073** |  | # seg sites | pi silent | pi (JC) | theta silent | Taj D | polymor. loci | synonymous | replacement | |  | |  |
|  | SC | 0 | 0 | 0 | 0 | na | 0 | 0 | 0 |  | |  |  |
|  | ARred | 0 | 0 | 0 | 0 | na | 0 | 0 | 0 |  | |  |  |
|  | CAred | 0 | 0 | 0 | 0 | na | 0 | 0 | 0 |  | |  |  |
|  |  |  |  |  |  |  | x | x | x |  | |  |  |
|  | Tempjap | 3 | 0.0047 | 0.00471 | 0.00345 | 0.77388 | 0 | 0 | 0 |  | |  |  |
|  |  | 3 | 0.00195 | 0.00195 | 0.00239 | -0.68677 | x | x | x |  | |  |  |
|  |  | 3 | 0.00721 | 0.00724 | 0.00492 | 1.52266 | x | x | x |  | |  |  |
|  | CAcult | 3 | 0.00634 | 0.00637 | 0.00419 | 1.33196 | 0 | 0 | 0 |  | |  |  |
|  | ARcult | 0 | 0 | 0 | 0 | na | 0 | 0 | 0 |  | |  |  |
|  |  | 3 | 0.0045 | 0.00452 | 0.00492 | -0.27845 | x | x | x |  | |  |  |
|  | indica | 0 | 0 | 0 | 0 | na | 0 | 0 | 0 |  | |  |  |
|  | SHWeedy | 0 | 0 | 0 | 0 | na | 0 | 0 | 0 |  | |  |  |
|  | MixWeedy | 0 | 0 | 0 | 0 | na | 0 | 0 | 0 |  | |  |  |
|  | BrHWeedy | 0 | 0 | 0 | 0 | na | 0 | 0 | 0 |  | |  |  |
|  | BHWeedy | 0 | 0 | 0 | 0 | na | 0 | 0 | 0 |  | |  |  |
|  | Rufi | 7 | 0.00804 | 0.00809 | 0.00662 | 0.18021 |  |  |  |  | |  |  |
|  |  | 2 | 0.00712 | 0.00715 | 0.00776 | -0.7099 | x | x | x |  | |  |  |
|  | Glumae | 1 | 0.0033 | 0.00331 | 0.0027 | 1.63299 | 0 | 0 | 0 |  | |  |  |
|  |  | 0 | 0 | 0 | 0 | na | x | x | x |  | |  |  |
|  | glaber | 0 | 0 | 0 | 0 | na | 0 | 0 | 0 |  | |  |  |
|  |  |  |  |  |  |  | x | x | x |  | |  |  |
|  | PA_rice |  |  |  |  |  |  |  |  |  | |  |  |
|  |  |  |  |  |  |  |  |  |  |  | |  |  |
|  | Updated Seq Set Analyses | | |  |  |  |  |  |  |  | |  |  |
|  |  |  |  |  |  |  |  |  |  |  | |  |  |
|  |  | # seg sites | pi silent | pi (JC) | theta silent | Taj D |  |  |  |  | |  |  |
|  | CAredWell | 0 | 0 | 0 | 0 | na | 0 | 0 | 0 |  | |  |  |
|  | DemmerWest1 | 0 | 0 | 0 | 0 | na | 0 | 0 | 0 |  | |  |  |
|  | DemmerWest2 | 0 | 0 | 0 | 0 | na | 0 | 0 | 0 |  | |  |  |
|  | DemmerEast | 0 | 0 | 0 | 0 | na | 0 | 0 | 0 |  | |  |  |
|  | TropJap | 3 | 0.00179 | 0.00179 | 0.00212 | -0.66688 | 1 | 0 | 1 |  | |  |  |
|  | Aromatic | 3 | 0.00721 | 0.00724 | 0.00492 | 1.52266 | 0 | 0 | 0 |  | |  |  |
|  | Aus | 3 | 0.00392 | 0.00393 | 0.00467 | -0.49373 | 0 | 0 | 0 |  | |  |  |
|  | RufiChina | 3 | 0.00547 | 0.00549 | 0.00408 | 0.88296 | 0 | 0 | 0 |  | |  |  |
|  | RufiIndia | 3 | 0.00467 | 0.00469 | 0.00369 | 0.61627 | 0 | 0 | 0 |  | |  |  |
|  | RufiSEAsia | 5 | 0.00781 | 0.00786 | 0.00461 | 1.00248 | 1 | 0 | 1 |  | |  |  |
|  | Nivara | 2 | 0.00474 | 0.00476 | 0.00623 | -1.13197 | 0 | 0 | 0 |  | |  |  |
|  | Barthii | 0 | 0 | 0 | 0 | na | 0 | 0 | 0 |  | |  |  |
|  | Meridionalis | 0 | 0 | 0 | 0 | na | 0 | 0 | 0 |  | |  |  |
| **sts080** |  | # seg sites | pi silent | pi (JC) | theta silent | Taj D | polymor. loci | synonymous | replacement | |  | |  |
|  | SC | 0 | 0 | 0 | 0 | na | 0 | 0 | 0 |  | |  |  |
|  | ARred | 3 | 0.00083 | 0.00083 | 0.00225 | -1.03184 | 0 | 0 | 0 |  | |  |  |
|  | CAred | 0 | 0 | 0 | 0 | na | 0 | 0 | 0 |  | |  |  |
|  |  |  |  |  |  |  | x | x | x |  | |  |  |
|  | Tempjap | 0 | 0 | 0 | 0 | na | 0 | 0 | 0 |  | |  |  |
|  |  | 0 | 0 | 0 | 0 | na | x | x | x |  | |  |  |
|  |  | 0 | 0 | 0 | 0 | na | x | x | x |  | |  |  |
|  | CAcult | 0 | 0 | 0 | 0 | na | 0 | 0 | 0 |  | |  |  |
|  | ARcult | 1 | 0.00069 | 0.00069 | 0.00115 | -0.68111 | 0 | 0 | 0 |  | |  |  |
|  |  | 2 | 0.00444 | 0.00445 | 0.00285 | 1.62381 | x | x | x |  | |  |  |
|  | indica | 2 | 0.0008 | 0.0008 | 0.002 | -1.11882 | 0 | 0 | 0 |  | |  |  |
|  | SHWeedy | 0 | 0 | 0 | 0 | na | 0 | 0 | 0 |  | |  |  |
|  | MixWeedy | 2 | 0.00307 | 0.00307 | 0.00305 | 0.01889 | 0 | 0 | 0 |  | |  |  |
|  | BrHWeedy | 0 | 0 | 0 | 0 | na | 0 | 0 | 0 |  | |  |  |
|  | BHWeedy | 1 | 0.0015 | 0.0015 | 0.00098 | 0.72613 | 0 | 0 | 0 |  | |  |  |
|  | Rufi | 11 | 0.00411 | 0.00412 | 0.00741 | -1.42865 |  |  |  |  | |  |  |
|  |  | 0 | 0 | 0 | 0 | na | x | x | x |  | |  |  |
|  | Glumae | 0 | 0 | 0 | 0 | na | 0 | 0 | 0 |  | |  |  |
|  |  | 0 | 0 | 0 | 0 | na | x | x | x |  | |  |  |
|  | glaber | 0 | 0 | 0 | 0 | na | 0 | 0 | 0 |  | |  |  |
|  |  |  |  |  |  |  | x | x | x |  | |  |  |
|  | PA_rice | 2 | 0.00575 | 0.00577 | 0.0047 |  | 0 | 0 | 0 |  | |  |  |
|  |  |  |  |  |  |  |  |  |  |  | |  |  |
|  | Updated Seq Set Analyses | | |  |  |  |  |  |  |  | |  |  |
|  |  |  |  |  |  |  |  |  |  |  | |  |  |
|  |  | # seg sites | pi silent | pi (JC) | theta silent | Taj D |  |  |  |  | |  |  |
|  | CAredWell | 0 | 0 | 0 | 0 | na | 0 | 0 | 0 |  | |  |  |
|  | DemmerWest1 | 0 | 0 | 0 | 0 | na | 0 | 0 | 0 |  | |  |  |
|  | DemmerWest2 | 0 | 0 | 0 | 0 | na | 0 | 0 | 0 |  | |  |  |
|  | DemmerEast | 0 | 0 | 0 | 0 | na | 0 | 0 | 0 |  | |  |  |
|  | TropJap | 0 | 0 | 0 | 0 | na | 0 | 0 | 0 |  | |  |  |
|  | Aromatic | 0 | 0 | 0 | 0 | na | 0 | 0 | 0 |  | |  |  |
|  | Aus | 2 | 0.00417 | 0.00418 | 0.00271 | 1.45891 | 0 | 0 | 0 |  | |  |  |
|  | RufiChina | 2 | 0.00258 | 0.00258 | 0.00236 | 0.20135 | 0 | 0 | 0 |  | |  |  |
|  | RufiIndia | 3 | 0.00207 | 0.00207 | 0.00214 | -0.7001 | 2 | 1 | 1 |  | |  |  |
|  | RufiSEAsia | 6 | 0.00507 | 0.00509 | 0.00701 | -0.96452 | 2 | 2 | 0 |  | |  |  |
|  | Nivara | 2 | 0.0046 | 0.00461 | 0.00378 | 1.03194 | 0 | 0 | 0 |  | |  |  |
|  | Barthii | 1 | 0 | 0 | 0 | 0.85057 | 1 | 0 | 1 |  | |  |  |
|  | Meridionalis | 1 | 0.00287 | 0.00288 | 0.00235 | 1.63299 | 0 | 0 | 0 |  | |  |  |
| **sts082** |  | # seg sites | pi silent | pi (JC) | theta silent | Taj D | polymor. loci | synonymous | replacement | |  | |  |
|  | SC | 0 | 0 | 0 | 0 | na | 0 | 0 | 0 |  | |  |  |
|  | ARred | 0 | 0 | 0 | 0 | na | 0 | 0 | 0 |  | |  |  |
|  | CAred | 0 | 0 | 0 | 0 | na | 0 | 0 | 0 |  | |  |  |
|  |  |  |  |  |  |  | x | x | x |  | |  |  |
|  | Tempjap | 0 | 0 | 0 | 0 | na | 0 | 0 | 0 |  | |  |  |
|  |  | 0 | 0 | 0 | 0 | na | x | x | x |  | |  |  |
|  |  | 0 | 0 | 0 | 0 | na | x | x | x |  | |  |  |
|  | CAcult | 0 | 0 | 0 | 0 | na | 0 | 0 | 0 |  | |  |  |
|  | ARcult | 0 | 0 | 0 | 0 | na | 0 | 0 | 0 |  | |  |  |
|  |  | 0 | 0 | 0 | 0 | na | x | x | x |  | |  |  |
|  | indica | 0 | 0 | 0 | 0 | na | 0 | 0 | 0 |  | |  |  |
|  | SHWeedy | 0 | 0 | 0 | 0 | na | 0 | 0 | 0 |  | |  |  |
|  | MixWeedy | 0 | 0 | 0 | 0 | na | 0 | 0 | 0 |  | |  |  |
|  | BrHWeedy | 0 | 0 | 0 | 0 | na | 0 | 0 | 0 |  | |  |  |
|  | BHWeedy | 0 | 0 | 0 | 0 | na | 0 | 0 | 0 |  | |  |  |
|  | Rufi | 16 | 0.00327 | 0.00328 | 0.01226 | -2.12747 |  |  |  |  | |  |  |
|  |  | 0 | 0 | 0 | 0 | na | x | x | x |  | |  |  |
|  | Glumae | 1 | 0.00238 | 0.00238 | 0.00195 | 1.63299 | 0 | 0 | 0 |  | |  |  |
|  |  | 1 | 0.00238 | 0.00238 | 0.00195 | 1.63299 | x | x | x |  | |  |  |
|  | glaber | 0 | 0 | 0 | 0 | na | 0 | 0 | 0 |  | |  |  |
|  |  |  |  |  |  |  | x | x | x |  | |  |  |
|  | PA_rice | 0 | 0 | 0 | 0 |  | 0 | 0 | 0 |  | |  |  |
|  |  |  |  |  |  |  |  |  |  |  | |  |  |
|  | Updated Seq Set Analyses | | |  |  |  |  |  |  |  | |  |  |
|  |  |  |  |  |  |  |  |  |  |  | |  |  |
|  |  | # seg sites | pi silent | pi (JC) | theta silent | Taj D |  |  |  |  | |  |  |
|  | CAredWell | 0 | 0 | 0 | 0 | na | 0 | 0 | 0 |  | |  |  |
|  | DemmerWest1 | 0 | 0 | 0 | 0 | na | 0 | 0 | 0 |  | |  |  |
|  | DemmerWest2 | 0 | 0 | 0 | 0 | na | 0 | 0 | 0 |  | |  |  |
|  | DemmerEast | 0 | 0 | 0 | 0 | na | 0 | 0 | 0 |  | |  |  |
|  | TropJap | 0 | 0 | 0 | 0 | na | 0 | 0 | 0 |  | |  |  |
|  | Aromatic | 0 | 0 | 0 | 0 | na | 0 | 0 | 0 |  | |  |  |
|  | Aus | 0 | 0 | 0 | 0 | na | 0 | 0 | 0 |  | |  |  |
|  | RufiChina | 0 | 0 | 0 | 0 | na | 0 | 0 | 0 |  | |  |  |
|  | RufiIndia | 3 | 0.00242 | 0.00242 | 0.00266 | -0.20946 | 1 | 1 | 0 |  | |  |  |
|  | RufiSEAsia | 3 | 0.00128 | 0.00129 | 0.00249 | -1.04143 | 1 | 1 | 0 |  | |  |  |
|  | Nivara | 0 | 0 | 0 | 0 | na | 0 | 0 | 0 |  | |  |  |
|  | Barthii | 1 | 0.0019 | 0.0019 | 0.00156 | 0.85057 | 0 | 0 | 0 |  | |  |  |
|  | Meridionalis | 0 | 0 | 0 | 0 | na | 0 | 0 | 0 |  | |  |  |
| **sts083** |  | # seg sites | pi silent | pi (JC) | theta silent | Taj D | polymor. loci | synonymous | replacement | |  | |  |
|  | SC | 0 | 0 | 0 | 0 | na | 0 | 0 | 0 |  | |  |  |
|  | ARred | 8 | 0.00142 | 0.00142 | 0.00548 | -1.66291 | 1 | 1 | 0 |  | |  |  |
|  | CAred | 0 | 0 | 0 | 0 | na | 0 | 0 | 0 |  | |  |  |
|  |  |  |  |  |  |  | x | x | x |  | |  |  |
|  | Tempjap | 0 | 0 | 0 | 0 | na | 1 | 1 | 0 |  | |  |  |
|  |  | 0 | 0 | 0 | 0 | na | x | x | x |  | |  |  |
|  |  | 7 | 0.00847 | 0.00852 | 0.00925 | -0.33897 | x | x | x |  | |  |  |
|  | CAcult | 0 | 0 | 0 | 0 | na | 1 | 1 | 0 |  | |  |  |
|  | ARcult | 9 | 0.01025 | 0.01032 | 0.00947 | 0.27009 | 1 | 1 | 0 |  | |  |  |
|  |  | 0 | 0 | 0 | 0 | na | x | x | x |  | |  |  |
|  | indica | 0 | 0 | 0 | 0 | na | 1 | 1 | 0 |  | |  |  |
|  | SHWeedy | 0 | 0 | 0 | 0 | na | 0 | 0 | 0 |  | |  |  |
|  | MixWeedy | 7 | 0.01484 | 0.01499 | 0.00984 | 2.19508 | 1 | 1 | 0 |  | |  |  |
|  | BrHWeedy | 0 | 0 | 0 | 0 | na | 0 | 0 | 0 |  | |  |  |
|  | BHWeedy | 0 | 0 | 0 | 0 | na | 1 | 1 | 0 |  | |  |  |
|  | Rufi | 9 | 0.01241 | 0.01251 | 0.00963 | 0.46043 |  |  |  |  | |  |  |
|  |  | 9 | 0.02376 | 0.02415 | 0.01944 | 2.21181 | x | x | x |  | |  |  |
|  | Glumae | 0 | 0 | 0 | 0 | na | 0 | 0 | 0 |  | |  |  |
|  |  | 0 | 0 | 0 | 0 | na | x | x | x |  | |  |  |
|  | glaber | 0 | 0 | 0 | 0 | na | 0 | 0 | 0 |  | |  |  |
|  |  |  |  |  |  |  | x | x | x |  | |  |  |
|  | PA_rice | 8 | 0.02096 | 0.02125 | 0.01715 |  | 0 | 0 | 0 |  | |  |  |
|  |  |  |  |  |  |  |  |  |  |  | |  |  |
|  | Updated Seq Set Analyses | | |  |  |  |  |  |  |  | |  |  |
|  |  |  |  |  |  |  |  |  |  |  | |  |  |
|  |  | # seg sites | pi silent | pi (JC) | theta silent | Taj D |  |  |  |  | |  |  |
|  | CAredWell | 0 | 0 | 0 | 0 | na | 0 | 0 | 0 |  | |  |  |
|  | DemmerWest1 | 0 | 0 | 0 | 0 | na | 0 | 0 | 0 |  | |  |  |
|  | DemmerWest2 | 0 | 0 | 0 | 0 | na | 0 | 0 | 0 |  | |  |  |
|  | DemmerEast | 0 | 0 | 0 | 0 | na | 0 | 0 | 0 |  | |  |  |
|  | TropJap | 1 | 0.00028 | 0.00028 | 0.0009 | -1.18693 | 2 | 2 | 0 |  | |  |  |
|  | Aromatic | 7 | 0.00907 | 0.00913 | 0.00991 | -0.33897 | 4 | 4 | 0 |  | |  |  |
|  | Aus | 0 | 0 | 0 | 0 | na | 0 | 0 | 0 |  | |  |  |
|  | RufiChina | 8 | 0.0136 | 0.01373 | 0.0096 | 1.49119 | 4 | 4 | 0 |  | |  |  |
|  | RufiIndia | 6 | 0.01039 | 0.01046 | 0.00919 | 0.37955 | 5 | 5 | 0 |  | |  |  |
|  | RufiSEAsia | 10 | 0.01052 | 0.01059 | 0.00998 | 0.08187 | 4 | 4 | 0 |  | |  |  |
|  | Nivara | 9 | 0.02035 | 0.02063 | 0.01671 | 1.30798 | 2 | 2 | 0 |  | |  |  |
|  | Barthii | 0 | 0 | 0 | 0 | na | 0 | 0 | 0 |  | |  |  |
|  | Meridionalis | 0 | 0 | 0 | 0 | na | 0 | 0 | 0 |  | |  |  |
| **sts085** |  | # seg sites | pi silent | pi (JC) | theta silent | Taj D | polymor. loci | synonymous | replacement | |  | |  |
|  | SC | 0 | 0 | 0 | 0 | na | 0 | 0 | 0 |  | |  |  |
|  | ARred | 2 | 0.00025 | 0.00025 | 0.00078 | -0.94875 | 1 | 1 | 0 |  | |  |  |
|  | CAred | 4 | 0.00065 | 0.00065 | 0.00196 | -1.49249 | 0 | 0 | 0 |  | |  |  |
|  |  |  |  |  |  |  | x | x | x |  | |  |  |
|  | Tempjap | 5 | 0.00214 | 0.00214 | 0.00261 | -0.43011 | 1 | 1 | 0 |  | |  |  |
|  |  | 8 | 0.00609 | 0.00612 | 0.00431 | 1.33816 | x | x | x |  | |  |  |
|  |  | 2 | 0.00159 | 0.00159 | 0.00158 | 0.01889 | x | x | x |  | |  |  |
|  | CAcult | 5 | 0.00352 | 0.00353 | 0.0031 | 0.40487 | 1 | 1 | 0 |  | |  |  |
|  | ARcult | 5 | 0.00415 | 0.00416 | 0.003 | 1.11708 | 1 | 1 | 0 |  | |  |  |
|  |  | 2 | 0.00135 | 0.00135 | 0.00148 | -0.24805 | x | x | x |  | |  |  |
|  | indica | 4 | 0.00207 | 0.00208 | 0.00207 | -0.00113 | 1 | 1 | 0 |  | |  |  |
|  | SHWeedy | 0 | 0 | 0 | 0 | na | 0 | 0 | 0 |  | |  |  |
|  | MixWeedy | 3 | 0.00203 | 0.00204 | 0.00237 | -0.50669 | 1 | 1 | 0 |  | |  |  |
|  | BrHWeedy | 0 | 0 | 0 | 0 | na | 0 | 0 | 0 |  | |  |  |
|  | BHWeedy | 3 | 0.00277 | 0.00278 | 0.00151 | 1.07033 | 1 | 1 | 0 |  | |  |  |
|  | Rufi | 32 | 0.00804 | 0.00808 | 0.0142 | -1.49313 |  |  |  |  | |  |  |
|  |  | 5 | 0.00744 | 0.00748 | 0.00609 | 2.12492 | x | x | x |  | |  |  |
|  | Glumae | 4 | 0.00595 | 0.00598 | 0.00487 | 2.12492 | 0 | 0 | 0 |  | |  |  |
|  |  | 0 | 0 | 0 | 0 | na | x | x | x |  | |  |  |
|  | glaber | 0 | 0 | 0 | 0 | na | 0 | 0 | 0 |  | |  |  |
|  |  |  |  |  |  |  | x | x | x |  | |  |  |
|  | PA_rice | 0 | 0 | 0 | 0 |  | 0 | 0 | 0 |  | |  |  |
|  |  |  |  |  |  |  |  |  |  |  | |  |  |
|  | Updated Seq Set Analyses | | |  |  |  |  |  |  |  | |  |  |
|  |  |  |  |  |  |  |  |  |  |  | |  |  |
|  |  | # seg sites | pi silent | pi (JC) | theta silent | Taj D |  |  |  |  | |  |  |
|  | CAredWell | 4 | 0.00208 | 0.00209 | 0.00269 | -0.70625 | 0 | 0 | 0 |  | |  |  |
|  | DemmerWest1 | 0 | 0 | 0 | 0 | na | 0 | 0 | 0 |  | |  |  |
|  | DemmerWest2 | 0 | 0 | 0 | 0 | na | 0 | 0 | 0 |  | |  |  |
|  | DemmerEast | 0 | 0 | 0 | 0 | na | 0 | 0 | 0 |  | |  |  |
|  | TropJap | 8 | 0.00595 | 0.00597 | 0.00383 | 1.63026 | 2 | 2 | 0 |  | |  |  |
|  | Aromatic | 2 | 0.00159 | 0.00159 | 0.00158 | 0.01889 | 4 | 4 | 0 |  | |  |  |
|  | Aus | 2 | 0.00118 | 0.00118 | 0.0014 | -0.43764 | 0 | 0 | 0 |  | |  |  |
|  | RufiChina | 10 | 0.00677 | 0.0068 | 0.00614 | 0.49493 | 4 | 4 | 0 |  | |  |  |
|  | RufiIndia | 12 | 0.00592 | 0.00594 | 0.00665 | -0.40519 | 5 | 5 | 0 |  | |  |  |
|  | RufiSEAsia | 23 | 0.00833 | 0.00838 | 0.01268 | -1.32468 | 4 | 4 | 0 |  | |  |  |
|  | Nivara | 9 | 0.01071 | 0.01079 | 0.0088 | 1.2971 | 2 | 2 | 0 |  | |  |  |
|  | Barthii | 0 | 0 | 0 | 0 | na | 0 | 0 | 0 |  | |  |  |
|  | Meridionalis | |  |  |  |  | 0 | 0 | 0 |  | |  |  |
| **sts086** |  | # seg sites | pi silent | pi (JC) | theta silent | Taj D | polymor. loci | synonymous | replacement | |  | |  |
|  | SC | 0 | 0 | 0 | 0 | na | 0 | 0 | 0 |  | |  |  |
|  | ARred | 2 | 0.00008 | 0.00008 | 0.0013 | -1.30213 | 1 | 1 | 0 |  | |  |  |
|  | CAred | 2 | 0.00054 | 0.00054 | 0.00164 | -1.17143 | 2 | 2 | 0 |  | |  |  |
|  |  | 2 | 0.00268 | 0.00268 | 0.0024 | -0.07082 | x | x | x |  | |  |  |
|  | Tempjap | 2 | 0.00187 | 0.00187 | 0.00173 | 0.14816 | 1 | 1 | 0 |  | |  |  |
|  |  | 2 | 0.0008 | 0.0008 | 0.0018 | -1.23707 | x | x | x |  | |  |  |
|  |  | 8 | 0.01126 | 0.01134 | 0.01053 | 0.24887 | x | x | x |  | |  |  |
|  | CAcult | 2 | 0.00129 | 0.00129 | 0.00205 | -0.83549 | 1 | 1 | 0 |  | |  |  |
|  | ARcult | 2 | 0.00216 | 0.00216 | 0.002 | 0.1812 | 1 | 1 | 0 |  | |  |  |
|  |  | 0 | 0 | 0 | 0 | na | x | x | x |  | |  |  |
|  | indica | 2 | 0.00132 | 0.00132 | 0.00173 | -0.44806 | 1 | 1 | 0 |  | |  |  |
|  | SHWeedy | 0 | 0 | 0 | 0 | na | 0 | 0 | 0 |  | |  |  |
|  | MixWeedy | 2 | 0.00397 | 0.00398 | 0.00263 | 1.64145 | 1 | 1 | 0 |  | |  |  |
|  | BrHWeedy | 0 | 0 | 0 | 0 | na | 0 | 0 | 0 |  | |  |  |
|  | BHWeedy | 2 | 0.00061 | 0.00061 | 0.00169 | -1.14975 | 1 | 1 | 0 |  | |  |  |
|  | Rufi | 18 | 0.01307 | 0.01319 | 0.0128 | 0.10276 |  |  |  |  | |  |  |
|  |  | 2 | 0.00497 | 0.00498 | 0.00406 | 1.89306 | x | x | x |  | |  |  |
|  | Glumae | 0 | 0 | 0 | 0 | na | 0 | 0 | 0 |  | |  |  |
|  |  | 0 | 0 | 0 | 0 | na | x | x | x |  | |  |  |
|  | glaber | 0 | 0 | 0 | 0 | na | 0 | 0 | 0 |  | |  |  |
|  |  | 3 | 0.00295 | 0.00295 | 0.00282 | 0.10425 | x | x | x |  | |  |  |
|  | PA_rice | 0 | 0 | 0 | 0 |  | 0 | 0 | 0 |  | |  |  |
|  |  |  |  |  |  |  |  |  |  |  | |  |  |
|  | Updated Seq Set Analyses | | |  |  |  |  |  |  |  | |  |  |
|  |  |  |  |  |  |  |  |  |  |  | |  |  |
|  |  | # seg sites | pi silent | pi (JC) | theta silent | Taj D |  |  |  |  | |  |  |
|  | CAredWell | 0 | 0 | 0 | 0 | na | 0 | 0 | 0 |  | |  |  |
|  | DemmerWest1 | 0 | 0 | 0 | 0 | na | 0 | 0 | 0 |  | |  |  |
|  | DemmerWest2 | 0 | 0 | 0 | 0 | na | 0 | 0 | 0 |  | |  |  |
|  | DemmerEast | 2 | 0.00156 | 0.00156 | 0.00217 | -0.68482 | 2 | 2 | 0 |  | |  |  |
|  | TropJap | 2 | 0.00049 | 0.00049 | 0.0016 | -1.37785 | 2 | 2 | 0 |  | |  |  |
|  | Aromatic | 8 | 0.01126 | 0.01134 | 0.01053 | 0.24887 | 4 | 4 | 0 |  | |  |  |
|  | Aus | 0 | 0 | 0 | 0 | na | 0 | 0 | 0 |  | |  |  |
|  | RufiChina | 8 | 0.00771 | 0.00775 | 0.00817 | -0.3158 | 4 | 4 | 0 |  | |  |  |
|  | RufiIndia | 12 | 0.01331 | 0.01343 | 0.0111 | 0.79653 | 5 | 5 | 0 |  | |  |  |
|  | RufiSEAsia | 14 | 0.01377 | 0.0139 | 0.01212 | 0.38949 | 4 | 4 | 0 |  | |  |  |
|  | Nivara | 3 | 0.00596 | 0.00598 | 0.00489 | 1.12414 | 2 | 2 | 0 |  | |  |  |
|  | Barthii | 0 | 0 | 0 | 0 | na | 0 | 0 | 0 |  | |  |  |
|  | Meridionalis | 2 | 0.00497 | 0.00498 | 0.00406 | 1.89306 | 0 | 0 | 0 |  | |  |  |
| **sts087** |  | # seg sites | pi silent | pi (JC) | theta silent | Taj D | polymor. loci | synonymous | replacement | |  | |  |
|  | SC | 0 | 0 | 0 | 0 | na | 0 | 0 | 0 |  | |  |  |
|  | ARred | 0 | 0 | 0 | 0 | na | 0 | 0 | 0 |  | |  |  |
|  | CAred | 0 | 0 | 0 | 0 | na | 0 | 0 | 0 |  | |  |  |
|  |  |  |  |  |  |  | x | x | x |  | |  |  |
|  | Tempjap | 0 | 0 | 0 | 0 | na | 0 | 0 | 0 |  | |  |  |
|  |  | 0 | 0 | 0 | 0 | na | x | x | x |  | |  |  |
|  |  | 0 | 0 | 0 | 0 | na | x | x | x |  | |  |  |
|  | CAcult | 0 | 0 | 0 | 0 | na | 0 | 0 | 0 |  | |  |  |
|  | ARcult | 0 | 0 | 0 | 0 | na | 0 | 0 | 0 |  | |  |  |
|  |  | 0 | 0 | 0 | 0 | na | x | x | x |  | |  |  |
|  | indica | 0 | 0 | 0 | 0 | na | 0 | 0 | 0 |  | |  |  |
|  | SHWeedy | 0 | 0 | 0 | 0 | na | 0 | 0 | 0 |  | |  |  |
|  | MixWeedy | 0 | 0 | 0 | 0 | na | 0 | 0 | 0 |  | |  |  |
|  | BrHWeedy | 0 | 0 | 0 | 0 | na | 0 | 0 | 0 |  | |  |  |
|  | BHWeedy | 0 | 0 | 0 | 0 | na | 0 | 0 | 0 |  | |  |  |
|  | Rufi | 9 | 0.00386 | 0.00387 | 0.01158 | -1.71704 |  |  |  |  | |  |  |
|  |  | 0 | 0 | 0 | 0 | na | x | x | x |  | |  |  |
|  | Glumae | 2 | 0.00404 | 0.00405 | 0.00331 | 1.89306 | 1 | 0 | 1 |  | |  |  |
|  |  | 0 | 0 | 0 | 0 | na | x | x | x |  | |  |  |
|  | glaber | 0 | 0 | 0 | 0 | na | 0 | 0 | 0 |  | |  |  |
|  |  |  |  |  |  |  | x | x | x |  | |  |  |
|  | PA_rice | 0 | 0 | 0 | 0 |  | 0 | 0 | 0 |  | |  |  |
|  |  |  |  |  |  |  |  |  |  |  | |  |  |
|  | Updated Seq Set Analyses | | |  |  |  |  |  |  |  | |  |  |
|  |  |  |  |  |  |  |  |  |  |  | |  |  |
|  |  | # seg sites | pi silent | pi (JC) | theta silent | Taj D |  |  |  |  | |  |  |
|  | CAredWell | 0 | 0 | 0 | 0 | na | 0 | 0 | 0 |  | |  |  |
|  | DemmerWest1 | 0 | 0 | 0 | 0 | na | 0 | 0 | 0 |  | |  |  |
|  | DemmerWest2 | 0 | 0 | 0 | 0 | na | 0 | 0 | 0 |  | |  |  |
|  | DemmerEast | 0 | 0 | 0 | 0 | na | 0 | 0 | 0 |  | |  |  |
|  | TropJap | 0 | 0 | 0 | 0 | na | 0 | 0 | 0 |  | |  |  |
|  | Aromatic | 0 | 0 | 0 | 0 | na | 0 | 0 | 0 |  | |  |  |
|  | Aus | 0 | 0 | 0 | 0 | na | 0 | 0 | 0 |  | |  |  |
|  | RufiChina | 0 | 0 | 0 | 0 | na | 0 | 0 | 0 |  | |  |  |
|  | RufiIndia | 0 | 0 | 0 | 0 | na | 1 | 1 | 0 |  | |  |  |
|  | RufiSEAsia | 5 | 0.00607 | 0.00609 | 0.00704 | -0.34835 | 1 | 1 | 0 |  | |  |  |
|  | Nivara | 0 | 0 | 0 | 0 | na | 0 | 0 | 0 |  | |  |  |
|  | Barthii | 0 | 0 | 0 | 0 | na | 0 | 0 | 0 |  | |  |  |
|  | Meridionalis | 0 | 0 | 0 | 0 | na | 1 | 0 | 1 |  | |  |  |
| **sts089** |  | # seg sites | pi silent | pi (JC) | theta silent | Taj D | polymor. loci | synonymous | replacement | |  | |  |
|  | SC | 9 | 0.02306 | 0.02343 | 0.01809 | 1.65186 | 1 | 0 | 1 |  | |  |  |
|  | ARred | 11 | 0.01995 | 0.02022 | 0.00899 | 3.05922 | 1 | 0 | 1 |  | |  |  |
|  | CAred | 0 | 0 | 0 | 0 | na | 0 | 0 | 0 |  | |  |  |
|  |  |  |  |  |  |  | x | x | x |  | |  |  |
|  | Tempjap | 9 | 0.00513 | 0.00515 | 0.0108 | -1.6186 | 0 | 0 | 0 |  | |  |  |
|  |  | 9 | 0.00502 | 0.00503 | 0.01121 | -1.7114 | x | x | x |  | |  |  |
|  |  | 0 | 0 | 0 | 0 | na | x | x | x |  | |  |  |
|  | CAcult | 0 | 0 | 0 | 0 | na | 0 | 0 | 0 |  | |  |  |
|  | ARcult | 9 | 0.02155 | 0.02187 | 0.01244 | 2.47866 | 0 | 0 | 0 |  | |  |  |
|  |  | 10 | 0.02535 | 0.02579 | 0.01539 | 2.65478 | x | x | x |  | |  |  |
|  | indica | 10 | 0.01774 | 0.01795 | 0.01086 | 1.41766 | 2 | 1 | 1 |  | |  |  |
|  | SHWeedy | 10 | 0.01397 | 0.01411 | 0.01073 | 1.01571 | 1 | 0 | 1 |  | |  |  |
|  | MixWeedy | 10 | 0.02478 | 0.0252 | 0.01643 | 2.10873 | 1 | 0 | 1 |  | |  |  |
|  | BrHWeedy | 10 | 0.01652 | 0.01671 | 0.01643 | 0.02645 | 1 | 0 | 1 |  | |  |  |
|  | BHWeedy | 10 | 0.01318 | 0.0133 | 0.01047 | 0.77154 | 1 | 0 | 1 |  | |  |  |
|  | Rufi | 18 | 0.02483 | 0.02525 | 0.01784 | 1.00563 |  |  |  |  | |  |  |
|  |  | 10 | 0.03442 | 0.03524 | 0.02816 | 2.24122 | x | x | x |  | |  |  |
|  | Glumae | 2 | 0.00688 | 0.00692 | 0.00563 | 2.01187 | 1 | 1 | 0 |  | |  |  |
|  |  | 0 | 0 | 0 | 0 | na | x | x | x |  | |  |  |
|  | glaber | 0 | 0 | 0 | 0 | na | 0 | 0 | 0 |  | |  |  |
|  |  |  |  |  |  |  | x | x | x |  | |  |  |
|  | PA_rice | 0 | 0 | 0 | 0 |  | 0 | 0 | 0 |  | |  |  |
|  |  |  |  |  |  |  |  |  |  |  | |  |  |
|  | Updated Seq Set Analyses | | |  |  |  |  |  |  |  | |  |  |
|  |  |  |  |  |  |  |  |  |  |  | |  |  |
|  |  | # seg sites | pi silent | pi (JC) | theta silent | Taj D |  |  |  |  | |  |  |
|  | CAredWell | 0 | 0 | 0 | 0 | na | 0 | 0 | 0 |  | |  |  |
|  | DemmerWest1 | 0 | 0 | 0 | 0 | na | 0 | 0 | 0 |  | |  |  |
|  | DemmerWest2 | 0 | 0 | 0 | 0 | na | 0 | 0 | 0 |  | |  |  |
|  | DemmerEast | 0 | 0 | 0 | 0 | na | 0 | 0 | 0 |  | |  |  |
|  | TropJap | 9 | 0.00305 | 0.00305 | 0.00997 | -1.95951 | 0 | 0 | 0 |  | |  |  |
|  | Aromatic | 0 | 0 | 0 | 0 | na | 0 | 0 | 0 |  | |  |  |
|  | Aus | 10 | 0.02451 | 0.02492 | 0.01461 | 2.60512 | 1 | 0 | 1 |  | |  |  |
|  | RufiChina | 9 | 0.01931 | 0.01957 | 0.01275 | 1.82393 | 1 | 1 | 0 |  | |  |  |
|  | RufiIndia | 12 | 0.02341 | 0.02378 | 0.01547 | 1.6533 | 1 | 1 | 0 |  | |  |  |
|  | RufiSEAsia | 14 | 0.02455 | 0.02496 | 0.01689 | 1.1253 | 1 | 1 | 0 |  | |  |  |
|  | Nivara | 10 | 0.02754 | 0.02806 | 0.02261 | 1.32483 | 0 | 0 | 0 |  | |  |  |
|  | Barthii | 0 | 0 | 0 | 0 | na | 0 | 0 | 0 |  | |  |  |
|  | Meridionalis | 0 | 0 | 0 | 0 | na | 0 | 0 | 0 |  | |  |  |
| **sts090** |  | # seg sites | pi silent | pi (JC) | theta silent | Taj D | polymor. loci | synonymous | replacement | |  | |  |
|  | SC | 1 | 0 | 0 | 0 | 1.4451 | 0 | 0 | 0 |  | |  |  |
|  | ARred | 5 | 0.00085 | 0.00085 | 0.00234 | -0.72935 | 0 | 0 | 0 |  | |  |  |
|  | CAred | 0 | 0 | 0 | 0 | na | 0 | 0 | 0 |  | |  |  |
|  |  |  |  |  |  |  | x | x | x |  | |  |  |
|  | Tempjap | 7 | 0.00455 | 0.00456 | 0.00548 | -0.50411 | 0 | 0 | 0 |  | |  |  |
|  |  | 0 | 0 | 0 | 0 | NA | x | x | x |  | |  |  |
|  |  | 3 | 0.00359 | 0.0036 | 0.00357 | 0.02107 | x | x | x |  | |  |  |
|  | CAcult | 0 | 0 | 0 | 0 | NA | 0 | 0 | 0 |  | |  |  |
|  | ARcult | 4 | 0.00358 | 0.00359 | 0.00373 | -0.10766 | 0 | 0 | 0 |  | |  |  |
|  |  | 0 | 0 | 0 | 0 | NA | x | x | x |  | |  |  |
|  | indica | 4 | 0.00511 | 0.00513 | 0.00313 | 1.49386 | 0 | 0 | 0 |  | |  |  |
|  | SHWeedy | 2 | 0.00029 | 0.00029 | 0.00077 | -0.55293 | 0 | 0 | 0 |  | |  |  |
|  | MixWeedy | 3 | 0.00538 | 0.0054 | 0.00357 | 1.83053 | 0 | 0 | 0 |  | |  |  |
|  | BrHWeedy | 0 | 0 | 0 | 0 | NA | 0 | 0 | 0 |  | |  |  |
|  | BHWeedy | 2 | 0.00153 | 0.00153 | 0.00076 | 1.92721 | 0 | 0 | 0 |  | |  |  |
|  | Rufi | 7 | 0.00458 | 0.00459 | 0.00985 | -1.24334 |  |  |  |  | |  |  |
|  |  | 1 | 0.00224 | 0.00225 | 0.00184 | 1.63299 | x | x | x |  | |  |  |
|  | Glumae | 0 | 0 | 0 | 0 | NA | 0 | 0 | 0 |  | |  |  |
|  |  | 3 | 0.00673 | 0.00676 | 0.00551 | 2.01187 | x | x | x |  | |  |  |
|  | glaber | 0 | 0 | 0 | 0 | NA | 0 | 0 | 0 |  | |  |  |
|  |  |  |  |  |  |  | x | x | x |  | |  |  |
|  | PA_rice | 5 | 0.00897 | 0.00903 | 0.00734 |  | 0 | 0 | 0 |  | |  |  |
|  |  |  |  |  |  |  |  |  |  |  | |  |  |
|  | Updated Seq Set Analyses | | |  |  |  |  |  |  |  | |  |  |
|  |  |  |  |  |  |  |  |  |  |  | |  |  |
|  |  | # seg sites | pi silent | pi (JC) | theta silent | Taj D |  |  |  |  | |  |  |
|  | CAredWell | 0 | 0 | 0 | 0 | na | 0 | 0 | 0 |  | |  |  |
|  | DemmerWest1 | 0 | 0 | 0 | 0 | na | 0 | 0 | 0 |  | |  |  |
|  | DemmerWest2 | 0 | 0 | 0 | 0 | na | 0 | 0 | 0 |  | |  |  |
|  | DemmerEast | 0 | 0 | 0 | 0 | na | 0 | 0 | 0 |  | |  |  |
|  | TropJap | 0 | 0 | 0 | 0 | na | 0 | 0 | 0 |  | |  |  |
|  | Aromatic | 3 | 0.00359 | 0.0036 | 0.00357 | 0.02107 | 0 | 0 | 0 |  | |  |  |
|  | Aus | 0 | 0 | 0 | 0 | na | 0 | 0 | 0 |  | |  |  |
|  | RufiChina | 3 | 0.00364 | 0.00365 | 0.00271 | 1.35917 | 0 | 0 | 0 |  | |  |  |
|  | RufiIndia | 9 | 0.00944 | 0.0095 | 0.00953 | 0.05677 | 0 | 0 | 0 |  | |  |  |
|  | RufiSEAsia | 11 | 0.00413 | 0.00414 | 0.00796 | -1.53048 | 1 | 1 | 0 |  | |  |  |
|  | Nivara | 1 | 0.00179 | 0.0018 | 0.00147 | 0.85057 | 0 | 0 | 0 |  | |  |  |
|  | Barthii | 7 | 0.01273 | 0.01284 | 0.01046 | 1.26744 | 1 | 1 | 0 |  | |  |  |
|  | Meridionalis | 0 | 0 | 0 | 0 | na | 0 | 0 | 0 |  | |  |  |
| **sts099** |  | # seg sites | pi silent | pi (JC) | theta silent | Taj D | polymor. loci | synonymous | replacement | |  | |  |
|  | SC | 0 | 0 | 0 | 0 | NA | 0 | 0 | 0 |  | |  |  |
|  | ARred | 3 | 0.00363 | 0.00364 | 0.00235 | 0.88621 | 1 | 1 | 0 |  | |  |  |
|  | CAred | 0 | 0 | 0 | 0 | na | 0 | 0 | 0 |  | |  |  |
|  |  |  |  |  |  |  | x | x | x |  | |  |  |
|  | Tempjap | 0 | 0 | 0 | 0 | NA | 0 | 0 | 0 |  | |  |  |
|  |  | 0 | 0 | 0 | 0 | NA | x | x | x |  | |  |  |
|  |  | 0 | 0 | 0 | 0 | NA | x | x | x |  | |  |  |
|  | CAcult | 0 | 0 | 0 | 0 | NA | 0 | 0 | 0 |  | |  |  |
|  | ARcult | 0 | 0 | 0 | 0 | NA | 0 | 0 | 0 |  | |  |  |
|  |  | 0 | 0 | 0 | 0 | NA | x | x | x |  | |  |  |
|  | indica | 3 | 0.00327 | 0.00327 | 0.00314 | 0.08507 | 2 | 2 | 0 |  | |  |  |
|  | SHWeedy | 2 | 0.00453 | 0.00455 | 0.00203 | 2.21789 | 1 | 1 | 0 |  | |  |  |
|  | MixWeedy | 3 | 0.00631 | 0.00634 | 0.00478 | 1.15198 | 1 | 1 | 0 |  | |  |  |
|  | BrHWeedy | 0 | 0 | 0 | 0 | NA | 0 | 0 | 0 |  | |  |  |
|  | BHWeedy | 0 | 0 | 0 | 0 | NA | 0 | 0 | 0 |  | |  |  |
|  | Rufi | 26 | 0.02349 | 0.02387 | 0.02356 | -0.00894 |  |  |  |  | |  |  |
|  |  | 2 | 0.00601 | 0.00603 | 0.00492 | 1.89306 | x | x | x |  | |  |  |
|  | Glumae | 0 | 0 | 0 | 0 | NA | 0 | 0 | 0 |  | |  |  |
|  |  | 0 | 0 | 0 | 0 | NA | x | x | x |  | |  |  |
|  | glaber | 0 | 0 | 0 | 0 | NA | 0 | 0 | 0 |  | |  |  |
|  |  |  |  |  |  |  | x | x | x |  | |  |  |
|  | PA_rice | 3 | 0.00676 | 0.00679 | 0.00738 |  | 1 | 1 | 0 |  | |  |  |
|  |  |  |  |  |  |  |  |  |  |  | |  |  |
|  | Updated Seq Set Analyses | | |  |  |  |  |  |  |  | |  |  |
|  |  |  |  |  |  |  |  |  |  |  | |  |  |
|  |  | # seg sites | pi silent | pi (JC) | theta silent | Taj D |  |  |  |  | |  |  |
|  | CAredWell | 0 | 0 | 0 | 0 | na | 0 | 0 | 0 |  | |  |  |
|  | DemmerWest1 | 0 | 0 | 0 | 0 | na | 0 | 0 | 0 |  | |  |  |
|  | DemmerWest2 | 0 | 0 | 0 | 0 | na | 0 | 0 | 0 |  | |  |  |
|  | DemmerEast | 0 | 0 | 0 | 0 | na | 0 | 0 | 0 |  | |  |  |
|  | TropJap | 0 | 0 | 0 | 0 | na | 0 | 0 | 0 |  | |  |  |
|  | Aromatic | 0 | 0 | 0 | 0 | na | 0 | 0 | 0 |  | |  |  |
|  | Aus | 0 | 0 | 0 | 0 | na | 0 | 0 | 0 |  | |  |  |
|  | RufiChina | 3 | 0.00193 | 0.00193 | 0.00371 | -1.23957 | 1 | 1 | 0 |  | |  |  |
|  | RufiIndia | 14 | 0.02723 | 0.02774 | 0.01574 | 2.40438 | 5 | 5 | 0 |  | |  |  |
|  | RufiSEAsia | 17 | 0.02643 | 0.02691 | 0.01894 | 1.2798 | 6 | 6 | 0 |  | |  |  |
|  | Nivara | 2 | 0.00481 | 0.00482 | 0.00395 | 1.03194 | 1 | 1 | 0 |  | |  |  |
|  | Barthii | 0 | 0 | 0 | 0 | na | 0 | 0 | 0 |  | |  |  |
|  | Meridionalis | 0 | 0 | 0 | 0 | na | 0 | 0 | 0 |  | |  |  |
| **sts102** |  | # seg sites | pi silent | pi (JC) | theta silent | Taj D | polymor. loci | synonymous | replacement | |  | |  |
|  | SC | 0 | 0 | 0 | 0 | NA | 0 | 0 | 0 |  | |  |  |
|  | ARred | 0 | 0 | 0 | 0 | NA | 0 | 0 | 0 |  | |  |  |
|  | CAred | 0 | 0 | 0 | 0 | NA | 0 | 0 | 0 |  | |  |  |
|  |  |  |  |  |  |  | x | x | x |  | |  |  |
|  | Tempjap | 0 | 0 | 0 | 0 | NA | 0 | 0 | 0 |  | |  |  |
|  |  | 1 | 0 | 0 | 0 | -0.81338 | x | x | x |  | |  |  |
|  |  | 0 | 0 | 0 | 0 | NA | x | x | x |  | |  |  |
|  | CAcult | 0 | 0 | 0 | 0 | NA | 0 | 0 | 0 |  | |  |  |
|  | ARcult | 1 | 0 | 0 | 0 | -0.6811 | 1 | 0 | 1 |  | |  |  |
|  |  | 1 | 0.00338 | 0.00339 | 0.00205 | 1.48617 | x | x | x |  | |  |  |
|  | indica | 2 | 0.00316 | 0.00317 | 0.00144 | 0.55807 | 2 | 1 | 1 |  | |  |  |
|  | SHWeedy | 0 | 0 | 0 | 0 | NA | 0 | 0 | 0 |  | |  |  |
|  | MixWeedy | 0 | 0 | 0 | 0 | NA | 0 | 0 | 0 |  | |  |  |
|  | BrHWeedy | 0 | 0 | 0 | 0 | NA | 0 | 0 | 0 |  | |  |  |
|  | BHWeedy | 0 | 0 | 0 | 0 | NA | 0 | 0 | 0 |  | |  |  |
|  | Rufi | 7 | 0.00183 | 0.00183 | 0.0071 | -1.3414 |  |  |  |  | |  |  |
|  |  | 0 | 0 | 0 | 0 | 1.63299 | x | x | x |  | |  |  |
|  | Glumae | 0 | 0 | 0 | 0 | 1.89306 | 2 | 1 | 1 |  | |  |  |
|  |  | 0 | 0 | 0 | 0 | NA | x | x | x |  | |  |  |
|  | glaber | 0 | 0 | 0 | 0 | NA | 0 | 0 | 0 |  | |  |  |
|  |  |  |  |  |  |  | x | x | x |  | |  |  |
|  | PA_rice | 0 | 0 | 0 | 0 |  | 0 | 0 | 0 |  | |  |  |
|  |  |  |  |  |  |  |  |  |  |  | |  |  |
|  | Updated Seq Set Analyses | | |  |  |  |  |  |  |  | |  |  |
|  |  |  |  |  |  |  |  |  |  |  | |  |  |
|  |  | # seg sites | pi silent | pi (JC) | theta silent | Taj D |  |  |  |  | |  |  |
|  | CAredWell | 0 | 0 | 0 | 0 | na | 0 | 0 | 0 |  | |  |  |
|  | DemmerWest1 | 0 | 0 | 0 | 0 | na | 0 | 0 | 0 |  | |  |  |
|  | DemmerWest2 | 0 | 0 | 0 | 0 | na | 0 | 0 | 0 |  | |  |  |
|  | DemmerEast | 0 | 0 | 0 | 0 | na | 0 | 0 | 0 |  | |  |  |
|  | TropJap | 1 | 0 | 0 | 0 | -0.8882 | 1 | 0 | 1 |  | |  |  |
|  | Aromatic | 0 | 0 | 0 | 0 | na | 0 | 0 | 0 |  | |  |  |
|  | Aus | 1 | 0.00327 | 0.00327 | 0.00195 | 1.43413 | 1 | 1 | 0 |  | |  |  |
|  | RufiChina | 3 | 0.00164 | 0.00164 | 0.0034 | -1.45662 | 4 | 3 | 1 |  | |  |  |
|  | RufiIndia | 2 | 0.00039 | 0.00039 | 0.00154 | 0.25842 | 2 | 1 | 1 |  | |  |  |
|  | RufiSEAsia | 4 | 0.00226 | 0.00226 | 0.00432 | -0.64518 | 4 | 3 | 1 |  | |  |  |
|  | Nivara | 0 | 0 | 0 | 0 | na | 1 | 1 | 0 |  | |  |  |
|  | Barthii | 0 | 0 | 0 | 0 | na | 0 | 0 | 0 |  | |  |  |
|  | Meridionalis | 0 | 0 | 0 | 0 | na | 0 | 0 | 0 |  | |  |  |
| **sts104** |  | # seg sites | pi silent | pi (JC) | theta silent | Taj D | polymor. loci | synonymous | replacement | |  | |  |
|  | SC | 0 | 0 | 0 | 0 | NA | 0 | 0 | 0 |  | |  |  |
|  | ARred | 1 | 0.0021 | 0.0021 | 0.0066 | -0.70363 | 0 | 0 | 0 |  | |  |  |
|  | CAred | 0 | 0 | 0 | 0 | na | 0 | 0 | 0 |  | |  |  |
|  |  |  |  |  |  |  | x | x | x |  | |  |  |
|  | Tempjap | 0 | 0 | 0 | 0 | NA | 0 | 0 | 0 |  | |  |  |
|  |  | 1 | 0.00041 | 0.00041 | 0.00091 | -1.07337 | x | x | x |  | |  |  |
|  |  | 1 | 0.00183 | 0.00184 | 0.00125 | 1.06589 | x | x | x |  | |  |  |
|  | CAcult | 0 | 0 | 0 | 0 | NA | 0 | 0 | 0 |  | |  |  |
|  | ARcult | 1 | 0.00064 | 0.00064 | 0.00108 | -0.68111 | 0 | 0 | 0 |  | |  |  |
|  |  | 1 | 0.00115 | 0.00115 | 0.00125 | -0.19492 | x | x | x |  | |  |  |
|  | indica | 0 | 0 | 0 | 0 | -0.33848 | 1 | 0 | 1 |  | |  |  |
|  | SHWeedy | 0 | 0 | 0 | 0 | NA | 0 | 0 | 0 |  | |  |  |
|  | MixWeedy | 1 | 0.00202 | 0.00202 | 0.00134 | 1.30268 | 0 | 0 | 0 |  | |  |  |
|  | BrHWeedy | 0 | 0 | 0 | 0 | NA | 0 | 0 | 0 |  | |  |  |
|  | BHWeedy | 1 | 0.00033 | 0.00033 | 0.0091 | -0.86644 | 0 | 0 | 0 |  | |  |  |
|  | Rufi | 28 | 0.00923 | 0.00929 | 0.01906 | -1.61434 |  |  |  |  | |  |  |
|  |  | 3 | 0.00756 | 0.0076 | 0.00619 | 2.01187 | x | x | x |  | |  |  |
|  | Glumae | 0 | 0 | 0 | 0 | NA | 0 | 0 | 0 |  | |  |  |
|  |  | 0 | 0 | 0 | 0 | NA | x | x | x |  | |  |  |
|  | glaber | 0 | 0 | 0 | 0 | NA | 0 | 0 | 0 |  | |  |  |
|  |  |  |  |  |  |  | x | x | x |  | |  |  |
|  | PA_rice | 1 | 0.00189 | 0.00189 | 0.00206 |  | 0 | 0 | 0 |  | |  |  |
|  |  |  |  |  |  |  |  |  |  |  | |  |  |
|  | Updated Seq Set Analyses | | |  |  |  |  |  |  |  | |  |  |
|  |  |  |  |  |  |  |  |  |  |  | |  |  |
|  |  | # seg sites | pi silent | pi (JC) | theta silent | Taj D |  |  |  |  | |  |  |
|  | CAredWell | 0 | 0 | 0 | 0 | na | 0 | 0 | 0 |  | |  |  |
|  | DemmerWest1 | 0 | 0 | 0 | 0 | na | 0 | 0 | 0 |  | |  |  |
|  | DemmerWest2 | 0 | 0 | 0 | 0 | na | 0 | 0 | 0 |  | |  |  |
|  | DemmerEast | 0 | 0 | 0 | 0 | na | 0 | 0 | 0 |  | |  |  |
|  | TropJap | 1 | 0.00049 | 0.00049 | 0.00082 | -0.93148 | 1 | 0 | 1 |  | |  |  |
|  | Aromatic | 1 | 0.00183 | 0.00184 | 0.00125 | 1.06589 | 0 | 0 | 0 |  | |  |  |
|  | Aus | 1 | 0.00166 | 0.00166 | 0.00119 | 0.84228 | 0 | 0 | 0 |  | |  |  |
|  | RufiChina | 4 | 0.00252 | 0.00252 | 0.00311 | -0.65517 | 3 | 1 | 2 |  | |  |  |
|  | RufiIndia | 6 | 0.00718 | 0.00721 | 0.00563 | 0.77179 | 1 | 1 | 0 |  | |  |  |
|  | RufiSEAsia | 8 | 0.00778 | 0.00782 | 0.00615 | 0.20913 | 4 | 2 | 2 |  | |  |  |
|  | Nivara | 3 | 0.00605 | 0.00607 | 0.00497 | 1.12414 | 1 | 1 | 0 |  | |  |  |
|  | Barthii | 0 | 0 | 0 | 0 | na | 0 | 0 | 0 |  | |  |  |
|  | Meridionalis | 0 | 0 | 0 | 0 | na | 0 | 0 | 0 |  | |  |  |
| **sts108** |  | # seg sites | pi silent | pi (JC) | theta silent | Taj D | polymor. loci | synonymous | replacement | |  | |  |
|  | SC | 0 | 0 | 0 | 0 | NA | 0 | 0 | 0 |  | |  |  |
|  | ARred | 0 | 0 | 0 | 0 | NA | 0 | 0 | 0 |  | |  |  |
|  | CAred | 0 | 0 | 0 | 0 | na | 0 | 0 | 0 |  | |  |  |
|  |  |  |  |  |  |  | x | x | x |  | |  |  |
|  | Tempjap | 0 | 0 | 0 | 0 | NA | 0 | 0 | 0 |  | |  |  |
|  |  | 0 | 0 | 0 | 0 | NA | x | x | x |  | |  |  |
|  |  | 0 | 0 | 0 | 0 | NA | x | x | x |  | |  |  |
|  | CAcult | 0 | 0 | 0 | 0 | NA | 0 | 0 | 0 |  | |  |  |
|  | ARcult | 0 | 0 | 0 | 0 | NA | 0 | 0 | 0 |  | |  |  |
|  |  | 0 | 0 | 0 | 0 | NA | x | x | x |  | |  |  |
|  | indica | 0 | 0 | 0 | 0 | NA | 0 | 0 | 0 |  | |  |  |
|  | SHWeedy | 0 | 0 | 0 | 0 | NA | 0 | 0 | 0 |  | |  |  |
|  | MixWeedy | 0 | 0 | 0 | 0 | NA | 0 | 0 | 0 |  | |  |  |
|  | BrHWeedy | 0 | 0 | 0 | 0 | NA | 0 | 0 | 0 |  | |  |  |
|  | BHWeedy | 0 | 0 | 0 | 0 | NA | 0 | 0 | 0 |  | |  |  |
|  | Rufi | 12 | 0.00371 | 0.00372 | 0.01364 | -1.96296 |  |  |  |  | |  |  |
|  |  | 0 | 0 | 0 | 0 | NA | x | x | x |  | |  |  |
|  | Glumae | 0 | 0 | 0 | 0 | NA | 0 | 0 | 0 |  | |  |  |
|  |  | 0 | 0 | 0 | 0 | NA | x | x | x |  | |  |  |
|  | glaber | 0 | 0 | 0 | 0 | NA | 0 | 0 | 0 |  | |  |  |
|  |  |  |  |  |  |  | x | x | x |  | |  |  |
|  | PA_rice | 0 | 0 | 0 | 0 |  | 0 | 0 | 0 |  | |  |  |
|  |  |  |  |  |  |  |  |  |  |  | |  |  |
|  | Updated Seq Set Analyses | | |  |  |  |  |  |  |  | |  |  |
|  |  |  |  |  |  |  |  |  |  |  | |  |  |
|  |  | # seg sites | pi silent | pi (JC) | theta silent | Taj D |  |  |  |  | |  |  |
|  | CAredWell | 0 | 0 | 0 | 0 | na | 0 | 0 | 0 |  | |  |  |
|  | DemmerWest1 | 0 | 0 | 0 | 0 | na | 0 | 0 | 0 |  | |  |  |
|  | DemmerWest2 | 0 | 0 | 0 | 0 | na | 0 | 0 | 0 |  | |  |  |
|  | DemmerEast | 0 | 0 | 0 | 0 | na | 0 | 0 | 0 |  | |  |  |
|  | TropJap | 0 | 0 | 0 | 0 | na | 0 | 0 | 0 |  | |  |  |
|  | Aromatic | 0 | 0 | 0 | 0 | na | 0 | 0 | 0 |  | |  |  |
|  | Aus | 0 | 0 | 0 | 0 | na | 0 | 0 | 0 |  | |  |  |
|  | RufiChina | 1 | 0.00103 | 0.00103 | 0.00163 | -0.64112 | 0 | 0 | 0 |  | |  |  |
|  | RufiIndia | 2 | 0.00144 | 0.00144 | 0.00296 | -1.03052 | 0 | 0 | 0 |  | |  |  |
|  | RufiSEAsia | 10 | 0.00576 | 0.00578 | 0.01383 | -1.72437 | 5 | 5 | 0 |  | |  |  |
|  | Nivara | 1 | 0.00317 | 0.00318 | 0.00261 | 0.85057 | 0 | 0 | 0 |  | |  |  |
|  | Barthii | 0 | 0 | 0 | 0 | na | 0 | 0 | 0 |  | |  |  |
|  | Meridionalis | 1 | 0.00397 | 0.00398 | 0.00325 | 1.63299 | 1 | 1 | 0 |  | |  |  |
| **sts113** |  | # seg sites | pi silent | pi (JC) | theta silent | Taj D | polymor. loci | synonymous | replacement | |  | |  |
|  | SC | 0 | 0 | 0 | 0 | NA | 0 | 0 | 0 |  | |  |  |
|  | ARred | 1 | 0.00017 | 0.00017 | 0.00065 | -0.76387 | 1 | 1 | 0 |  | |  |  |
|  | CAred | 0 | 0 | 0 | 0 | na | 0 | 0 | 0 |  | |  |  |
|  |  |  |  |  |  |  | x | x | x |  | |  |  |
|  | Tempjap | 0 | 0 | 0 | 0 | NA | 0 | 0 | 0 |  | |  |  |
|  |  | 1 | 0.0004 | 0.0004 | 0.0009 | -0.81338 | x | x | x |  | |  |  |
|  |  | 1 | 0.00113 | 0.00113 | 0.00123 | -0.19492 | x | x | x |  | |  |  |
|  | CAcult | 0 | 0 | 0 | 0 | NA | 0 | 0 | 0 |  | |  |  |
|  | ARcult | 1 | 0.0006 | 0.0006 | 0.00101 | -0.68111 | 1 | 1 | 0 |  | |  |  |
|  |  | 0 | 0 | 0 | 0 | NA | x | x | x |  | |  |  |
|  | indica | 0 | 0 | 0 | 0 | NA | 0 | 0 | 0 |  | |  |  |
|  | SHWeedy | 0 | 0 | 0 | 0 | NA | 0 | 0 | 0 |  | |  |  |
|  | MixWeedy | 1 | 0.00174 | 0.00174 | 0.00132 | 0.8198 | 1 | 1 | 0 |  | |  |  |
|  | BrHWeedy | 0 | 0 | 0 | 0 | NA | 0 | 0 | 0 |  | |  |  |
|  | BHWeedy | 0 | 0 | 0 | 0 | NA | 0 | 0 | 0 |  | |  |  |
|  | Rufi | 13 | 0.0016 | 0.0016 | 0.00782 | -2.1774 |  |  |  |  | |  |  |
|  |  | 0 | 0 | 0 | 0 | NA | x | x | x |  | |  |  |
|  | Glumae | 0 | 0 | 0 | 0 | NA | 0 | 0 | 0 |  | |  |  |
|  |  | 0 | 0 | 0 | 0 | NA | x | x | x |  | |  |  |
|  | glaber | 0 | 0 | 0 | 0 | NA | 0 | 0 | 0 |  | |  |  |
|  |  |  |  |  |  |  | x | x | x |  | |  |  |
|  | PA_rice | 0 | 0 | 0 | 0 |  | 0 | 0 | 0 |  | |  |  |
|  |  |  |  |  |  |  |  |  |  |  | |  |  |
|  | Updated Seq Set Analyses | | |  |  |  |  |  |  |  | |  |  |
|  |  |  |  |  |  |  |  |  |  |  | |  |  |
|  |  | # seg sites | pi silent | pi (JC) | theta silent | Taj D |  |  |  |  | |  |  |
|  | CAredWell | 0 | 0 | 0 | 0 | na | 0 | 0 | 0 |  | |  |  |
|  | DemmerWest1 | 0 | 0 | 0 | 0 | na | 0 | 0 | 0 |  | |  |  |
|  | DemmerWest2 | 0 | 0 | 0 | 0 | na | 0 | 0 | 0 |  | |  |  |
|  | DemmerEast | 0 | 0 | 0 | 0 | na | 0 | 0 | 0 |  | |  |  |
|  | TropJap | 1 | 0.00024 | 0.00024 | 0.0008 | -0.8912 | 1 | 1 | 0 |  | |  |  |
|  | Aromatic | 1 | 0.00113 | 0.00113 | 0.00123 | -0.19492 | 1 | 1 | 0 |  | |  |  |
|  | Aus | 0 | 0 | 0 | 0 | na | 0 | 0 | 0 |  | |  |  |
|  | RufiChina | 2 | 0.00034 | 0.00034 | 0.00102 | -0.52596 | 4 | 1 | 3 |  | |  |  |
|  | RufiIndia | 2 | 0.00047 | 0.00047 | 0.00185 | -1.59159 | 4 | 3 | 1 |  | |  |  |
|  | RufiSEAsia | 6 | 0.00208 | 0.00208 | 0.00519 | -1.737 | 5 | 2 | 3 |  | |  |  |
|  | Nivara | 0 | 0 | 0 | 0 | na | 1 | 0 | 1 |  | |  |  |
|  | Barthii | 0 | 0 | 0 | 0 | na | 0 | 0 | 0 |  | |  |  |
|  | Meridionalis | 0 | 0 | 0 | 0 | na | 0 | 0 | 0 |  | |  |  |
| **sts116** |  | # seg sites | pi silent | pi (JC) | theta silent | Taj D | polymor. loci | synonymous | replacement | |  | |  |
|  | SC | 0 | 0 | 0 | 0 | na | 0 | 0 | 0 |  | |  |  |
|  | ARred | 1 | 0 | 0 | 0 | -0.91063 | 1 | 0 | 1 |  | |  |  |
|  | CAred | 0 | 0 | 0 | 0 | na | 0 | 0 | 0 |  | |  |  |
|  |  |  |  |  |  |  | x | x | x |  | |  |  |
|  | Tempjap | 0 | 0 | 0 | 0 | na | 0 | 0 | 0 |  | |  |  |
|  |  | 1 | 0 | 0 | 0 | 0.2721 | x | x | x |  | |  |  |
|  |  | 0 | 0 | 0 | 0 | 0.8198 | x | x | x |  | |  |  |
|  | CAcult | 0 | 0 | 0 | 0 | na | 0 | 0 | 0 |  | |  |  |
|  | ARcult | 1 | 0 | 0 | 0 | 0.89527 | 1 | 0 | 1 |  | |  |  |
|  |  | 0 | 0 | 0 | 0 | 1.06589 | x | x | x |  | |  |  |
|  | indica | 1 | 0 | 0 | 0 | 0.35267 | 2 | 1 | 1 |  | |  |  |
|  | SHWeedy | 0 | 0 | 0 | 0 | na | 0 | 0 | 0 |  | |  |  |
|  | MixWeedy | 1 | 0 | 0 | 0 | 1.64145 | 2 | 1 | 1 |  | |  |  |
|  | BrHWeedy | 0 | 0 | 0 | 0 | na | 0 | 0 | 0 |  | |  |  |
|  | BHWeedy | 1 | 0 | 0 | 0 | -1.10686 | 1 | 0 | 1 |  | |  |  |
|  | Rufi | 10 | 0.00133 | 0.00133 | 0.00359 | -1.5055 |  |  |  |  | |  |  |
|  |  | 0 | 0 | 0 | 0 | na | x | x | x |  | |  |  |
|  | Glumae | 1 | 0.004 | 0.00401 | 0.00328 | 1.89306 | 1 | 0 | 1 |  | |  |  |
|  |  | 1 | 0.004 | 0.00401 | 0.00328 | 1.63299 | x | x | x |  | |  |  |
|  | glaber | 0 | 0 | 0 | 0 | na | 0 | 0 | 0 |  | |  |  |
|  |  |  |  |  |  |  | x | x | x |  | |  |  |
|  | PA_rice | 0 | 0 | 0 | 0 |  | 0 | 0 | 0 |  | |  |  |
|  |  |  |  |  |  |  |  |  |  |  | |  |  |
|  | Updated Seq Set Analyses | | |  |  |  |  |  |  |  | |  |  |
|  |  |  |  |  |  |  |  |  |  |  | |  |  |
|  |  | # seg sites | pi silent | pi (JC) | theta silent | Taj D |  |  |  |  | |  |  |
|  | CAredWell | 0 | 0 | 0 | 0 | na | 0 | 0 | 0 |  | |  |  |
|  | DemmerWest1 | 0 | 0 | 0 | 0 | na | 0 | 0 | 0 |  | |  |  |
|  | DemmerWest2 | 0 | 0 | 0 | 0 | na | 0 | 0 | 0 |  | |  |  |
|  | DemmerEast | 0 | 0 | 0 | 0 | na | 0 | 0 | 0 |  | |  |  |
|  | TropJap | 1 | 0 | 0 | 0 | 0.89413 | 1 | 0 | 1 |  | |  |  |
|  | Aromatic | 0 | 0 | 0 | 0 | na | 1 | 1 | 0 |  | |  |  |
|  | Aus | 0 | 0 | 0 | 0 | na | 1 | 1 | 0 |  | |  |  |
|  | RufiChina | 2 | 0.00104 | 0.00104 | 0.00165 | -0.19191 | 2 | 1 | 1 |  | |  |  |
|  | RufiIndia | 4 | 0.00189 | 0.00189 | 0.00303 | -0.75042 | 4 | 2 | 2 |  | |  |  |
|  | RufiSEAsia | 6 | 0.00046 | 0.00046 | 0.00158 | -1.51558 | 7 | 2 | 5 |  | |  |  |
|  | Nivara | 1 | 0 | 0 | 0 | 1.63299 | 1 | 0 | 1 |  | |  |  |
|  | Barthii | 2 | 0.0032 | 0.00321 | 0.00263 | 1.03194 | 1 | 0 | 1 |  | |  |  |
|  | Meridionalis | 0 | 0 | 0 | 0 | na | 1 | 0 | 1 |  | |  |  |
| **sts120** |  | # seg sites | pi silent | pi (JC) | theta silent | Taj D | polymor. loci | synonymous | replacement | |  | |  |
|  | SC | 0 | 0 | 0 | 0 | na | 0 | 0 | 0 |  | |  |  |
|  | ARred | 0 | 0 | 0 | 0 | na | 0 | 0 | 0 |  | |  |  |
|  | CAred | 0 | 0 | 0 | 0 | na | 0 | 0 | 0 |  | |  |  |
|  |  |  |  |  |  |  | x | x | x |  | |  |  |
|  | Tempjap | 0 | 0 | 0 | 0 | na | 0 | 0 | 0 |  | |  |  |
|  |  | 0 | 0 | 0 | 0 | na | x | x | x |  | |  |  |
|  |  | 0 | 0 | 0 | 0 | na | x | x | x |  | |  |  |
|  | CAcult | 0 | 0 | 0 | 0 | na | 0 | 0 | 0 |  | |  |  |
|  | ARcult | 0 | 0 | 0 | 0 | na | 0 | 0 | 0 |  | |  |  |
|  |  | 0 | 0 | 0 | 0 | na | x | x | x |  | |  |  |
|  | indica | 0 | 0 | 0 | 0 | na | 0 | 0 | 0 |  | |  |  |
|  | SHWeedy | 0 | 0 | 0 | 0 | na | 0 | 0 | 0 |  | |  |  |
|  | MixWeedy | 0 | 0 | 0 | 0 | na | 0 | 0 | 0 |  | |  |  |
|  | BrHWeedy | 0 | 0 | 0 | 0 | na | 0 | 0 | 0 |  | |  |  |
|  | BHWeedy | 0 | 0 | 0 | 0 | na | 0 | 0 | 0 |  | |  |  |
|  | Rufi | 7 | 0.00165 | 0.00165 | 0.00484 | -1.62863 |  |  |  |  | |  |  |
|  |  | 0 | 0 | 0 | 0 | na | x | x | x |  | |  |  |
|  | Glumae | 0 | 0 | 0 | 0 | na | 0 | 0 | 0 |  | |  |  |
|  |  | 3 | 0.00583 | 0.00585 | 0.00477 | 2.01187 | x | x | x |  | |  |  |
|  | glaber | 0 | 0 | 0 | 0 | na | 0 | 0 | 0 |  | |  |  |
|  |  |  |  |  |  |  | x | x | x |  | |  |  |
|  | PA_rice | 0 | 0 | 0 | 0 |  | 0 | 0 | 0 |  | |  |  |
|  |  |  |  |  |  |  |  |  |  |  | |  |  |
|  | Updated Seq Set Analyses | | |  |  |  |  |  |  |  | |  |  |
|  |  |  |  |  |  |  |  |  |  |  | |  |  |
|  |  | # seg sites | pi silent | pi (JC) | theta silent | Taj D |  |  |  |  | |  |  |
|  | CAredWell | 0 | 0 | 0 | 0 | na | 0 | 0 | 0 |  | |  |  |
|  | DemmerWest1 | 0 | 0 | 0 | 0 | na | 0 | 0 | 0 |  | |  |  |
|  | DemmerWest2 | 0 | 0 | 0 | 0 | na | 0 | 0 | 0 |  | |  |  |
|  | DemmerEast | 0 | 0 | 0 | 0 | na | 0 | 0 | 0 |  | |  |  |
|  | TropJap | 0 | 0 | 0 | 0 | na | 0 | 0 | 0 |  | |  |  |
|  | Aromatic | 0 | 0 | 0 | 0 | na | 0 | 0 | 0 |  | |  |  |
|  | Aus | 0 | 0 | 0 | 0 | na | 0 | 0 | 0 |  | |  |  |
|  | RufiChina | 0 | 0 | 0 | 0 | na | 0 | 0 | 0 |  | |  |  |
|  | RufiIndia | 2 | 0.00204 | 0.00205 | 0.0018 | 0.27474 | 0 | 0 | 0 |  | |  |  |
|  | RufiSEAsia | 2 | 0.00098 | 0.00098 | 0.00168 | -1.03425 | 0 | 0 | 0 |  | |  |  |
|  | Nivara | 0 | 0 | 0 | 0 | na | 0 | 0 | 0 |  | |  |  |
|  | Barthii | 3 | 0.00466 | 0.00468 | 0.00383 | 1.18059 | 1 | 0 | 1 |  | |  |  |
|  | Meridionalis | 0 | 0 | 0 | 0 | na | 0 | 0 | 0 |  | |  |  |
| **sts121** |  | # seg sites | pi silent | pi (JC) | theta silent | Taj D | polymor. loci | synonymous | replacement | |  | |  |
|  | SC | 0 | 0 | 0 | 0 | na | 0 | 0 | 0 |  | |  |  |
|  | ARred | 0 | 0 | 0 | 0 | na | 0 | 0 | 0 |  | |  |  |
|  | CAred | 0 | 0 | 0 | 0 | na | 0 | 0 | 0 |  | |  |  |
|  |  | 1 | 0 | 0 | 0 | -1.05482 | x | x | x |  | |  |  |
|  | Tempjap | 0 | 0 | 0 | 0 | na | 0 | 0 | 0 |  | |  |  |
|  |  | 1 | 0.00109 | 0.00109 | 0.00092 | 0.2721 | x | x | x |  | |  |  |
|  |  | 0 | 0 | 0 | 0 | na | x | x | x |  | |  |  |
|  | CAcult | 0 | 0 | 0 | 0 | na | 0 | 0 | 0 |  | |  |  |
|  | ARcult | 0 | 0 | 0 | 0 | na | 0 | 0 | 0 |  | |  |  |
|  |  | 0 | 0 | 0 | 0 | na | x | x | x |  | |  |  |
|  | indica | 0 | 0 | 0 | 0 | na | 0 | 0 | 0 |  | |  |  |
|  | SHWeedy | 0 | 0 | 0 | 0 | na | 0 | 0 | 0 |  | |  |  |
|  | MixWeedy | 0 | 0 | 0 | 0 | na | 0 | 0 | 0 |  | |  |  |
|  | BrHWeedy | 0 | 0 | 0 | 0 | na | 0 | 0 | 0 |  | |  |  |
|  | BHWeedy | 0 | 0 | 0 | 0 | na | 0 | 0 | 0 |  | |  |  |
|  | Rufi | 15 | 0.00306 | 0.00307 | 0.00949 | -1.9999 |  |  |  |  | |  |  |
|  |  | 0 | 0 | 0 | 0 | na | x | x | x |  | |  |  |
|  | Glumae | 0 | 0 | 0 | 0 | na | 0 | 0 | 0 |  | |  |  |
|  |  | 0 | 0 | 0 | 0 | na | x | x | x |  | |  |  |
|  | glaber | 0 | 0 | 0 | 0 | na | 0 | 0 | 0 |  | |  |  |
|  |  | 0 | 0 | 0 | 0 | na | x | x | x |  | |  |  |
|  | PA_rice | 0 | 0 | 0 | 0 |  | 0 | 0 | 0 |  | |  |  |
|  |  |  |  |  |  |  |  |  |  |  | |  |  |
|  | Updated Seq Set Analyses | | |  |  |  |  |  |  |  | |  |  |
|  |  |  |  |  |  |  |  |  |  |  | |  |  |
|  |  | # seg sites | pi silent | pi (JC) | theta silent | Taj D |  |  |  |  | |  |  |
|  | CAredWell | 0 | 0 | 0 | 0 | na | 0 | 0 | 0 |  | |  |  |
|  | DemmerWest1 | 0 | 0 | 0 | 0 | na | 0 | 0 | 0 |  | |  |  |
|  | DemmerWest2 | 0 | 0 | 0 | 0 | na | 0 | 0 | 0 |  | |  |  |
|  | DemmerEast | 0 | 0 | 0 | 0 | na | 0 | 0 | 0 |  | |  |  |
|  | TropJap | 1 | 0.0007 | 0.0007 | 0.00082 | -0.18789 | 0 | 0 | 0 |  | |  |  |
|  | Aromatic | 0 | 0 | 0 | 0 | na | 0 | 0 | 0 |  | |  |  |
|  | Aus | 0 | 0 | 0 | 0 | na | 0 | 0 | 0 |  | |  |  |
|  | RufiChina | 3 | 0.00384 | 0.00385 | 0.00315 | 0.57002 | 0 | 0 | 0 |  | |  |  |
|  | RufiIndia | 5 | 0.0034 | 0.00341 | 0.0038 | -0.6765 | 2 | 1 | 1 |  | |  |  |
|  | RufiSEAsia | 2 | 0.00036 | 0.00036 | 0.00089 | -1.30048 | 2 | 1 | 1 |  | |  |  |
|  | Nivara | 0 | 0 | 0 | 0 | na | 0 | 0 | 0 |  | |  |  |
|  | Barthii | 0 | 0 | 0 | 0 | na | 0 | 0 | 0 |  | |  |  |
|  | Meridionalis | 0 | 0 | 0 | 0 | na | 0 | 0 | 0 |  | |  |  |
| **sts123** |  | # seg sites | pi silent | pi (JC) | theta silent | Taj D | polymor. loci | synonymous | replacement | |  | |  |
|  | SC | 0 | 0 | 0 | 0 | na | 0 | 0 | 0 |  | |  |  |
|  | ARred | 2 | 0.00017 | 0.00017 | 0.00105 | -1.16883 | 0 | 0 | 0 |  | |  |  |
|  | CAred | 0 | 0 | 0 | 0 | na | 0 | 0 | 0 |  | |  |  |
|  |  | 0 | 0 | 0 | 0 | na | x | x | x |  | |  |  |
|  | Tempjap | 0 | 0 | 0 | 0 | na | 0 | 0 | 0 |  | |  |  |
|  |  | 1 | 0.00061 | 0.00061 | 0.00073 | -0.30599 | x | x | x |  | |  |  |
|  |  | 1 | 0.00091 | 0.00091 | 0.001 | -0.27845 | x | x | x |  | |  |  |
|  | CAcult | 0 | 0 | 0 | 0 | na | 0 | 0 | 0 |  | |  |  |
|  | ARcult | 1 | 0.00087 | 0.00087 | 0.00081 | 0.13869 | 0 | 0 | 0 |  | |  |  |
|  |  | 0 | 0 | 0 | 0 | na | x | x | x |  | |  |  |
|  | indica | 1 | 0.00099 | 0.00099 | 0.00071 | 0.56544 | 0 | 0 | 0 |  | |  |  |
|  | SHWeedy | 0 | 0 | 0 | 0 | na | 0 | 0 | 0 |  | |  |  |
|  | MixWeedy | 1 | 0.0016 | 0.00161 | 0.00106 | 1.60435 | 0 | 0 | 0 |  | |  |  |
|  | BrHWeedy | 0 | 0 | 0 | 0 | na | 0 | 0 | 0 |  | |  |  |
|  | BHWeedy | 0 | 0 | 0 | 0 | na | 0 | 0 | 0 |  | |  |  |
|  | Rufi | 17 | 0.0057 | 0.00573 | 0.01134 | -0.97415 |  |  |  |  | |  |  |
|  |  | 1 | 0.00201 | 0.00201 | 0.00164 | 2.01187 | x | x | x |  | |  |  |
|  | Glumae | 0 | 0 | 0 | 0 | na | 0 | 0 | 0 |  | |  |  |
|  |  | 1 | 0.002 | 0.00201 | 0.00164 | 1.63299 | x | x | x |  | |  |  |
|  | glaber | 0 | 0 | 0 | 0 | na | 0 | 0 | 0 |  | |  |  |
|  |  | 1 | 0.00073 | 0.00073 | 0.00077 | -0.10825 | x | x | x |  | |  |  |
|  | PA_rice | 1 | 0.00201 | 0.00201 | 0.00164 |  | 0 | 0 | 0 |  | |  |  |
|  |  |  |  |  |  |  |  |  |  |  | |  |  |
|  | Updated Seq Set Analyses | | |  |  |  |  |  |  |  | |  |  |
|  |  |  |  |  |  |  |  |  |  |  | |  |  |
|  |  | # seg sites | pi silent | pi (JC) | theta silent | Taj D |  |  |  |  | |  |  |
|  | CAredWell | 0 | 0 | 0 | 0 | na | 0 | 0 | 0 |  | |  |  |
|  | DemmerWest1 | 0 | 0 | 0 | 0 | na | 0 | 0 | 0 |  | |  |  |
|  | DemmerWest2 | 0 | 0 | 0 | 0 | na | 0 | 0 | 0 |  | |  |  |
|  | DemmerEast | 0 | 0 | 0 | 0 | na | 0 | 0 | 0 |  | |  |  |
|  | TropJap | 1 | 0.00038 | 0.00038 | 0.00065 | -0.52602 | 0 | 0 | 0 |  | |  |  |
|  | Aromatic | 1 | 0.00091 | 0.00091 | 0.001 | -0.27845 | 0 | 0 | 0 |  | |  |  |
|  | Aus | 0 | 0 | 0 | 0 | na | 0 | 0 | 0 |  | |  |  |
|  | RufiChina | 11 | 0.01094 | 0.01102 | 0.00907 | 0.97473 | 0 | 0 | 0 |  | |  |  |
|  | RufiIndia | 12 | 0.00434 | 0.00435 | 0.01102 | -1.44774 | 0 | 0 | 0 |  | |  |  |
|  | RufiSEAsia | 3 | 0.00095 | 0.00095 | 0.0021 | -1.08539 | 0 | 0 | 0 |  | |  |  |
|  | Nivara | 1 | 0.0016 | 0.00161 | 0.00132 | 1.12414 | 0 | 0 | 0 |  | |  |  |
|  | Barthii | 3 | 0.00481 | 0.00483 | 0.00395 | 1.12414 | 0 | 0 | 0 |  | |  |  |
|  | Meridionalis | 3 | 0.00603 | 0.00606 | 0.00494 | 2.01187 | 0 | 0 | 0 |  | |  |  |
| **sts124** |  | # seg sites | pi silent | pi (JC) | theta silent | Taj D | polymor. loci | synonymous | replacement | |  | |  |
|  | SC | 0 | 0 | 0 | 0 | na | 0 | 0 | 0 |  | |  |  |
|  | ARred | 0 | 0 | 0 | 0 | na | 0 | 0 | 0 |  | |  |  |
|  | CAred | 0 | 0 | 0 | 0 | na | 0 | 0 | 0 |  | |  |  |
|  |  | 0 | 0 | 0 | 0 | na | x | x | x |  | |  |  |
|  | Tempjap | 0 | 0 | 0 | 0 | na | 0 | 0 | 0 |  | |  |  |
|  |  | 0 | 0 | 0 | 0 | na | x | x | x |  | |  |  |
|  |  | 0 | 0 | 0 | 0 | na | x | x | x |  | |  |  |
|  | CAcult | 0 | 0 | 0 | 0 | na | 0 | 0 | 0 |  | |  |  |
|  | ARcult | 0 | 0 | 0 | 0 | na | 0 | 0 | 0 |  | |  |  |
|  |  | 0 | 0 | 0 | 0 | na | x | x | x |  | |  |  |
|  | indica | 0 | 0 | 0 | 0 | na | 0 | 0 | 0 |  | |  |  |
|  | SHWeedy | 0 | 0 | 0 | 0 | na | 0 | 0 | 0 |  | |  |  |
|  | MixWeedy | 0 | 0 | 0 | 0 | na | 0 | 0 | 0 |  | |  |  |
|  | BrHWeedy | 0 | 0 | 0 | 0 | na | 0 | 0 | 0 |  | |  |  |
|  | BHWeedy | 0 | 0 | 0 | 0 | na | 0 | 0 | 0 |  | |  |  |
|  | Rufi | 13 | 0.00341 | 0.00342 | 0.00727 | -1.35663 |  |  |  |  | |  |  |
|  |  | 0 | 0 | 0 | 0 | na | x | x | x |  | |  |  |
|  | Glumae | 1 | 0.00224 | 0.00224 | 0.00183 | 1.63299 | 0 | 0 | 0 |  | |  |  |
|  |  | 0 | 0 | 0 | 0 | na | x | x | x |  | |  |  |
|  | glaber | 0 | 0 | 0 | 0 | na | 0 | 0 | 0 |  | |  |  |
|  |  | 0 | 0 | 0 | 0 | na | x | x | x |  | |  |  |
|  | PA_rice | 0 | 0 | 0 | 0 |  | 0 | 0 | 0 |  | |  |  |
|  |  |  |  |  |  |  |  |  |  |  | |  |  |
|  | Updated Seq Set Analyses | | |  |  |  |  |  |  |  | |  |  |
|  |  |  |  |  |  |  |  |  |  |  | |  |  |
|  |  | # seg sites | pi silent | pi (JC) | theta silent | Taj D |  |  |  |  | |  |  |
|  | CAredWell | 0 | 0 | 0 | 0 | na | 0 | 0 | 0 |  | |  |  |
|  | DemmerWest1 | 0 | 0 | 0 | 0 | na | 0 | 0 | 0 |  | |  |  |
|  | DemmerWest2 | 0 | 0 | 0 | 0 | na | 0 | 0 | 0 |  | |  |  |
|  | DemmerEast | 0 | 0 | 0 | 0 | na | 0 | 0 | 0 |  | |  |  |
|  | TropJap | 0 | 0 | 0 | 0 | na | 0 | 0 | 0 |  | |  |  |
|  | Aromatic | 0 | 0 | 0 | 0 | na | 0 | 0 | 0 |  | |  |  |
|  | Aus | 0 | 0 | 0 | 0 | na | 0 | 0 | 0 |  | |  |  |
|  | RufiChina | 5 | 0.00174 | 0.00175 | 0.00276 | -0.7761 | 2 | 0 | 2 |  | |  |  |
|  | RufiIndia | 8 | 0.00291 | 0.00291 | 0.00419 | -0.84586 | 4 | 1 | 3 |  | |  |  |
|  | RufiSEAsia | 10 | 0.00309 | 0.00309 | 0.00637 | -1.61811 | 2 | 0 | 2 |  | |  |  |
|  | Nivara | 1 | 0.0018 | 0.0018 | 0.00147 | 1.03194 | 0 | 0 | 0 |  | |  |  |
|  | Barthii | 0 | 0 | 0 | 0 | na | 0 | 0 | 0 |  | |  |  |
|  | Meridionalis | 0 | 0 | 0 | 0 | na | 0 | 0 | 0 |  | |  |  |
| **sts125** |  | # seg sites | pi silent | pi (JC) | theta silent | Taj D | polymor. loci | synonymous | replacement | |  | |  |
|  | SC | 0 | 0 | 0 | 0 | na | 0 | 0 | 0 |  | |  |  |
|  | ARred | 1 | 0.00008 | 0.00008 | 0.00065 | -0.89904 | 0 | 0 | 0 |  | |  |  |
|  | CAred | 0 | 0 | 0 | 0 | na | 0 | 0 | 0.00 |  | |  |  |
|  |  |  |  |  |  |  | x | x | x |  | |  |  |
|  | Tempjap | 0 | 0 | 0 | 0 | na | 0 | 0 | 0 |  | |  |  |
|  |  | 2 | 0.00117 | 0.00117 | 0.00181 | -0.68968 | x | x | x |  | |  |  |
|  |  | 0 | 0 | 0 | 0 | na | x | x | x |  | |  |  |
|  | CAcult | 0 | 0 | 0 | 0 | na | 0 | 0 | 0 |  | |  |  |
|  | ARcult | 1 | 0.0006 | 0.0006 | 0.001 | -0.68111 | 0 | 0.00 | 0 |  | |  |  |
|  |  | 1 | 0 | 0 | 0 | 0.01499 | x | x | x |  | |  |  |
|  | indica | 1 | 0.00171 | 0.00171 | 0.00087 | 1.35055 | 0 | 0.00 | 0 |  | |  |  |
|  | SHWeedy | 0 | 0 | 0 | 0 | na | 0 | 0 | 0 |  | |  |  |
|  | MixWeedy | 1 | 0.00133 | 0.00133 | 0.00133 | 0.01499 | 0 | 0.00 | 0 |  | |  |  |
|  | BrHWeedy | 0 | 0 | 0 | 0 | na | 0 | 0 | 0 |  | |  |  |
|  | BHWeedy | 0 | 0 | 0 | 0 | na | 0 | 0.00 | 0 |  | |  |  |
|  | Rufi | 14 | 0.00328 | 0.00329 | 0.01014 | -1.84373 |  |  |  |  | |  |  |
|  |  | 0 | 0 | 0 | 0 | na | x | x | x |  | |  |  |
|  | Glumae | 1 | 0.00252 | 0.00252 | 0.00206 | 1.89306 | 1 | 1 | 0 |  | |  |  |
|  |  | 5 | 0.0125 | 0.01261 | 0.01023 | 2.15629 | x | x | x |  | |  |  |
|  | glaber | 0 | 0 | 0 | 0 | na | 0 | 0 | 0 |  | |  |  |
|  |  |  |  |  |  |  | x | x | x |  | |  |  |
|  | PA_rice | 0.00 | 0.00 | 0.00 | 0.00 |  | 0 | 0 | 0 |  | |  |  |
|  |  |  |  |  |  |  |  |  |  |  | |  |  |
|  | Updated Seq Set Analyses | | |  |  |  |  |  |  |  | |  |  |
|  |  |  |  |  |  |  |  |  |  |  | |  |  |
|  |  | # seg sites | pi silent | pi (JC) | theta silent | Taj D |  |  |  |  | |  |  |
|  | CAredWell | 0 | 0 | 0 | 0 | na | 0 | 0 | 0 |  | |  |  |
|  | DemmerWest1 | 0 | 0 | 0 | 0 | na | 0 | 0 | 0 |  | |  |  |
|  | DemmerWest2 | 0 | 0 | 0 | 0 | na | 0 | 0 | 0 |  | |  |  |
|  | DemmerEast | 0 | 0 | 0 | 0 | na | 0 | 0 | 0 |  | |  |  |
|  | TropJap | 2 | 0.00081 | 0.00081 | 0.00177 | -0.93148 | 0 | 0 | 0 |  | |  |  |
|  | Aromatic | 0 | 0 | 0 | 0 | na | 0 | 0 | 0 |  | |  |  |
|  | Aus | 1 | 0 | 0 | 0 | -0.19492 | 1 | 0 | 1 |  | |  |  |
|  | RufiChina | 1 | 0.00034 | 0.00034 | 0.00103 | -1.1624 | 0 | 0 | 0 |  | |  |  |
|  | RufiIndia | 2 | 0.00069 | 0.00069 | 0.00186 | -1.2671 | 0 | 0 | 0 |  | |  |  |
|  | RufiSEAsia | 7 | 0.00564 | 0.00566 | 0.00627 | -0.28021 | 2 | 2 | 0 |  | |  |  |
|  | Nivara | 3 | 0.00607 | 0.00609 | 0.00498 | 1.12414 | 1 | 1 | 0 |  | |  |  |
|  | Barthii | 5 | 0.01 | 0.01007 | 0.00821 | 1.24649 | 1 | 1 | 0 |  | |  |  |
|  | Meridionalis | 1 | 0.0025 | 0.0025 | 0.00205 | 1.89306 | 1 | 0 | 1 |  | |  |  |

**Table D**. Standard sequence diversity indices (e.g. segregating sites, sequence diversity pi, Watterson’s theta, polymorphic loci, numbers of mutations, synonymous versus nonsynonymous replacements) for sequence tagged site (STS) loci in all groups of *Oryza* analyzed in this study.

| **Log Likelihood Values** | | |
| --- | --- | --- |
| ***K*** | **Mean** | **Variance** |
| 1 | 16957.606 | 207.155 |
| 2 | 13946.316 | 2331.923 |
| 3 | 11951.194 | 3341.237 |
| 4 | 10587.821 | 6125.332 |
| 5 | 10518.077 | 4892.519 |
| 6 | -9738.018 | 6430.001 |
| 7 | -9576.967 | 7962.029 |
| 8 | -9469.065 | 9129.998 |
| **9** | **-9448.274** | **11606.899** |
| 10 | -9480.589 | 13962.43 |
| 11 | -9463.621 | 18182.364 |
| 12 | -9499.871 | 21473.036 |
| 13 | -9480.022 | 24693.721 |
| 14 | -9887.324 | 39099.686 |
| 15 | -9498.861 | 36541.264 |
| 16 | -9509.78 | 44100.869 |
| 17 | -9544.792 | 50121.998 |
| 18 | -9529.199 | 57284.086 |
| 19 | -9937.281 | 81005.585 |
| 20 | -9547.536 | 73641.735 |
| 21 | -9562.89 | 84541.04 |
| 22 | -9970.413 | 115965.75 |

**Table E.** STRUCTURE mean log likelihood results. Mean log likelihood of each K value (LnP(K)) and variance are shown for all K cluster models evaluated in STRUCTURE. The most likely value of K is shown in bold.

| **Locus** | **Pop 1** | ***N*_1_** | **Pop 2** | ***N*_2_** | ***F_ST_*** | ***N_m_*** | ***D_a_*** |
| --- | --- | --- | --- | --- | --- | --- | --- |
| STS004 | CWR | 56 | CCR | 22 | 0.96364 | 0.01 | 0.00922 |
|  | CWR | 56 | SWR | 180 | 0.45890 | 0.29 | 0.00452 |
|  | CWR | 56 | SCR | 24 | 0.85751 | 0.04 | 0.00881 |
|  | CCR | 22 | SWR | 180 | 0.49701 | 0.25 | 0.00556 |
|  | CCR | 22 | SCR | 24 | 0.13043 | 1.67 | 0.00016 |
|  |  |  |  |  |  |  |  |
| STS005 | CWR | 56 | CCR | 22 | N/A* | N/A | 0.00000 |
|  | CWR | 56 | SWR | 186 | 0.84965 | 0.04 | 0.00177 |
|  | CWR | 56 | SCR | 24 | 0.04348 | 5.50 | 0.00001 |
|  | CCR | 22 | SWR | 186 | 0.84865 | 0.04 | 0.00177 |
|  | CCR | 22 | SCR | 24 | 0.04348 | 5.50 | 0.00001 |
|  |  |  |  |  |  |  |  |
| STS007 | CWR | 56 | CCR | 22 | 0.88472 | 0.03 | 0.00203 |
|  | CWR | 56 | SWR | 186 | -0.01192 | -21.22 | 0.00000 |
|  | CWR | 56 | SCR | 24 | 0.77766 | 0.07 | 0.00137 |
|  | CCR | 22 | SWR | 186 | 0.88857 | 0.03 | 0.00218 |
|  | CCR | 22 | SCR | 24 | 0.10121 | 2.22 | 0.00006 |
|  | SWR | 186 | SCR | 24 | 0.78282 | 0.07 | 0.00147 |
| STS011 | CWR | 56 | CCR | 22 | N/A | N/A | 0.00000 |
|  | CWR | 56 | SWR | 186 | N/A | N/A | 0.00000 |
|  | CWR | 56 | SCR | 24 | N/A | N/A | 0.00000 |
|  | CCR | 22 | SWR | 186 | N/A | N/A | 0.00000 |
|  | CCR | 22 | SCR | 24 | N/A | N/A | 0.00000 |
|  |  |  |  |  |  |  |  |
| STS012 | CWR | 56 | CCR | 22 | N/A | N/A | 0.00000 |
|  | CWR | 56 | SWR | 186 | N/A | N/A | 0.00000 |
|  | CWR | 56 | SCR | 24 | N/A | N/A | 0.00000 |
|  | CCR | 22 | SWR | 186 | N/A | N/A | 0.00000 |
|  | CCR | 22 | SCR | 24 | N/A | N/A | 0.00000 |
|  |  |  |  |  |  |  |  |
| STS021 | CWR | 56 | CCR | 22 | 0.90476 | 0.03 | 0.00523 |
|  | CWR | 56 | SWR | 184 | 0.02368 | 10.31 | 0.00002 |
|  | CWR | 56 | SCR | 24 | 0.60641 | 0.16 | 0.01277 |
|  | CCR | 22 | SWR | 184 | 0.78214 | 0.07 | 0.00548 |
|  | CCR | 22 | SCR | 24 | 0.64189 | 0.14 | 0.01579 |
|  |  |  |  |  |  |  |  |
| **Locus** | **Pop 1** | ***N*_1_** | **Pop 2** | ***N*_2_** | ***F_ST_*** | ***N_m_*** | ***D_a_*** |
| STS023 | CWR | 56 | CCR | 22 | 0.96364 | 0.01 | 0.00424 |
|  | CWR | 56 | SWR | 186 | 0.81906 | 0.06 | 0.00213 |
|  | CWR | 56 | SCR | 24 | 0.76842 | 0.08 | 0.00357 |
|  | CCR | 22 | SWR | 186 | 0.95322 | 0.01 | 0.00648 |
|  | CCR | 22 | SCR | 24 | 0.04348 | 5.50 | 0.00004 |
|  |  |  |  |  |  |  |  |
| STS024 | CWR | 56 | CCR | 22 | N/A | N/A | 0.00000 |
|  | CWR | 56 | SWR | 186 | 0.93822 | 0.02 | 0.00384 |
|  | CWR | 56 | SCR | 24 | 0.10145 | 2.21 | 0.00006 |
|  | CCR | 22 | SWR | 186 | 0.93822 | 0.02 | 0.00385 |
|  | CCR | 22 | SCR | 24 | 0.10145 | 2.21 | 0.00006 |
|  |  |  |  |  |  |  |  |
| STS025 | CWR | 56 | CCR | 22 | N/A | N/A | 0.00000 |
|  | CWR | 56 | SWR | 186 | 0.87034 | 0.04 | 0.00186 |
|  | CWR | 56 | SCR | 24 | 0.04348 | 5.50 | 0.00001 |
|  | CCR | 22 | SWR | 186 | 0.87034 | 0.04 | 0.00178 |
|  | CCR | 22 | SCR | 24 | 0.04348 | 5.50 | 0.00001 |
|  |  |  |  |  |  |  |  |
| STS031 | CWR | 56 | CCR | 22 | 0.96364 | 0.01 | 0.00445 |
|  | CWR | 56 | SWR | 186 | 0.01891 | 12.97 | 0.00001 |
|  | CWR | 56 | SCR | 24 | 0.77766 | 0.07 | 0.00157 |
|  | CCR | 22 | SWR | 186 | 0.89034 | 0.03 | 0.00408 |
|  | CCR | 22 | SCR | 24 | 0.13043 | 1.67 | 0.00005 |
|  |  |  |  |  |  |  |  |
| STS035 | CWR | 56 | CCR | 22 | 0.30493 | 0.57 | 0.00041 |
|  | CWR | 56 | SWR | 186 | 0.03546 | 6.80 | 0.00002 |
|  | CWR | 56 | SCR | 24 | 0.56594 | 0.19 | 0.00125 |
|  | CCR | 22 | SWR | 186 | 0.21191 | 0.93 | 0.00033 |
|  | CCR | 22 | SCR | 24 | 0.08333 | 2.75 | 0.00016 |
|  |  |  |  |  |  |  |  |
| STS036 | CWR | 56 | CCR | 22 | 0.76000 | 0.08 | 0.00131 |
|  | CWR | 56 | SWR | 186 | 0.38702 | 0.40 | 0.00065 |
|  | CWR | 56 | SCR | 24 | 0.48622 | 0.26 | 0.00071 |
|  | CCR | 22 | SWR | 186 | 0.54667 | 0.21 | 0.00158 |
|  | CCR | 22 | SCR | 24 | 0.04698 | 5.07 | 0.00005 |
|  |  |  |  |  |  |  |  |
|  |  |  |  |  |  |  |  |
| **Locus** | **Pop 1** | ***N*_1_** | **Pop 2** | ***N*_2_** | ***F_ST_*** | ***N_m_*** | ***D_a_*** |
| STS040 | CWR | 56 | CCR | 22 | 0.96364 | 0.01 | 0.00442 |
|  | CWR | 56 | SCR | 24 | 0.96364 | 0.01 | 0.00438 |
|  | CCR | 22 | SWR | 186 | N/A | N/A | 0.00000 |
|  | CCR | 22 | SCR | 24 | 0.04348 | 5.50 | 0.00001 |
|  |  |  |  |  |  |  |  |
| STS041 | CWR | 56 | CCR | 22 | 0.71207 | 0.10 | 0.00225 |
|  | CWR | 56 | SWR | 182 | N/A | N/A | 0.00000 |
|  | CWR | 56 | SCR | 24 | 0.13866 | 1.55 | 0.00016 |
|  | CCR | 22 | SWR | 182 | 0.76471 | 0.08 | 0.00239 |
|  | CCR | 22 | SCR | 24 | 0.40683 | 0.36 | 0.00111 |
|  |  |  |  |  |  |  |  |
| STS046 | CWR | 56 | CCR | 22 | 0.86175 | 0.04 | 0.00182 |
|  | CWR | 56 | SWR | 186 | 0.93582 | 0.02 | 0.00259 |
|  | CWR | 56 | SCR | 24 | 0.81114 | 0.06 | 0.00200 |
|  | CCR | 22 | SWR | 186 | 0.93726 | 0.02 | 0.00469 |
|  | CCR | 22 | SCR | 24 | 0.86003 | 0.04 | 0.00339 |
|  |  |  |  |  |  |  |  |
| STS047 | CWR | 56 | CCR | 22 | 0.78642 | 0.07 | 0.00391 |
|  | CWR | 56 | SWR | 186 | 0.80472 | 0.06 | 0.00200 |
|  | CWR | 56 | SCR | 24 | 0.73537 | 0.09 | 0.00271 |
|  | CCR | 22 | SWR | 186 | 0.63957 | 0.14 | 0.00206 |
|  | CCR | 22 | SCR | 24 | 0.26341 | 0.70 | 0.00058 |
|  |  |  |  |  |  |  |  |
| STS051 | CWR | 56 | CCR | 22 | 0.75067 | 0.08 | 0.00332 |
|  | CWR | 56 | SWR | 186 | 0.84741 | 0.05 | 0.00383 |
|  | CWR | 56 | SCR | 24 | 0.72654 | 0.09 | 0.00328 |
|  | CCR | 22 | SWR | 186 | 0.54584 | 0.21 | 0.00175 |
|  | CCR | 22 | SCR | 24 | -0.03638 | -7.12 | -0.00007 |
|  |  |  |  |  |  |  |  |
| STS052 | CWR | 56 | CCR | 22 | 0.96364 | 0.01 | 0.00201 |
|  | CWR | 56 | SWR | 184 | 0.70892 | 0.10 | 0.00164 |
|  | CWR | 56 | SCR | 24 | 0.96364 | 0.01 | 0.00202 |
|  | CCR | 22 | SWR | 184 | 0.14228 | 1.51 | 0.00010 |
|  | CCR | 22 | SCR | 24 | N/A | N/A | 0.00000 |
|  |  |  |  |  |  |  |  |

| **Locus** | **Pop 1** | ***N*_1_** | **Pop 2** | ***N*_2_** | ***F_ST_*** | ***N_m_*** | ***D_a_*** |
| --- | --- | --- | --- | --- | --- | --- | --- |
| STS059 | CWR | 56 | CCR | 22 | N/A | N/A | 0.00000 |
|  | CWR | 56 | SWR | 186 | N/A | N/A | 0.00000 |
|  | CWR | 56 | SCR | 24 | 0.04348 | 5.50 | 0.00001 |
|  | CCR | 22 | SWR | 186 | N/A | N/A | 0.00000 |
|  | CCR | 22 | SCR | 24 | 0.04348 | 5.50 | 0.00001 |
|  |  |  |  |  |  |  |  |
| STS060 | CWR | 56 | CCR | 22 | 0.70014 | 0.11 | 0.00501 |
|  | CWR | 56 | SWR | 186 | 0.89013 | 0.03 | 0.00408 |
|  | CWR | 56 | SCR | 24 | 0.65815 | 0.13 | 0.00503 |
|  | CCR | 22 | SWR | 186 | 0.52600 | 0.23 | 0.00218 |
|  | CCR | 22 | SCR | 24 | 0.04230 | 5.66 | 0.00018 |
|  |  |  |  |  |  |  |  |
| STS061 | CWR | 56 | CCR | 22 | 0.96364 | 0.01 | 0.00209 |
|  | CWR | 56 | SWR | 186 | -0.00966 | -26.12 | 0.00000 |
|  | CWR | 56 | SCR | 24 | 0.77766 | 0.07 | 0.00151 |
|  | CCR | 22 | SWR | 186 | 0.97838 | 0.01 | 0.00230 |
|  | CCR | 22 | SCR | 24 | 0.13043 | 1.67 | 0.00005 |
|  |  |  |  |  |  |  |  |
| STS063 | CWR | 56 | CCR | 22 | N/A | N/A | 0.00000 |
|  | CWR | 56 | SWR | 186 | N/A | N/A | 0.00000 |
|  | CWR | 56 | SCR | 24 | N/A | N/A | 0.00000 |
|  | CCR | 22 | SWR | 186 | N/A | N/A | 0.00000 |
|  | CCR | 22 | SCR | 24 | N/A | N/A | 0.00000 |
|  |  |  |  |  |  |  |  |
| STS065 | CWR | 56 | CCR | 22 | 0.96394 | 0.01 | 0.00483 |
|  | CWR | 56 | SWR | 186 | 0.96364 | 0.01 | 0.00469 |
|  | CWR | 56 | SCR | 24 | 0.84674 | 0.05 | 0.00444 |
|  | CCR | 22 | SWR | 186 | N/A | N/A | 0.00000 |
|  | CCR | 22 | SCR | 24 | 0.04348 | 5.50 | 0.00003 |
|  |  |  |  |  |  |  |  |
| STS066 | CWR | 56 | CCR | 22 | N/A | N/A | 0.00000 |
|  | CWR | 56 | SWR | 186 | N/A | N/A | 0.00000 |
|  | CWR | 56 | SCR | 24 | 0.04348 | 5.50 | 0.00001 |
|  | CCR | 22 | SWR | 186 | N/A | N/A | 0.00000 |
|  | CCR | 22 | SCR | 24 | 0.04348 | 5.50 | 0.00001 |
|  |  |  |  |  |  |  |  |

| **Locus** | **Pop 1** | ***N*_1_** | **Pop 2** | ***N*_2_** | ***F_ST_*** | ***N_m_*** | ***D_a_*** |
| --- | --- | --- | --- | --- | --- | --- | --- |
| STS068 | CWR | 56 | CCR | 22 | N/A | N/A | 0.00000 |
|  | CWR | 56 | SWR | 186 | N/A | N/A | 0.00000 |
|  | CWR | 56 | SCR | 24 | 0.04348 | 5.50 | 0.00001 |
|  | CCR | 22 | SWR | 186 | N/A | N/A | 0.00000 |
|  | CCR | 22 | SCR | 24 | 0.04348 | 5.50 | 0.00001 |
|  |  |  |  |  |  |  |  |
| STS070 | CWR | 56 | CCR | 22 | N/A | N/A | 0.00000 |
|  | CWR | 56 | SWR | 186 | 0.05946 | 3.95 | 0.00001 |
|  | CWR | 56 | SCR | 22 | N/A | N/A | 0.00000 |
|  | CCR | 22 | SWR | 186 | 0.05946 | 3.95 | 0.00001 |
|  | CCR | 22 | SCR | 22 | N/A | N/A | 0.00000 |
|  |  |  |  |  |  |  |  |
| STS071 | CWR | 56 | CCR | 20 | 0.90672 | 0.03 | 0.03777 |
|  | CWR | 56 | SWR | 184 | 0.92495 | 0.02 | 0.03339 |
|  | CWR | 56 | SCR | 22 | 0.35843 | 0.45 | 0.00796 |
|  | CCR | 20 | SWR | 184 | 0.32323 | 0.52 | 0.00129 |
|  | CCR | 20 | SCR | 22 | 0.44474 | 0.31 | 0.01212 |
|  |  |  |  |  |  |  |  |
| STS072 | CWR | 56 | CCR | 22 | 0.86175 | 0.04 | 0.00877 |
|  | CWR | 56 | SWR | 186 | -0.01085 | -23.28 | -0.00001 |
|  | CWR | 56 | SCR | 22 | 0.84125 | 0.05 | 0.00851 |
|  | CCR | 22 | SWR | 186 | 0.85571 | 0.04 | 0.00642 |
|  | CCR | 22 | SCR | 22 | -0.03611 | -7.17 | -0.00008 |
|  |  |  |  |  |  |  |  |
| STS073 | CWR | 56 | CCR | 22 | 0.71429 | 0.10 | 0.00450 |
|  | CWR | 56 | SWR | 182 | N/A | N/A | 0.00000 |
|  | CWR | 56 | SCR | 24 | N/A | N/A | 0.00000 |
|  | CCR | 22 | SWR | 182 | 0.71429 | 0.10 | 0.00450 |
|  | CCR | 22 | SCR | 24 | 0.71429 | 0.10 | 0.00449 |
|  |  |  |  |  |  |  |  |
| STS080 | CWR | 56 | CCR | 22 | 0.96364 | 0.01 | 0.00229 |
|  | CWR | 56 | SWR | 186 | 0.92814 | 0.02 | 0.00478 |
|  | CWR | 56 | SCR | 24 | 0.89044 | 0.03 | 0.00230 |
|  | CCR | 22 | SWR | 186 | 0.89599 | 0.03 | 0.00242 |
|  | CCR | 22 | SCR | 24 | 0.04348 | 5.50 | 0.00001 |
|  |  |  |  |  |  |  |  |

| **Locus** | **Pop 1** | ***N*_1_** | **Pop 2** | ***N*_2_** | ***F_ST_*** | ***N_m_*** | ***D_a_*** |
| --- | --- | --- | --- | --- | --- | --- | --- |
| STS082 | CWR | 56 | CCR | 22 | N/A | N/A | 0.00000 |
|  | CWR | 56 | SWR | 186 | N/A | N/A | 0.00000 |
|  | CWR | 56 | SCR | 24 | N/A | N/A | 0.00000 |
|  | CCR | 22 | SWR | 186 | N/A | N/A | 0.00000 |
|  | CCR | 22 | SCR | 24 | N/A | N/A | 0.00000 |
|  |  |  |  |  |  |  |  |
| STS083 | CWR | 56 | CCR | 22 | 0.96364 | 0.01 | 0.00431 |
|  | CWR | 56 | SWR | 184 | 0.96223 | 0.01 | 0.01638 |
|  | CWR | 56 | SCR | 24 | 0.11985 | 1.84 | 0.00041 |
|  | CCR | 22 | SWR | 184 | 0.97096 | 0.01 | 0.02055 |
|  | CCR | 22 | SCR | 24 | 0.39799 | 0.38 | 0.00201 |
|  |  |  |  |  |  |  |  |
| STS085 | CWR | 56 | CCR | 22 | 0.77866 | 0.07 | 0.00865 |
|  | CWR | 56 | SWR | 186 | 0.90594 | 0.03 | 0.00773 |
|  | CWR | 56 | SCR | 24 | 0.72239 | 0.10 | 0.00705 |
|  | CCR | 22 | SWR | 186 | 0.47986 | 0.27 | 0.00173 |
|  | CCR | 22 | SCR | 24 | 0.17777 | 1.16 | 0.00083 |
|  |  |  |  |  |  |  |  |
| STS086 | CWR | 56 | CCR | 22 | 0.83883 | 0.05 | 0.00355 |
|  | CWR | 56 | SWR | 186 | 0.90134 | 0.03 | 0.00400 |
|  | CWR | 56 | SCR | 24 | 0.77832 | 0.07 | 0.00334 |
|  | CCR | 22 | SWR | 186 | 0.87275 | 0.04 | 0.00355 |
|  | CCR | 22 | SCR | 24 | -0.01863 | -13.67 | -0.00002 |
|  |  |  |  |  |  |  |  |
| STS087 | CWR | 56 | CCR | 22 | N/A | N/A | 0.00000 |
|  | CWR | 56 | SWR | 186 | N/A | N/A | 0.00000 |
|  | CWR | 56 | SCR | 24 | N/A | N/A | 0.00000 |
|  | CCR | 22 | SWR | 186 | N/A | N/A | 0.00000 |
|  | CCR | 22 | SCR | 24 | N/A | N/A | 0.00000 |
|  |  |  |  |  |  |  |  |
| STS089 | CWR | 56 | CCR | 22 | N/A | N/A | 0.00000 |
|  | CWR | 56 | SWR | 186 | 0.65714 | 0.13 | 0.01303 |
|  | CWR | 56 | SCR | 24 | 0.30435 | 0.57 | 0.00246 |
|  | CCR | 22 | SWR | 186 | 0.65714 | 0.13 | 0.01299 |
|  | CCR | 22 | SCR | 24 | 0.30435 | 0.57 | 0.00246 |
|  |  |  |  |  |  |  |  |
| **Locus** | **Pop 1** | ***N*_1_** | **Pop 2** | ***N*_2_** | ***F_ST_*** | ***N_m_*** | ***D_a_*** |
| STS090 | CWR | 56 | CCR | 22 | 0.96364 | 0.01 | 0.00653 |
|  | CWR | 56 | SWR | 186 | 0.68519 | 0.11 | 0.00215 |
|  | CWR | 56 | SCR | 24 | 0.75330 | 0.08 | 0.00445 |
|  | CCR | 22 | SWR | 186 | 0.92126 | 0.02 | 0.00894 |
|  | CCR | 22 | SCR | 24 | 0.11801 | 1.87 | 0.00016 |
|  |  |  |  |  |  |  |  |
| STS099 | CWR | 56 | CCR | 22 | 0.96364 | 0.01 | 0.00465 |
|  | CWR | 56 | SWR | 186 | 0.75930 | 0.08 | 0.00406 |
|  | CWR | 56 | SCR | 24 | 0.96364 | 0.01 | 0.00466 |
|  | CCR | 22 | SWR | 186 | 0.18919 | 1.07 | 0.00025 |
|  | CCR | 22 | SCR | 24 | N/A | N/A | 0.00000 |
|  |  |  |  |  |  |  |  |
| STS102 | CWR | 56 | CCR | 22 | N/A | N/A | 0.00000 |
|  | CWR | 56 | SWR | 186 | N/A | N/A | 0.00000 |
|  | CWR | 56 | SCR | 24 | 0.04348 | 5.50 | 0.00001 |
|  | CCR | 22 | SWR | 186 | N/A | N/A | 0.00000 |
|  | CCR | 22 | SCR | 24 | 0.04348 | 5.50 | 0.00001 |
|  |  |  |  |  |  |  |  |
| STS104 | CWR | 56 | CCR | 22 | 0.96364 | 0.01 | 0.00230 |
|  | CWR | 56 | SWR | 186 | -0.01192 | -21.22 | 0.00000 |
|  | CWR | 56 | SCR | 24 | 0.87059 | 0.04 | 0.00201 |
|  | CCR | 22 | SWR | 186 | 0.96757 | 0.01 | 0.00232 |
|  | CCR | 22 | SCR | 24 | 0.04348 | 5.50 | 0.00001 |
|  |  |  |  |  |  |  |  |
| STS108 | CWR | 56 | CCR | 22 | 0.96364 | 0.01 | 0.00233 |
|  | CWR | 56 | SWR | 186 | 0.96364 | 0.01 | 0.00248 |
|  | CWR | 56 | SCR | 24 | 0.96364 | 0.01 | 0.00233 |
|  | CCR | 22 | SWR | 186 | N/A | N/A | 0.00000 |
|  | CCR | 22 | SCR | 24 | N/A | N/A | 0.00000 |
|  |  |  |  |  |  |  |  |
| STS113 | CWR | 56 | CCR | 20 | 0.96364 | 0.01 | 0.00195 |
|  | CWR | 56 | SWR | 178 | -0.01012 | -24.96 | 0.00000 |
|  | CWR | 56 | SCR | 24 | 0.87059 | 0.04 | 0.00169 |
|  | CCR | 20 | SWR | 186 | 0.95480 | 0.01 | 0.00210 |
|  | CCR | 20 | SCR | 24 | 0.04348 | 5.50 | 0.00001 |
|  |  |  |  |  |  |  |  |
| **Locus** | **Pop 1** | ***N*_1_** | **Pop 2** | ***N*_2_** | ***F_ST_*** | ***N_m_*** | ***D_a_*** |
| STS116 | CWR | 56 | CCR | 22 | 0.96364 | 0.01 | 0.00226 |
|  | CWR | 56 | SWR | 126 | 0.94649 | 0.01 | 0.00241 |
|  | CWR | 56 | SCR | 22 | 0.80367 | 0.06 | 0.00259 |
|  | CCR | 22 | SWR | 126 | 0.00800 | 31.00 | 0.00000 |
|  | CCR | 22 | SCR | 22 | 0.23810 | 0.80 | 0.00017 |
|  |  |  |  |  |  |  |  |
| STS120 | CWR | 56 | CCR | 22 | N/A | N/A | 0.00000 |
|  | CWR | 56 | SWR | 186 | N/A | N/A | 0.00000 |
|  | CWR | 56 | SCR | 24 | N/A | N/A | 0.00000 |
|  | CCR | 22 | SWR | 186 | N/A | N/A | 0.00000 |
|  | CCR | 22 | SCR | 24 | N/A | N/A | 0.00000 |
|  |  |  |  |  |  |  |  |
| STS121 | CWR | 56 | CCR | 22 | 0.96364 | 0.01 | 0.00214 |
|  | CWR | 56 | SWR | 186 | 0.96364 | 0.01 | 0.00222 |
|  | CWR | 56 | SCR | 24 | 0.96364 | 0.01 | 0.00214 |
|  | CCR | 22 | SWR | 186 | N/A | N/A | 0.00000 |
|  | CCR | 22 | SCR | 24 | N/A | N/A | 0.00000 |
|  |  |  |  |  |  |  |  |
| STS123 | CWR | 56 | CCR | 22 | 0.96364 | 0.01 | 0.00214 |
|  | CWR | 56 | SWR | 186 | -0.00668 | -37.66 | 0.00000 |
|  | CWR | 56 | SCR | 24 | 0.77766 | 0.07 | 0.00150 |
|  | CCR | 22 | SWR | 186 | 0.96257 | 0.01 | 0.00229 |
|  | CCR | 22 | SCR | 24 | 0.13043 | 1.67 | 0.00005 |
|  |  |  |  |  |  |  |  |
| STS124 | CWR | 56 | CCR | 22 | 0.96364 | 0.01 | 0.00453 |
|  | CWR | 56 | SWR | 186 | 0.96364 | 0.01 | 0.00246 |
|  | CWR | 56 | SCR | 24 | 0.84333 | 0.05 | 0.00344 |
|  | CCR | 22 | SWR | 186 | N/A | N/A | 0.00000 |
|  | CCR | 22 | SCR | 24 | 0.21739 | 0.90 | 0.00013 |
|  |  |  |  |  |  |  |  |
| STS125 | CWR | 56 | CCR | 22 | N/A | N/A | 0.00000 |
|  | CWR | 56 | SWR | 186 | 0.00541 | 46.00 | 0.00000 |
|  | CWR | 56 | SCR | 24 | 0.04348 | 5.50 | 0.00001 |
|  | CCR | 22 | SWR | 186 | 0.00541 | 46.00 | 0.00000 |
|  | CCR | 22 | SCR | 24 | 0.04348 | 5.50 | 0.00001 |
|  |  |  |  |  |  |  |  |

| **Locus** | **Pop 1** | ***N*_1_** | **Pop 2** | ***N*_2_** | ***F_ST_*** | ***N_m_*** | ***D_a_*** |
| --- | --- | --- | --- | --- | --- | --- | --- |
| **Average STS** | CWR | 56 | CCR | 22 | 0.87849 | 0.04938 | 0.00323 |
|  | CWR | 56 | SWR | 184 | 0.58558 | -2.02306 | 0.00277 |
|  | CWR | 56 | SCR | 24 | 0.59231 | 1.17800 | 0.00234 |
|  | CCR | 22 | SWR | 184 | 0.66058 | 2.63758 | 0.00246 |
|  | CCR | 22 | SCR | 24 | 0.16638 | 2.03946 | 0.00092 |
|  |  |  |  |  |  |  |  |

**Table F**. Divergence measures for 48 STS loci. Estimates of gene flow (*F_ST_*), number of migrants (*Nm*), and number of net nucleotide substitutions per site between populations (*Da*). *N* is the number of individuals. *No polymorphic sites in the region. CWR indicates California weedy rice (*O. sativa*), CCR indicates California cultivated rice, Population 1 (Pop 1) is intraspecific and Population 2 (Pop 2) is interspecific.
